# Efficient and Rapid Arylation of NH₂‐Unprotected Bromobisindole Ethanamines via Suzuki‐Miyaura Coupling: Generating New Leads Against *Leishmania*

**DOI:** 10.1002/chem.202500637

**Published:** 2025-05-19

**Authors:** Alessandro Buono, Aurora Diotallevi, Sara Maestrini, Michele Verboni, Paula Kiuru, Luca Galluzzi, Andrea Duranti, Diego Olivieri, Simone Lucarini

**Affiliations:** ^1^ Department of Biomolecular Sciences Section of Chemistry and Pharmaceutical Technologies University of Urbino Carlo Bo Campus Scientifico E. Mattei, via Ca’ le suore 2 Urbino 61029 Italy; ^2^ Department of Biomolecular Sciences Section of Biochemistry and Biotechnology University of Urbino Carlo Bo Via Arco d'Augusto 2 Fano 61032 Italy; ^3^ Drug Research Program, Division of Pharmaceutical Chemistry and Technology, Faculty of Pharmacy University of Helsinki Viikinkaari 5E, P.O. Box 56 Helsinki 00014 Finland

**Keywords:** bisindoles, late‐stage derivatization, *Leishmania*, palladium, Suzuki‐Miyaura coupling

## Abstract

Leishmaniasis is a neglected tropical disease which presents significant global health challenges due to the lack of effective vaccines and the limitations of existing chemotherapeutics in view of their toxicity, resistance, and high costs. In this study, we realized a library of novel bisindole derivatives as potential antileishmanial agents through a rapid Suzuki‐Miyaura coupling reaction, utilizing NH_2_‐unprotected bromobisindole ethanamines and boronic acids. Optimization of reaction conditions allowed for the efficient and selective arylation of these substrates, with yields up to 93%. The compounds were screened for their activity against *Leishmania infantum* promastigotes. Among the tested bisindole derivatives, **3af** (bearing a 4‐vinylphenyl moiety) demonstrated potent antileishmanial activity (IC_50_ = 1.1 µM) with a higher selectivity index (21.8) compared to the reference drug miltefosine (9.8). A significant activity was also retained against intracellular amastigotes. This study establishes a robust methodology for late‐stage functionalization of bisindoles, also highlighting these derivatives' potential as promising leads for antileishmanial drug development.

## Graphical abstract

A library of novel bisindole derivatives was synthesized through an optimized rapid Suzuki‐Miyaura coupling reaction, utilizing NH_2_‐unprotected bromobisindole ethanamines and boronic acids with yields up to 93%. The compounds were screened for their activity against *L. infantum* promastigotes. In particular, bisindole **3af** demonstrated potent antileishmanial activity with a higher selectivity index compared to the reference drug miltefosine.

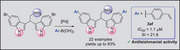

## 1 Introduction

Leishmaniasis is the second most dangerous parasitic disease, after malaria, in terms of mortality and morbidity. Up to 12 million poverty stricken people are infected and more than one million new cases are believed to occur every year worldwide.^[^ ^1^, ^2^, ^3^ ^]^ The World Health Organization also estimates that nearly one billion people in large parts of Africa, the Middle East, South America, and Asia are at risk of infection.^[^ ^4^ ^]^ Leishmaniasis is endemic also within the Mediterranean basin, and recent evidence suggests that the disease has expanded mainly due to climate change (affecting distribution of sandfly vectors and reservoir hosts), travels, and migration.^[^ ^5^ ^]^ Leishmaniasis is a neglected tropical disease caused by more than 20 species of protozoan *Leishmania*, which is transmitted to humans by the bite of infected female sandflies. This disease presents different clinical manifestations: visceral (VL), mucocutaneous (ML), and cutaneous (CL). VL, also known as kala‐azar, affects internal organs (bone marrow, liver, and spleen), and is fatal if untreated. ML can lead to the partial or total destruction of the nose, mouth, and throat mucous membranes. The CL form is the most common variety of leishmaniasis and results in ulcers and skin lesions. The VL and CL forms are prevalent in Mediterranean Europe and are caused by *L. infantum* protozoa.^[^ ^6^ ^]^

Today, no effective vaccines are available on the market^[^ ^7^ ^]^ and commercial chemotherapeutics present several drawbacks such as toxicity and multiorgan side effects, resistance, lack of specificity, prolonged treatment, high cost, and administration route.^[^ ^8^ ^]^ Therefore, the discovery of new potential drug candidates against leishmaniasis is an important goal for the research community.

**Figure 1:**
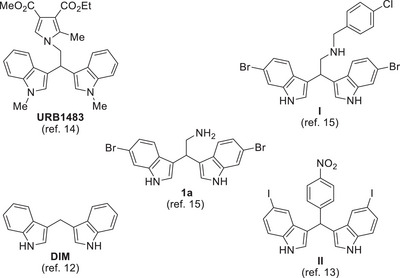
Bisindoles with antileishmanial activity.

Among several new active heterocyclic scaffolds,^[^ ^9^, ^10^, ^11^ ^]^ some bisindoles were reported to possess interesting antileishmanial activity (Figure 1).

3,3′‐Diindolylmethane (DIM, Figure 1) was the first antileishmanial bisindole reported in 2008 by Roy et al., who demonstrated that DIM is a potent *L. donovani* DNA topoisomerase I poison (IC_50_ = 1.2 µM).^[^ ^12^ ^]^ The methylene linker of DIM could be an ideal point of chemical diversification to generate new active compounds. This strategy has also been explored by Bharate and coworkers,^[^ ^13^ ^]^ who used a phenyl ring as an additional side arm. In detail, **II** (Figure 1) was the most effective compound of the new aryl‐DIM class against *L. donovani*, showing IC_50_ value lower than 10 µM. However, they did not show any toxicity data on human cells for all the reported compounds.^[^ ^13^ ^]^

**Scheme 1:**
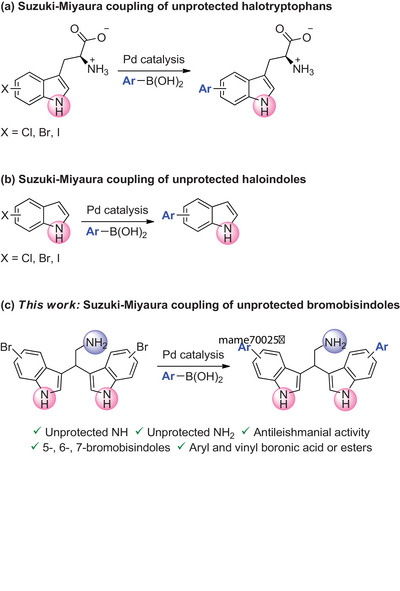
Suzuki‐Miyaura coupling of unprotected (a) halotryptophans, (b) haloindoles, and (c) bromobisindoles.

URB1483 (Figure 1), the most promising pyrrole‐bisindole reported by our group, showed excellent in vitro results such as potency against *L. infantum* promastigotes (IC_50_ = 3.7 µM) and intracellular amastigotes with no quantifiable cytotoxicity in mammalian cells (selectivity index [SI] > 55).^[^ ^14^ ^]^ However, URB1483 failed the in vivo studies on a mouse infection model probably due to its poor water solubility.^[^ ^15^ ^]^ Consequently, we designed and synthesized more hydrophilic bisindoles, and compound **I** (Figure 1) was the most potent against *L. infantum* promastigotes with IC_50_ = 2.7 µM, however slightly toxic on human macrophage‐type THP‐1 cell (CC_50_ = 11.7 µM) thus showing low SI (SI ≈ 4).^[^ ^15^ ^]^ Marine bisindole alkaloid 2,2‐bis(6‐bromo‐1*H*‐indol‐3‐yl) ethanamine (**1a**, Figure 1) was also active against *L. infantum* but resulted toxic (4 < CC_50_ < 20 µM).^[^ ^15^ ^]^ We speculated that their toxicity was due to the presence of bromine atoms in the indole rings compared with similar bisindoles. Therefore, selective late‐stage functionalizations for replacing the bromine atoms of these molecules can possibly lead to a series of nontoxic derivatives possessing antileishmanial activity. To pursue our goal, using an appropriate nucleophile, a series of cross‐coupling reactions can be performed.^[^ ^16^ ^]^ Furthermore, Suzuki‐Miyaura coupling^[^ ^17^ ^]^ allows the facile insertion of aryl functionalities starting from cheap and easily available boronic acids.^[^ ^18^ ^]^ Usually, NH‐unprotected haloindole derivatives are challenging substrates for metal‐catalyzed cross‐coupling reactions since the nitrogen, coordinating to the metal,^[^ ^19^ ^]^ can lead to undesired side reactions eventually lowering the amount of the desired product; therefore, protective groups are necessary.^[^ ^20^ ^]^ In 2008, Goss and coworkers reported the first example of palladium (Pd)‐catalyzed Suzuki‐Miyaura coupling of unprotected halotryptophans, using water as solvent^[^ ^21^ ^]^ (Scheme 1a), and successively they utilized these substrates for other Pd‐catalyzed cross‐coupling reactions.^[^ ^22^ ^]^ More recently, the same group succeeded in the coupling of unprotected haloindoles with aryl boronic acids (Scheme 1b).^[^ ^23^ ^]^ Despite the importance of all these and others’ contributions in the field,^[^ ^24^ ^]^ to the best of our knowledge, no examples of the Suzuki‐Miyaura coupling have been reported with halotryptamines,^[^ ^25^ ^]^ which exhibit both the indole and the amino nitrogen atoms unprotected, as in the case of the marine bisindole alkaloid **1a**. Indeed, also substrates containing basic nitrogens (e.g., amino groups or pyridines) are able to coordinate the catalyst,^[^ ^26^ ^]^ decreasing the reaction rates of Suzuki‐Miyaura coupling,^[^ ^27^ ^]^ and negatively affecting the final yields. Clearly, the nucleophilicity of the amino group in tryptophane (or amino acid) derivatives is attenuated since the molecule is in its zwitterionic form.^[^ ^28^ ^]^

Pleasantly, using a series of boronic acids, we have reported, for the first time, a Pd‐catalyzed Suzuki‐Miyaura cross‐coupling reaction of brominated bisindole derivatives, including the natural product **1a**, protecting neither the indole nor the amino group (Scheme 1c). The same catalytic system can also be successfully applied to bromotryptamine derivatives. Moreover, the antileishmanial activity of the obtained arylated bisindoles has been evaluated.

## 2 Results and Discussion

Using the natural product **1a** as a model substrate, we started our investigation by applying similar conditions to those reported by Jin and coworkers.^[^ ^25^ ^]^ In particular, in the presence of 2.5 equivalents of the phenylboronic acid **2a**, 8 mol% of Pd(dppf)Cl_2_ as catalyst, and K_3_PO_4_∙H_2_O as the base in dioxane/H_2_O = 4:1 at 80°C, after 16 hours the desired diarylated 2,2‐bis(6‐phenyl‐1*H*‐indol‐3‐yl)ethan‐1‐amine compound **3aa** was achieved with 61% isolated yields (Table 1, entry 1). Concomitantly, in the absence of boronic acid **2a**, we performed some stability tests of compound **1a**, which seems to be stable at 80°C in the dioxane/H_2_O = 4:1 medium, since it was completely recovered by column chromatography (entry 2). However, adding the catalyst and the base (entry 3) or just the base (entry 4), only 63% of bisindole **1a** was found, suggesting that a partial decomposition of the molecule takes place. This effect seems more likely ascribable to the basic environment with respect to possible Pd‐catalyzed side reactions (compare entries 3 and 4). Changing the base to Cs_2_CO_3_, similar results to K_3_PO_4_∙H_2_O were obtained (entry 5), while with KOH (entry 6) or K_2_CO_3_ (entry 7), lower yields of **3aa** were achieved. Unfortunately, the amino group of **1a** is not sufficient for the reaction to proceed, indeed when no external base is present the reaction does not take place (entry 8). Screening other bases and other parameters, such as the reaction medium, the ratio of dioxane/H_2_O, the concentration of the species, and other Pd salts did not lead to any improvement (see Supporting Information). Surprisingly, progressively halving the reaction time to 1 hour, a complete **1a** conversion and a slightly higher **3aa** yield were obtained (entry 9). To speed up the reaction, and therefore reduce decomposition, various Pd catalysts were tested. The reaction was stopped when complete starting material conversion was reached (monitored by TLC) and, in any case, after 1 hour (entries 10–15). We observed that the reaction mixture usually turns dark brown when **1a** is completely consumed. Despite the utilization of other bidentate (entries 10–11) or monodentate (entries 12–15) phosphine ligands, Pd(dppf)Cl_2_ was confirmed as the best catalyst. Interestingly, the monoarylated product **4aa** was detected, being the main product utilizing Pd(Xphos)_2_Cl_2_ (entry 12). Finally, switching the base to Cs_2_CO_3_, a 79% isolated yield was reached in 1 hour in the presence of the best catalyst Pd(dppf)Cl_2_ (entry 16), since a further catalyst screening with this base was unsuccessful (entries 17–20). Doubling the amount of boronic acid or the catalyst loading essentially did not result in any changes. Unfortunately, a decrease in the **3aa** yield was observed lowering the amount of the base or the catalyst loading (see Supporting Information).

**Table 1:** Screening of the reaction conditions.^[^ ^a^ ^]^

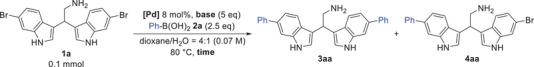						
Entry	[Pd]	Base	Time [h]	Conversion 1a [%]^[^ ^b^ ^]^	Yield 3aa [%]^[^ ^b^ ^]^	Yield 4aa [%]^[^ ^b^ ^]^
1	Pd(dppf)Cl_2_	K_3_PO_4_·H_2_O	16	100	61^[^ ^c^ ^]^	< 2
2^[^ ^d^ ^]^	‐	‐	16	< 2	0	0
3^[^ ^d^ ^]^	Pd(dppf)Cl_2_	K_3_PO_4_·H_2_O	16	37	0	0
4^[^ ^d^ ^]^	‐	K_3_PO_4_·H_2_O	16	37	0	0
5	Pd(dppf)Cl_2_	Cs_2_CO_3_	16	98	60^[^ ^c^ ^]^	< 2
6	Pd(dppf)Cl_2_	KOH	16	n.d.	31^[^ ^c^ ^]^	n.d.
7	Pd(dppf)Cl_2_	K_2_CO_3_	16	98	16^[^ ^c^ ^]^	n.d.
8	Pd(dppf)Cl_2_	‐	16	15	0	0
9	Pd(dppf)Cl_2_	K_3_PO_4_·H_2_O	1	100	70 (69^[^ ^c^ ^]^)	< 2
10	Pd(dppe)Cl_2_	K_3_PO_4_·H_2_O	1	95	43	25
11	Pd(dppp)Cl_2_	K_3_PO_4_·H_2_O	1	93	38	16
12	Pd(Xphos)_2_Cl_2_	K_3_PO_4_·H_2_O	1	71	11	59
13	*t*BuXPhos Pd G1	K_3_PO_4_·H_2_O	1	90	7	0
14	Pd(PPh_3_)_2_Cl_2_	K_3_PO_4_·H_2_O	1	100	59	20
15	(PPh_3_)_4_Pd	K_3_PO_4_·H_2_O	1	85	18	38
16	Pd(dppf)Cl_2_	Cs_2_CO_3_	1	100	79 (79^[^ ^c^ ^]^)	< 2
17	Pd(Binap)Cl_2_	Cs_2_CO_3_	1	79	11	25
18	Pd(DPEPhos)Cl_2_	Cs_2_CO_3_	1	95	52	16
19	Pd(dppf)(OAc)_2_^[^ ^e^ ^]^	Cs_2_CO_3_	1	90	29	32
20	Pd(JhonPhos)_2_(OAc)_2_^[^ ^e^ ^]^	Cs_2_CO_3_	1	55	5	37

Abbreviation: n.d. = not determinable.

^[a]^ Reaction performed under N_2_ inert atmosphere using bisindole **1a** (0.1 mmol), boronic acid **2a** (0.25 mmol), the indicated Pd source (8 mol% of catalyst loading), the indicated base (0.5 mmol), in dioxane/H_2_O = 4:1 (0.07 M) as the reaction medium at 80°C for the indicated time.

^[b]^ Determined by ^1^H NMR analysis of the reaction crude using 1,3,5‐trimethoxybenzene as standard.

^[c]^ Isolated yield by means of chromatographic column.

^[d]^ No boronic acid was utilized.

^[e]^ The catalyst was formed in situ by mixing Pd(OAc)_2_ and dppf or JhonPhos ligand (ratio, Pd:ligand = 1:2).

With the optimized conditions in hand (Table 1, entry 16), we investigated the scope of this late‐stage Suzuki‐Miyaura functionalization reaction (Table 2).

**Table 2:** Scope of the Pd‐catalyzed Suzuki‐Miyaura coupling of unprotected bisindoles.

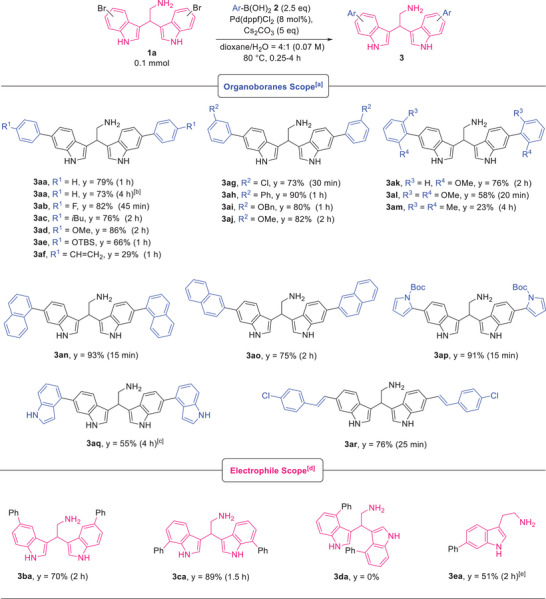

^[a]^ Reaction performed under N_2_ inert atmosphere using bisindole **1a** (0.1 mmol), boronic acid **2** (0.25 mmol), 8 mol% of Pd(dppf)Cl_2_, 0.5 mmol of Cs_2_CO_3_, in dioxane/H_2_O = 4:1 (0.07 M) at 80°C for the indicated time. Isolated yields are reported.

^[b]^ In place of the boronic acid, the phenylboronic acid pinacol ester was utilized.

^[c]^ In place of the boronic acid, 4‐(4,4,5,5‐tetramethyl‐1,3,2‐dioxaborolan‐2‐yl)‐1*H*‐indole was utilized.

^[d]^ Reaction performed under N_2_ inert atmosphere using 4‐, 5‐, and 7‐bromobisindoles **1** or tryptamine (0.1 mmol), phenylboronic acid **2a** (0.25 mmol), 8 mol% of Pd(dppf)Cl_2_, 0.5 mmol of Cs_2_CO_3_, in dioxane/H_2_O = 4:1 (0.07 M) at 80°C for the indicated time. Isolated yields are reported.

^[e]^ 1.25 equivalents of boronic acid **2a** (0.125 mmol), 4 mol% of catalyst loading, and 2.5 equivalents of base (0.25 mmol) were utilized.

To start with, we tested our reaction conditions using phenylboronic acid pinacol ester in place of the corresponding boronic acid and, gratifyingly, 73% isolated yield of **3aa** was reached after 4 hours, indicating that the desired product is accessible also using boronic esters, although longer reaction times are necessary. After, a series of boronic acids^[^ ^29^ ^]^ were utilized and the reactions were stopped when a complete starting material conversion was achieved (monitored by TLC) and, in case, for a maximum of 4 hours, in order to minimize the decomposition of the reagents/products. It should be noted that in all cases, only traces (less than 5%) of the monoarylated product **4** were observed. Using *para*‐substituted phenyl boronic acids, high yields were obtained in the presence of fluorine (**3ab**, 82%), isobutyl (**3ac**, y = 76%), or methoxy (**3ad**, y = 86%) substituents. Using the (4‐hydroxyphenyl)boronic acid, no reaction occurred while protecting the hydroxy group with the *tert*‐butyldimethylsilyl (TBS) afforded the desired product **3ae** in 66% isolated yields. Employing the (4‐vinylphenyl)boronic acid **2f** only 29% of **3af** was obtained, although it should be noted that under these developed conditions, the Heck product of the reaction was not detected, and the result could be mainly ascribable to side reactions only involving the boronic acid, such as polymerizations or protodeboronation. When *meta*‐substituted phenyl boronic acids were employed, good yields were still obtained, ranging from 73% to 90% ([3‐chlorophenyl]boronic acid [**3ag**] and [1,1′‐biphenyl]‐3‐ylboronic acid [**3ah**], respectively). Methoxy or benzyloxy *meta*‐substituents essentially both led to the same result with yields around 80% (**3ai** and **3j**). When a methoxy group was present in the *ortho* position, a good yield was achieved (**3ak**, 76%), and remained acceptable even when two *o*‐OMe were present (**3al**, 58%), despite the expected steric interactions. Using the (2,6‐dimethylphenyl)boronic acid **2m**, a less satisfying result was obtained (**3am**, 23%), probably due to a more pronounced steric effect of the methyl group with respect to the methoxy group^[^ ^30^ ^]^ (compare **3al** and **3am**). Notably, with this encumbered substrate, a 27% of the monoarylated product **4am** was also isolated (see Supporting Information for the characterization). The coupling between the naphthalen‐1‐ylboronic acid **2n** and **1a** gives an excellent yield of product **3an** in a very short time (93% in 15 minutes) and good results are also obtainable with naphthalen‐2‐ylboronic acid (**3ao**, 75%). Then, we tested heteroarylboranes bearing a pyrrole or an indole scaffold, successfully affording the respective compounds **3ap** and **3aq** in excellent and modest yields, respectively. Notably, our C(sp^2^)‐C(sp^2^) Suzuki‐Miyaura coupling can also be applied to vinyl boronic acids, as bisindole **3ar** is obtained in 76% isolated yields.

From the results reported above, longer reaction times seem to be generally required when electron donating groups are present, although a clear trend of reactivity based on the substituents on the boronic acid does not emerge, and generally satisfying yields are obtained regardless of the organoboranes utilized. Unfortunately, alkylboronic acids, such as butylboronic acid, are not reactive. Then, we tested other bromobisindoles^[^ ^29^ ^]^ in the presence of the phenylboronic acid **2a**. While using the 5‐bromobisindole **1b** and 7‐bromobisindole **1c**, good and high yields are respectively obtained (compare **3ba** and **3ca**), but the 4‐bromobisindole **1d** resulted to be completely unreactive, probably due to the presence of the bisindole ethylenimine group that sterically prevents the coupling. This result seems to further exclude a possible interaction of the catalyst with the amino group under our developed conditions, since, acting the NH_2_ as a directing group, a facile functionalization of the indole 4‐position was instead expected. Finally, for the first time, the Suzuki‐Miyaura coupling was applied to a completely NH‐indole and NH_2_‐unprotected tryptamine. Indeed, the 6‐bromotryptamine was gratifyingly converted into the desired product **3ea** with 51% isolated yields.

**Scheme 2:**
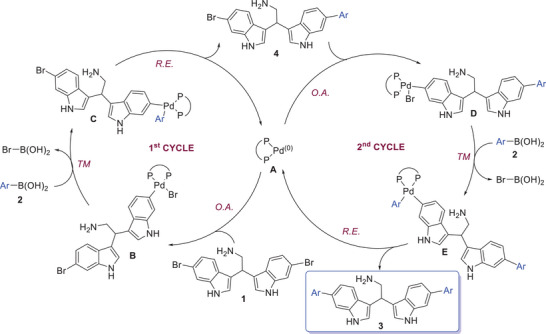
Proposed catalytic cycle. O.A. = oxidative addition; R.E. = reductive elimination; TM = transmetalation.

A plausible catalytic cycle for the reaction, based on our results and literature data,^[^ ^31^ ^]^ is proposed in Scheme 2.

Initially, an oxidative addition on one of the C‐Br bonds of the electrophile bisindole **1a** takes place, affording the intermediate **B**, followed by a transmetalation with the boronic acid **2**. It is known that the transmetalation step is assisted by the base since two possible pathways can occur, involving the formation of either an organoboronate ArB(OH)_3_^−^ species or a Pd‐hydride complex.^[^ ^31^ ^]^ In both cases, intermediate **C** is eventually formed, which undergoes reductive elimination, leading to the Pd(0) complex **A** and the monoarylated bisindole **4**. The latter, re‐entering the cycle and following the already described steps, finally affords the desired diarylated bisindole **3**. Based on the results obtained in Table 2, both the cycles operate concomitantly.

Finally, eighteen compounds were first screened for efficacy against *L. infantum* MHOM/TN/80/IPT1 promastigotes at a single dose of 20 µM for 72 hours. Almost all compounds showed a potent activity against the parasite (inhibition ≥ 92.6%) (Table 3), except for compounds **3ai**, **3ah**, **3ac,** and **3al** that were poorly active (inhibition ≤ 51.4%). Contextually, the cytotoxicity of the molecules was investigated on THP‐1 cells at 100, 20, and 4 µM for 72 hours to roughly determine the CC_50_ interval, that was estimated between 4 and 20 µM for all compounds, with the exception of **3ah**, **3ac,** and **3al** (20 µM < CC_50_ < 100 µM), which had previously shown poor activity on the parasites.

**Table 3:** Summary of bisindole derivatives activity on *L. infantum* promastigotes and THP‐1 cells and corresponding SIs.^[^ ^a^ ^]^ IC_50_ and CC_50_ values are reported as mean and 95% CI, from at least two independent experiments. Each experimental condition was conducted at least in duplicate, and miltefosine (Milt) was used as positive control.

	Inhibition of *L. infantum* at 20 µM (%)	Cytotoxicity on THP‐1 (100, 20, 4 µM) (%)	*L. infantum* IC_50_ (µM) (95% CI)	THP‐1 CC_50_ (µM) (95% CI)	SI^[^ ^a^ ^]^
**3af**	98.7	78, 97, −14	**1.1** (0.9–1.3)	24.0^[^ ^b^ ^]^	**21.8**
**3ca**	98.0	89, 98, −19	**1.7** (1.5–1.9)	18.1^[^ ^b^ ^]^	10.7
**3an**	97.8	79, 95, −15			
**3ab**	97.1	85, 97, −33			
**3aq**	96.9	89, 73, −16	2.8 (2.5–3.1)	14.6 (12.2–17.2)	5.2
**3ba**	96.8	91, 98, −22			
**3aj**	96.5	85, 97, 2			
**3ak**	96.5	86, 97, −4			
**3aa**	96.2	87, 97, −18			
**3ad**	95.1	86, 96, −12			
**3ao**	95.1	76, 94, −13			
**3ar**	95.1	66, 95, −32			
**3ag**	93.6	84, 97, −7			
**3ap**	92.6	85, 97, 22			
**3al**	51.4	84, 47, −5			
**3ac**	36.6	88, 1, −17			
**3ah**	25.3	77, 32, −20			
**3ai**	19.7	72, 90, 1			
Milt	98.5	99, 6, −35	3.7 (3.3–4.1)	36.6 (32.8–40.6)	9.8

^[a]^ Selectivity index = CC_50_/IC_50_.

^[b]^ CI was not calculable.

From this preliminary phenotype screening, it appeared that the efficacy of the arylbisindole **3** on *L. infantum* promastigotes and the toxicity on THP‐1 cells did not depend on the aryl substituent or on its position.

**Figure 2:**
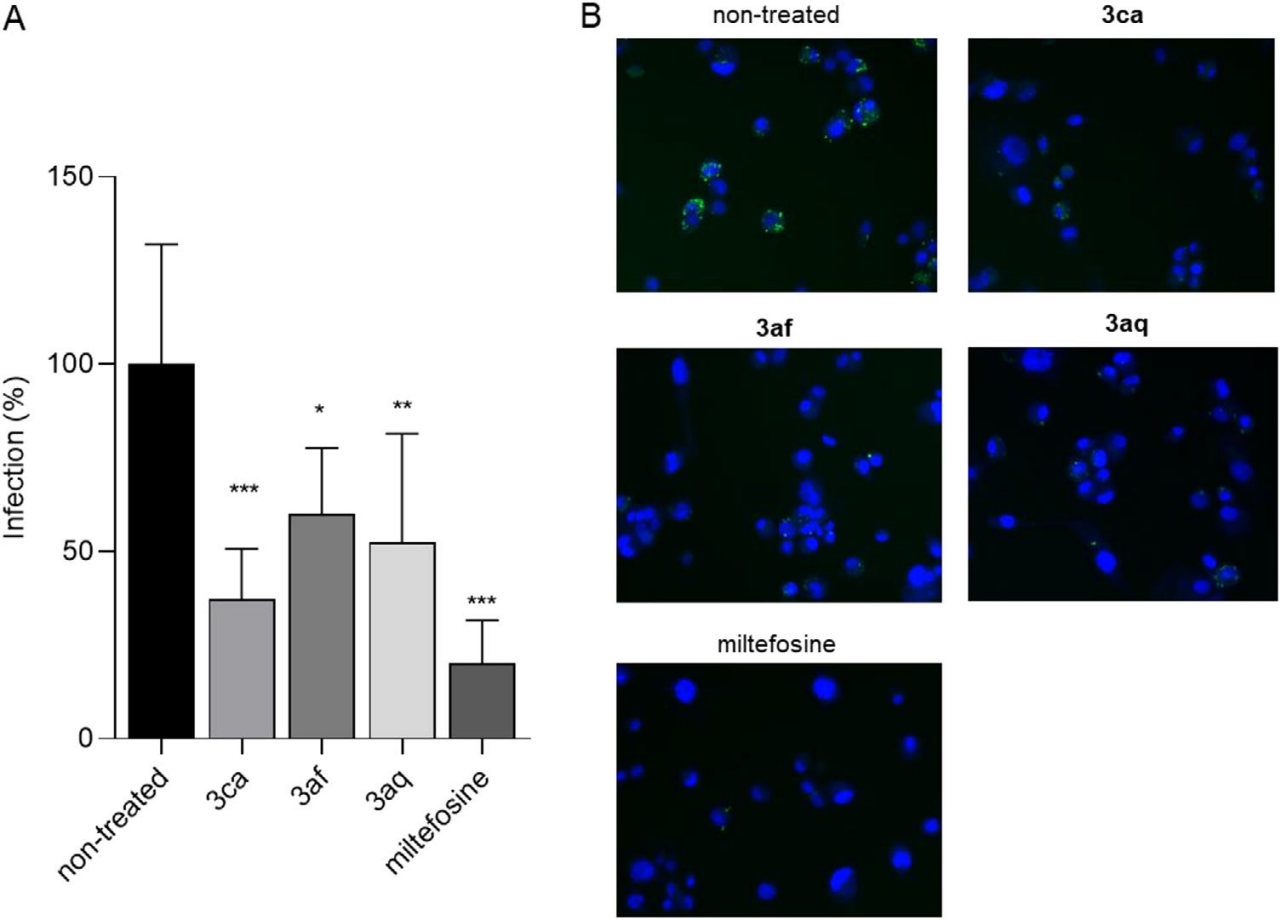
Effect of **3ca**, **3af**, and **3aq** on intracellular *L. infantum* amastigotes. Differentiated THP‐1 cells were infected for 24 hours at 37°C; then, the different compounds were added at 2 µM, and the efficacy on intracellular amastigotes was calculated after 72 hours of treatment. Miltefosine was used as a positive control. (A) Percentages of infection are presented as mean ± SD. Ordinary one‐way ANOVA with Dunnett's multiple comparisons test. *p ≤ 0.05, **p ≤ 0.01 *** *p* ≤ 0.001. (B) Representative merged images showing THP‐1 cells stained with Hoechst dye (blue) and *Leishmania* amastigotes stained with CFSE (green).

Based on these initial results, compounds **3af**, **3ca,** and **3aq** were selected for further tests to determine the exact IC_50_ and CC_50_ values. The bisindoles **3af** (bearing a 4‐vinylphenyl moiety) and **3ca** (7‐phenylbisindole) were chosen for their potent activity on the parasites (inhibition > 98%) while the tetraindole **3aq** was selected due to possible lower toxicity on THP‐1 cells at 20 µM (72.9%, Table 3). These selected compounds were further tested on *L. infantum* promastigotes with scalar dilutions 2:3 (from 20 to 1.17 µM) and on THP‐1 cells with scalar dilutions 1:2 (from 100 to 3.12 µM). Concerning the activity on promastigotes, molecules **3af**, **3ca,** and **3aq** showed IC_50_ values lower than miltefosine (i.e., 1.1, 1.7 and 2.8 µM, respectively) (Table 3), while regarding cytotoxicity on THP‐1 cells, they showed CC_50_ values between 14.6 and 24 µM (Table 3), comparable to the starting bromobisindole **1a**.^[^ ^15^ ^]^ Notably, in the CC_50_ calculation on THP‐1 cells, the 95% confidence interval (CI) was not determined for **3af** and **3ca** due to the steeper slope in dose‐response curves (Figure S9). Although **3af**, **3ca,** and **3aq** appeared relatively toxic to human cells, their SIs were 21.8, 10.7 and 5.2, respectively. It is noteworthy that the SI of miltefosine (reference antileishmanial drug) was 9.8. To further explore the potential of these three promising compounds, their activity was assessed against amastigotes in an in vitro infection model. All compounds showed a significant reduction in the infection at 2 µM (Figure 2), confirming their efficacy on the corresponding protozoan form inside the macrophage‐like human cells. Therefore, despite a slightly higher cytotoxicity than miltefosine (as also shown in Figure S10, Supporting Information), **3af**, **3ca,** and **3aq** were the most promising compounds of our new library, showing an inhibitory activity on *L. infantum* promastigotes and intramacrophage amastigotes.

## 3 Conclusion

In this study, we developed a robust, rapid, and efficient method for the Suzuki‐Miyaura coupling of NH₂‐unprotected bromobisindole ethanamines, allowing the late‐stage functionalization of these scaffolds with a variety of boronic acids. High yields (up to 93%) of diarylated products were obtained using Pd(dppf)Cl₂ as the catalyst, Cs₂CO₃ as the base, and dioxane/H_2_O = 4:1 as the reaction medium. A broad scope and excellent functional group tolerance are demonstrated, and even sterically hindered arylboronic acids or vinylboronic acids can be employed. Remarkably, both the NH_2_ and the indolic NH of the starting bromobisindole ethanamines are unprotected, suggesting this methodology can be applied to late‐stage diversification of unprotected halobisindoles and, more generally, to unprotected haloindole derivatives. Indeed, to the best of our knowledge, using our procedure, a bromotryptamine (6‐Br) was directly arylated for the first time achieving the corresponding product with good yield.

Eighteen compounds from our synthesized library were also tested against *L. infantum* promastigotes. Among the tested substrates, **3af** (bearing a 4‐vinylphenyl moiety) emerged as the most promising candidate, showing a SI of 21.8, significantly higher than the reference drug miltefosine (9.8), with an IC_50_ = 1.1 µM. Bisindoles **3ca** and **3aq** also demonstrated high activity, although with a slightly lower SI. A significant activity of **3af**, **3ca,** and **3aq** was also reported against intramacrophage amastigotes in an in vitro infection model. Using our developed synthesis, future studies will focus on improving the pharmacokinetic profiles of these compounds, in order to enhance their potential as therapeutic agents against leishmaniasis.

## Conflict of Interests

The authors declare no conflict of interest.

## Supporting information



Supporting Information

## Data Availability

The data that support the findings of this study are available in the Supporting Information of this article.
