# Supplementary material for: Efficient and Rapid Arylation of NH₂‐Unprotected Bromobisindole Ethanamines via Suzuki‐Miyaura Coupling: Generating New Leads Against Leishmania
Source: Chemistry. 2025 May 19;31(43):e202500637. doi: 10.1002/chem.202500637 (PMC12319374; doi:10.1002/chem.202500637)
Supplement: Supplementary file 1 — Supporting Information [file CHEM-31-e202500637-s001.docx]

**Efficient and Rapid Arylation of NH₂-Unprotected Bromobisindole Ethanamines *via* Suzuki-Miyaura Coupling: Generating New Leads against *Leishmania***

Alessandro Buono,^[a]^ Aurora Diotallevi,^[b]^ Sara Maestrini,^[b]^ Michele Verboni,^[a]^ Paula Kiuru,^[c]^ Luca Galluzzi,^[b]^ Andrea Duranti,^[a]^ Diego Olivieri,^[a]^ Simone Lucarini*^[a]^

[a] A. Buono, Dr. M. Verboni, Dr. A. Duranti, Dr. D. Olivieri, Dr. S. Lucarini
Department of Biomolecular Sciences, Section of Chemistry and Pharmaceutical Technologies
University of Urbino Carlo Bo
Campus Scientifico E. Mattei, via ca’ le suore 2, 61029 Urbino, PU, Italy
E-mail: [simone.lucarini@uniurb.it](mailto:simone.lucarini@uniurb.it)

[b] Dr. A. Diotallevi, S. Maestrini, Dr. L. Galluzzi
Department of Biomolecular Sciences, Section of Biochemistry and Biotechnology
University of Urbino Carlo Bo
Via Arco d'Augusto 2, 61032 Fano, PU, Italy

[c] P. Kiuru

Drug Research Program, Division of Pharmaceutical Chemistry and Technology, Faculty of Pharmacy

University of Helsinki

Viikinkaari 5E, P.O. Box 56, 00014 Helsinki, Finland

**Supporting Information**

**Contents:**

| 1. Material and methods |  | S2 |
| --- | --- | --- |
| 1. List of the utilized bromo(bis)indoles **1** and boronic acids **2** |  | S5 |
| 1. Complete Optimization Reactions for the Suzuki-Miyaura Coupling of Unprotected Bromobisindole |  | S6 |
| 1. ^1^H NMR and ESI-MS characterization of 2-(6-bromo-1*H*-indol-3-yl)-2-(6-phenyl-1*H*-indol-3-yl)ethan-1-amine **4aa** |  | S8 |
| 1. Synthesis of 2,2-bis(7-bromo-1*H*-indol-3-yl)ethan-1-amine **1c** and 2,2-bis(4-bromo-1*H*-indol-3-yl)ethan-1-amine **1d** |  | S11 |
| 1. Experimental data for compounds **3** and **4** |  | S14 |
| 1. ^1^H NMR and ^13^C NMR Spectra |  | S25 |
| 1. Dose-response curves of **3af**; **3ca** and **3aq** on *L. infantum* promastigotes |  | S47 |
| 1. Dose-response curves of **3af**; **3ca** and **3aq** on THP-1 cells |  | S48 |
| 1. Representative phase-contrast images of THP-1 cells infected and treated with compounds **3af**, **3ca** and **3aq**, and miltefosine |  | S49 |
| 1. References |  | S50 |

1. **Material and methods**

**Chemistry**

All reactions were prepared under nitrogen atmosphere with dry solvents under anhydrous conditions, by using Schlenk technique. Reactions were monitored by ^1^H NMR taking a direct sample of the crude mixture. ^1^H NMR and ^13^C NMR were recorded on a Bruker Avance 400 spectrometer (^1^H: 400 MHz, ^13^C: 101 MHz), or on a Bruker Avance Neo 600 spectrometer (^1^H: 600 MHz, ^13^C: 151 MHz) using DMSO-*d_6_* or CDCl_3_ as solvent and 1,3,5-trimethoxybenzene as standard. Chemical shifts are reported in the *δ* scale relative to residual CHCl_3_ (s, 7.26 ppm) or DMSO (p, 2.50 ppm) for ^1^H NMR and to the central line of CDCl_3_ (77.16 ppm) or DMSO-*d_6_* (39.52 ppm) for ^13^C NMR. ^13^C NMR were recorded with ^1^H broadband decoupling. The following abbreviations were used to explain the multiplicities: br = broad, s = singlet, d = doublet, t = triplet, q = quartet, hept = heptet, dd = doublet of doublets, ddd = doublet of doublets of doublets, m = multiplet. Coupling constants (*J*) are reported in Hertz (Hz). ESI-MS spectra were recorded on Waters Micromass ZQ 4000, using electrospray ionisation techniques, with samples dissolved in MeOH. HRMS spectra were performed by slow direct infusion (5 μL/min) of ≈ 0.1 μg/mL solution (methanol), using Orbitrap Exploris 240 mass spectrometer. Only molecular ions [M + H]^+^ are given. For **Int-1c** and **Int-1d** molecular ions [M + Na]^+^ are given.

1,4-dioxane was dried over molecular sieves (Alfa Aesar, 4 Å, 1–2 mm, beads). Both 1,4-dioxane and water were degassed (N_2_ bubbling for 30 min).

Pure compounds **3** were isolated through flash column chromatography on silica gel 60 (40-60 μm, 230-400 mesh). Bromo-bisindole derivatives **1a** and **1b** have been synthesized as already described in the literature [1]. 6-bromotryptamine was synthesized as already described in the literature [2]. The new bromo-bisindoles **1c** and **1d** have been realized following a literature procedure [1], as reported in the Supporting Information. Boronic acids **2a**, **2c**, **2f** and boronic ester **2q** were purchased from BLDpharm. Boronic acids **2b**, **2i**, **2j** were purchased from Alfa Aesar - Thermo Fisher. Boronic acids **2d** and **2e** were purchased from TCI. Boronic acids **2g**, **2h**, **2l**, **2m**, **2r**, **2k** were purchased from Merck Sigma-Aldrich. Boronic acids **2n**, **2o**, **2p** were purchased from Fluorochem. Boronic ester **2a’** has been synthesized as already described in the literature [3]. All other chemicals were purchased from Merck Sigma-Aldrich and used without further purification. All solid reagents were weighed in an analytical balance without excluding moisture and air.

**Parasite and cell cultures**

*L. infantum* MHOM/TN/80/IPT1 (WHO international reference strain) was purchased from ATCC (ATCC® 50134™) and was cultured in Evans's Modified Tobie Medium (EMTM) at 26–28 °C. To test the compounds, parasites were resuspended in RPMI-PY medium as described previously [4]. The human monocytic cell line THP-1 (ECACC 88081201) was cultured in RPMI-1640 medium supplemented with 10% heat-inactivated Fetal Bovine Serum (FBS), 100 μg/ml streptomycin, 100 U/l penicillin and maintained in a humidified incubator at 37 °C and 5% CO_2_. All the cell culture reagents were purchased from Sigma-Aldrich (St. Louis, MO, USA).

***L. infantum* promastigotes viability assay**

Late log/stationary phase L. *infantum* promastigotes were resuspended in RPMI-PY medium at a density of 2.5×10^6^ parasites/mL in 96-well plates (100μL/well). The anti-parasitic activity of the eighteen compounds of the library was initially investigated at a single dose of 20 μM for 72 h at 26 °C. Three selected compounds were tested with scalar dilutions 2:3 (from 20 to 1.17 μM) on promastigotes to determine the exact IC_50_ value. Not treated parasites were included as negative control. As positive control, the anti-leishmanial drug miltefosine (Sigma-Aldrich, Milan, Italy) was used. Each condition was carried out in duplicate. To evaluate the promastigotes viability, the CellTiter 96H Aqueous Non-Radioactive Cell Proliferation Assay (Promega), based on the ability of viable cells to convert a soluble tetrazolium salt [3-(4.5-dimethylthiazol-2-yl)-5-(3-carboxymethoxy-phenyl)-2-(4-sulfophenyl)-2H-tetrazolium. MTS] to a formazan product, was performed. To this end, 20 μL of MTS/PMS (phenazinemethosulfate, Sigma-Aldrich) was added to 100 μL of culture medium and incubated at 26°C until formazan production. Absorbance was recorded on a Microplate Reader (Infinite® F50 Plus, Tecan) at 492 nm. The IC_50_ values were calculated using nonlinear regression curves in GraphPad Prism 8.0 (GraphPad Software,Inc., San Diego, CA). The equation used for data fitting was Y= 100/(1 + 10∧((Log IC_50_ −X)*HillSlope)) (hillslope not constrained), where X is the log of concentration and Y is the normalized response.

**Evaluation of cytotoxicity on THP-1 cells**

The cytotoxicity of the compounds was assessed on THP-1 cells seeded at a density of 5 × 10^5^ cells per mL, 100μL/well in a 96-well plate and treated for 72 h with 20 ng/mL phorbol myristic acid (PMA) to induce differentiation into macrophages like cells. After cell adhesion to the plate, each molecule was tested at 4, 20 and 100 μM for 72 h at 37˚C.

Three selected compounds were further evaluated at different concentration, obtained from scalar dilutions 1:2 (from 100 µM to 3.12 μM) for 72 h at 37°C. The negative control (untreated THP-1 cells) and the positive control (miltefosine) were included in each experiment. Each condition was carried out in duplicate. To evaluate the compounds cytotoxicity, the CellTiter 96H Aqueous Non-Radioactive Cell Proliferation Assay (Promega), was used as described before. For each compound the Selectivity Index (SI) was calculated as the ratio between cytotoxicity in THP-1 (CC_50_, 72 h) and activity against L. *infantum* promastigotes (IC_50_, 72 h).

**Anti-amastigote Assay on infected THP-1 cells**

The activity of compounds **3af**, **3ca**, and **3aq** against intracellular amastigotes was evaluated in THP-1 infected cells. Briefly, THP-1 cells were seeded in a 12-well plate with a density of 3 × 10^5^ cells/well and, to induce differentiation into macrophage-like cells, were treated with 20 ng/mL phorbol myristic acid (PMA) for 48 h. After differentiation, cells were infected for 24 h with *L. infantum* MHOM/TN/80/IPT1 promastigotes stained with carboxyfluorescein succinimidyl ester (eBioscience™ CFSE, Invitrogen) as described in reference [5]**,** with a parasite-to-cell ratio of 10:1. Non-internalized promastigotes were then removed through medium replacement, and infected cells were treated with **3af**, **3ca**, and **3aq** or with the positive control miltefosine for 72 h at concentrations of 2, 10, and 20 μM. To monitor the infection, cells were washed, formaldehyde/methanol fixed, stained with Hoechst dye, and observed with a fluorescence microscope at 5X or 10X magnification. For each field, two images were acquired: one with excitation at 340-390 nm (for Hoechst dye) and the other with excitation at 460-495 nm (for CFSE). Automated cell counting of single-color images was performed with ImageJ software to monitor the rate of infection, which was determined by normalizing the amastigote count from each green (CFSE) image to the THP-1 cell count from the corresponding blue (Hoechst) image. At least 500 THP-1 cells were counted for each condition. The normalized infection rate for untreated infected cells was set as 100%.

**Statistical Analysis**

The evaluation of IC_50_ in promastigotes and CC_50_ in mammalian cells following the treatments was performed by nonlinear regression analysis and expressed as mean and 95% confidence interval. Statistical evaluation of treatment efficacy on amastigotes was performed using one-way ANOVA, followed by Dunnett's multiple comparisons test. All tests were performed using GraphPad Prism version 8 (GraphPad Software, Inc., La Jolla, CA).

**Typical Procedure for the Suzuki-Miyaura coupling of unprotected bromo-bisindole with boronic acids**

Compound **1** (0.1 mmol, 43.3 mg), Pd(dppf)Cl_2_ (8mol%, 5.9 mg), Cs_2_CO_3_ (0.5 mmol, 162.9 mg) and the select boronic acid or pinacol derivative **2** (0.25 mmol) were added in a dried Schlenk tube, equipped with a magnetic stirring bar. Degassed dioxane (1.15 mL) and degassed water (0.28 mL) were added, and the reaction was vigorously stirred at 80°C in an oil bath under balloon pressure of nitrogen, monitoring the progress of the reaction by TLC (silica gel; dichloromethane/methanol/ammonia, 90/10/1). The nitrogen was removed, and the reaction mixture was analyzed by ^1^H NMR to determine the conversion of the bis-indole. The crude was filtered on silica plug (dichloromethane/methanol/ammonia, 80:20:1) and the products were eventually obtained after column chromatography on silica gel. For the synthesis of compound **3ea**, 0.125 mmol of boronic acid **2a**, 4 mol% of Pd(dppf)Cl_2_, and 0.25 mmol of base were utilized.

1. **List of the utilized bromo(bis)indoles 1 and boronic acids 2**

**Figure S1 –** Complete list of the utilized Br-(bis)indoles **1**

**Figure S2 –** Complete list of the utilized boronic acids or boronic esters **2**

1. **Complete Optimization Reactions for the Suzuki-Miyaura Coupling of Unprotected Bromobisindole**

**Table S1 –** Screening of the solvent, the concentration and the ratio organic solvent : water

| Entry^[a]^ | Solvent^[b]^ | Solvent : H_2_O (v/v) | Concentration [M] | Yield **3aa** [%]^[c]^ |
| --- | --- | --- | --- | --- |
| 1 | 1,4-dioxane | 4 : 1 | 0.07 | 61 |
| 2 | THF | 4 : 1 | 0.07 | 45 |
| 3 | CH_3_CN | 4 : 1 | 0.07 | 18 |
| 4 | *i*PrOH | 4 : 1 | 0.07 | 40 |
| 5 | 1,4-dioxane | 4 : 1 | 0.14 | 57 |
| 6 | 1,4-dioxane | 3 : 1 | 0.07 | 35 |
| 7 | 1,4-dioxane | 5 : 1 | 0.07 | 24 |
| 8 | 1,4-dioxane | 4 : 1 | 0.035 | 31 |

[a] Reaction performed under N_2_ inert atmosphere using bisindole **1a** (0.1 mmol), boronic acid **2a** (0.25 mmol), Pd(dppf)Cl_2_ (8 mol% of catalyst loading), K_3_PO_4_∙H_2_O (0.5 mmol), in the indicated reaction medium at 80°C for 16 h. [b] Solvents dried over molecular sieves. [c] Isolated yields.

**Table S2 –** Screening of the bases/additives

| Entry^[a]^ | Base | Yield **3aa** [%]^[b]^ |
| --- | --- | --- |
| 1 | K_2_CO_3_ | 16 |
| 2 | Cs_2_CO_3_ | 60 |
| 3 | KOH | 31 |
| 4 | *n*Bu_4_NOH | 0 |
| 5 | CsF | < 2 |

[a] Reaction performed under N_2_ inert atmosphere using bisindole **1a** (0.1 mmol), boronic acid **2** a(0.25 mmol), 8 mol% of Pd(dppf)Cl_2_, the indicated base (0.5 mmol), in dioxane/H_2_O = 4 : 1 (0.07 M) at 80°C for 16 h. [b] Isolated yields.

**Table S3 –** Screening of various palladium sources and catalysts

| Entry^[a]^ | [Pd] | Base | Conversion **1a** [%]^[b]^ | Yield **3aa** [%]^[b]^ | Yield **4aa** [%]^[b]^ |
| --- | --- | --- | --- | --- | --- |
| 1^[c]^ | Pd(dppf)(OAc)_2_^[d]^ | K_3_PO_4_∙H_2_O | > 98 | 21^[e]^ | n.d. |
| 2^[c]^ | Pd(dppf)Cl_2_^[d]^ | K_3_PO_4_∙H_2_O | > 98 | 21^[e]^ | n.d. |
| 3 | Pd(dppf)Cl_2_ | K_3_PO_4_∙H_2_O | 100 | 70 (69^[e]^) | < 2 |
| 4 | Pd(dppe)Cl_2_ | K_3_PO_4_∙H_2_O | 95 | 43 | 25 |
| 5 | Pd(dppp)Cl_2_ | K_3_PO_4_∙H_2_O | 93 | 38 | 16 |
| 6 | Pd(Xphos)_2_Cl_2_ | K_3_PO_4_∙H_2_O | 71 | 11 | 59 |
| 7 | *t*BuXPhos Pd G1 | K_3_PO_4_∙H_2_O | 90 | 7 | 0 |
| 8 | Pd(PPh_3_)_2_Cl_2_ | K_3_PO_4_∙H_2_O | 100 | 59 | 20 |
| 9 | (PPh_3_)_4_Pd | K_3_PO_4_∙H_2_O | 85 | 18 | 38 |
| 10 | Pd(dppf)Cl_2_ | Cs_2_CO_3_ | 100 | 79 (79^[e]^) | < 2 |
| 11 | Pd(Binap)Cl_2_ | Cs_2_CO_3_ | 79 | 11 | 25 |
| 12 | Pd(DPEPhos)Cl_2_ | Cs_2_CO_3_ | 95 | 52 | 16 |
| 13 | Pd(dppf)(OAc)_2_^[f]^ | Cs_2_CO_3_ | 90 | 29 | 32 |
| 14 | Pd(JhonPhos)_2_(OAc)_2_^[f]^ | Cs_2_CO_3_ | 55 | 5 | 37 |

[a] Reaction performed under N_2_ inert atmosphere using bisindole **1a** (0.1 mmol), boronic acid **2a** (0.25 mmol), the indicated Pd source (8 mol% of catalyst loading), the indicated base (0.5 mmol), in dioxane/H_2_O = 4 : 1 (0.07 M) as the reaction medium at 80°C for 1 h. [b] Determined by ^1^H NMR analysis of the reaction crude using 1,3,5-trimethoxybenzene as standard. [c] Reaction time = 16 h. [d] The catalyst was formed by mixing Pd(OAc)_2_ or Pd(cod)Cl_2_ with dppf (ratio Pd:ligand = 1:1) [e] Isolated yield by means of chromatographic column. [f] The catalyst was formed *in-situ* by mixing Pd(OAc)_2_ and dppf or JhonPhos ligand (ratio Pd:ligand = 1:2). n.d. = not determinable.

**Table S4 –** Screening of the effect of the amount of boronic acid **2a**, catalyst loading and base amount on the reaction

| Entry^[a]^ | Catalyst Loading [mol%] | Cs_2_CO_3_ [equiv] | **2a** [equiv] | Conversion **1a** [%]^[b]^ | Yield **3aa** [%]^[b]^ | Yield **4aa** [%]^[b]^ |
| --- | --- | --- | --- | --- | --- | --- |
| 1 | 8 | 5 | 4 | 100 | 77 | 1 |
| 2 | 16 | 5 | 2.5 | 100 | 72 | 3 |
| 3 | 16 | 5 | 4 | 100 | 76 | 0 |
| 4 | 16 | 1 | 4 | n.d. | 16 | 24 |
| 5 | 4 | 5 | 2.5 | >98 | 65 | 11 |

[a] Reaction performed under N_2_ inert atmosphere using bisindole **1a** (0.1 mmol) and boronic acid **2a**, Pd(dppf)Cl_2_ and Cs_2_CO_3_ in the indicated amount, in dioxane/H_2_O = 4 : 1 (0.07 M) as the reaction medium at 80°C for 1 h. [b] Determined by ^1^H NMR analysis of the reaction crude using 1,3,5-trimethoxybenzene as standard.

1. **^1^H NMR and ESI-MS characterization of 2-(6-bromo-1*H*-indol-3-yl)-2-(6-phenyl-1*H*-indol-3-yl)ethan-1-amine 4aa**

Compound **4aa** was isolated from entry 3 of Table S2 in a mixture with compound **1a** and compound **3aa** using silica gel column chromatography (dichloromethane / methanol / ammonia, 99 / 1 / 1). ^1^H NMR spectrum of the mixture (**4aa**:**1a**:**3aa** = 72:17:12) and ESI(+)-MS spectrum, confirming the formation of **4aa**, are reported below.

**4aa:** ^1^H NMR (400 MHz, DMSO-*d*_6_): *δ* = 10.98 (br s, 1H, NH_ind_), 10.93 (br s, 1H, NH_ind_), 7.62-7.59 (m, 2H, Ar-H), 7.54 (d, *J* = 1.5 Hz, 1H, Ar-H), 7.52 (d, *J* = 8.5 Hz, 1H, Ar-H), 7.50 (d, *J* = 2.0 Hz, 1H, Ar-H), 7.44-7.40 (m, 3H, Ar-H), 7.31-7.26 (m, 3H, Ar-H), 7.18 (dd, *J_1_* = 1.5 Hz, *J_2_* = 8.5 Hz, 1H, Ar-H), 7.00 (dd, *J_1_* = 2.0 Hz, *J_2_* = 8.5 Hz, Ar-H), 4.39 (t, *J* = 7.0 Hz, 1H, C*H*CH_2_NH_2_), 3.27 (d, *J* = 7.0 Hz, 1H, CHC*H_2_*NH_2_) ppm. ESI-MS: m/z = 430 [M+H]^+^.


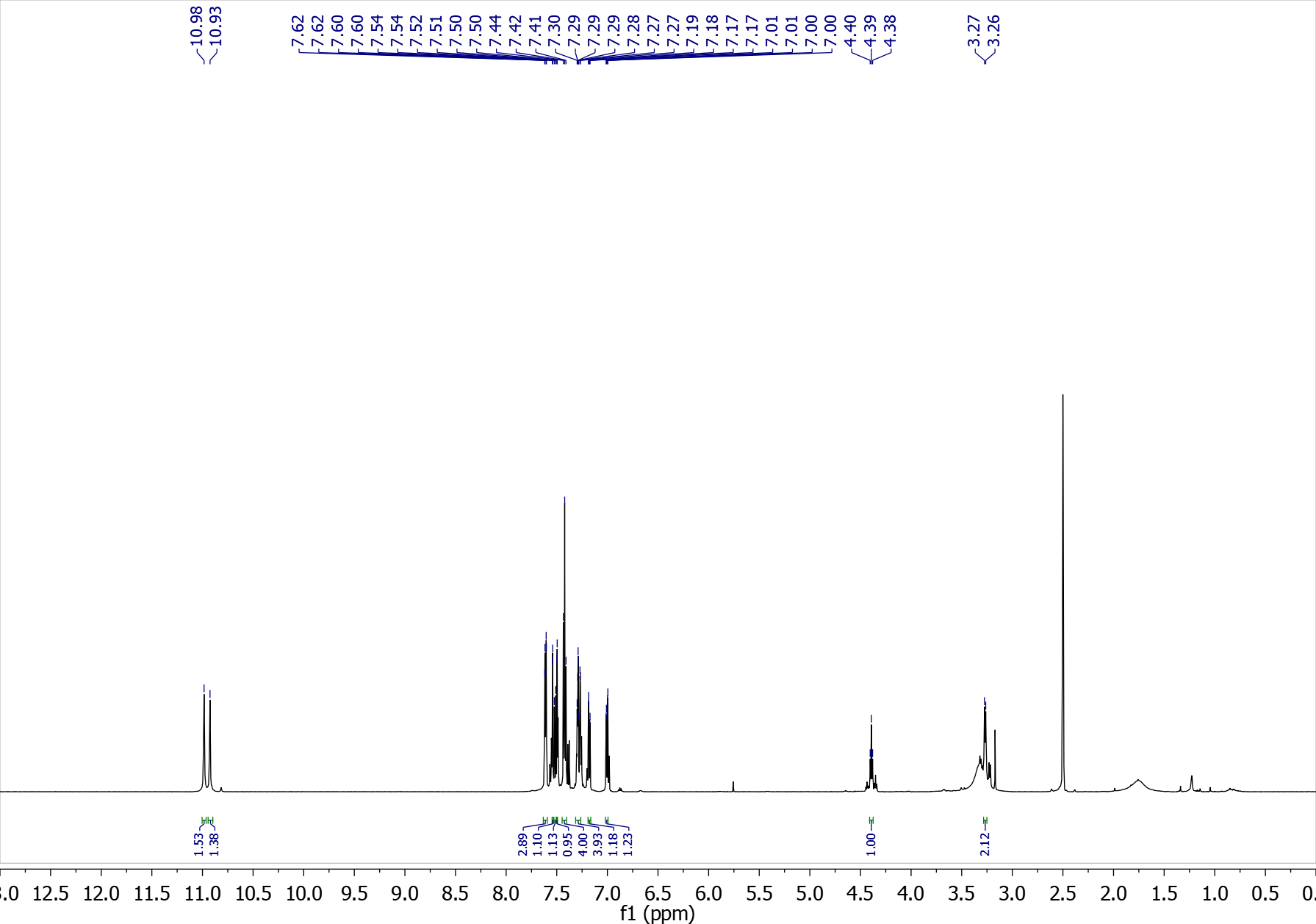


**Figure S3** –^1^H NMR of the isolated mixture.


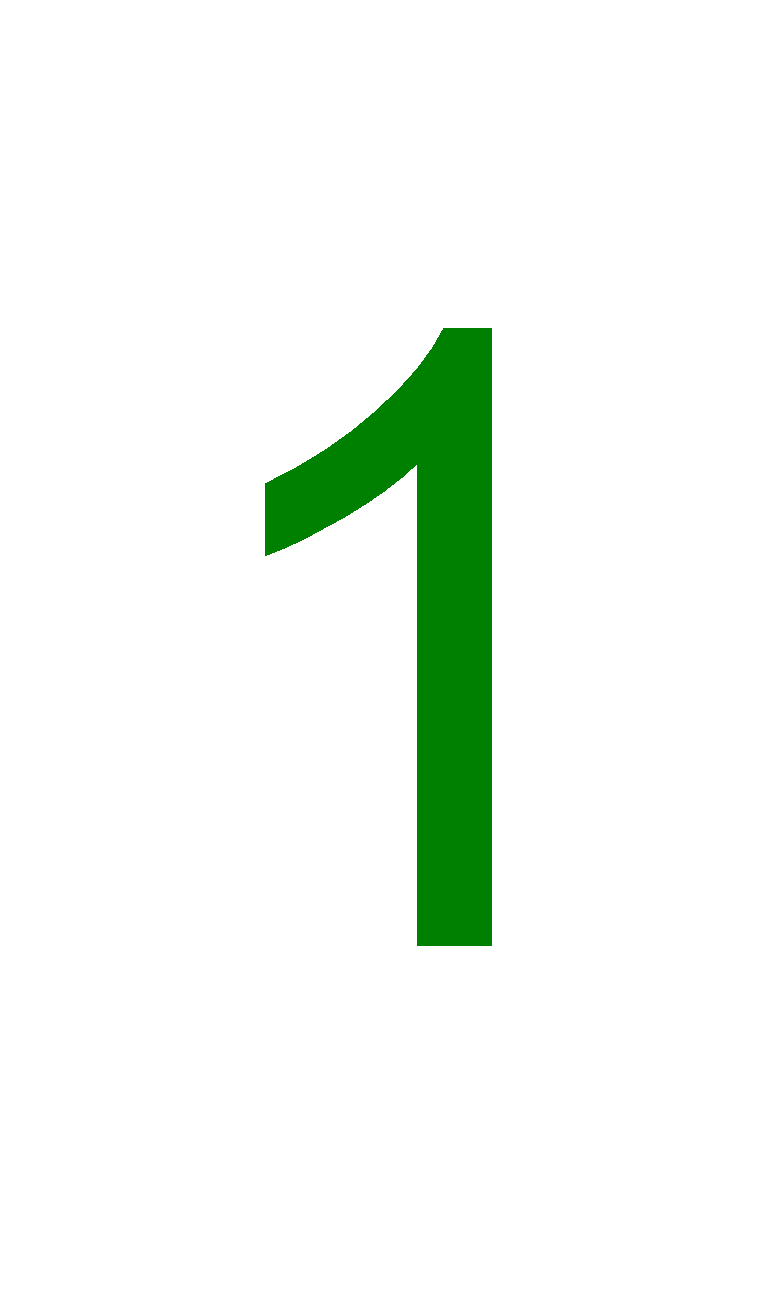

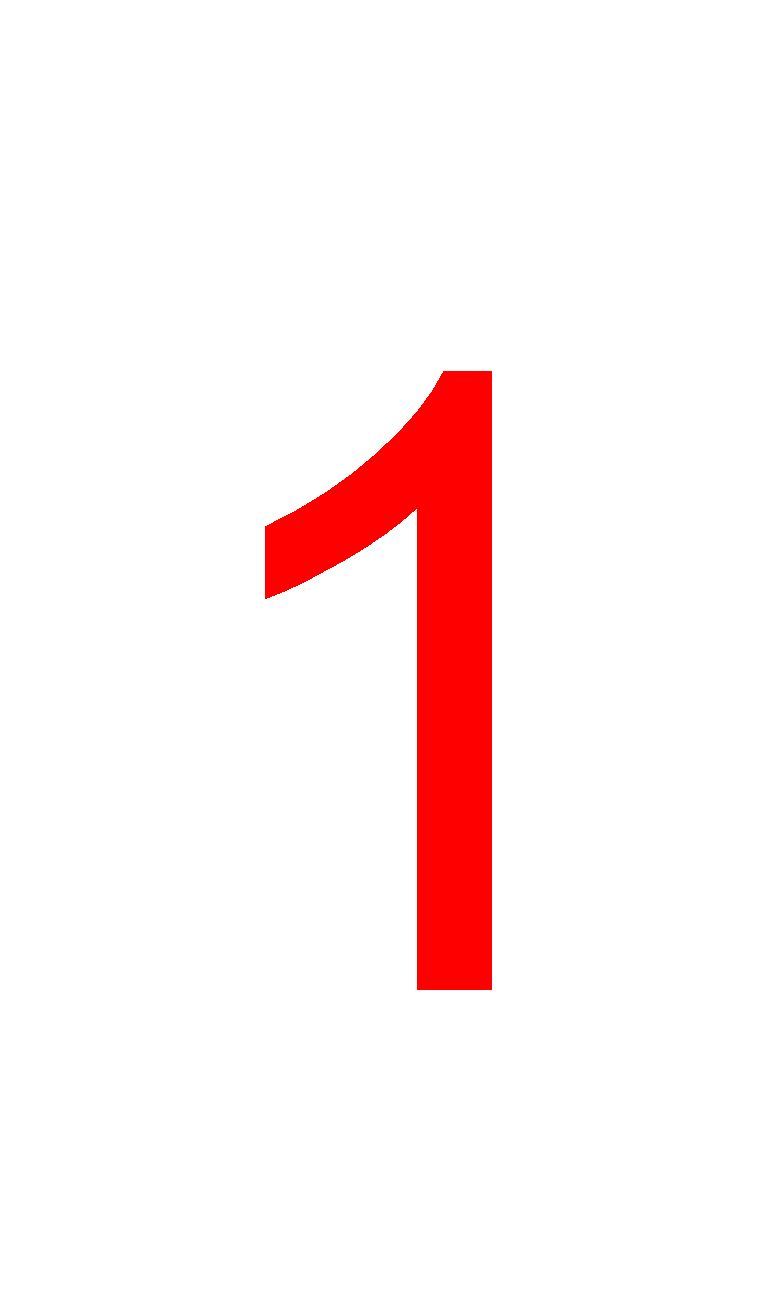


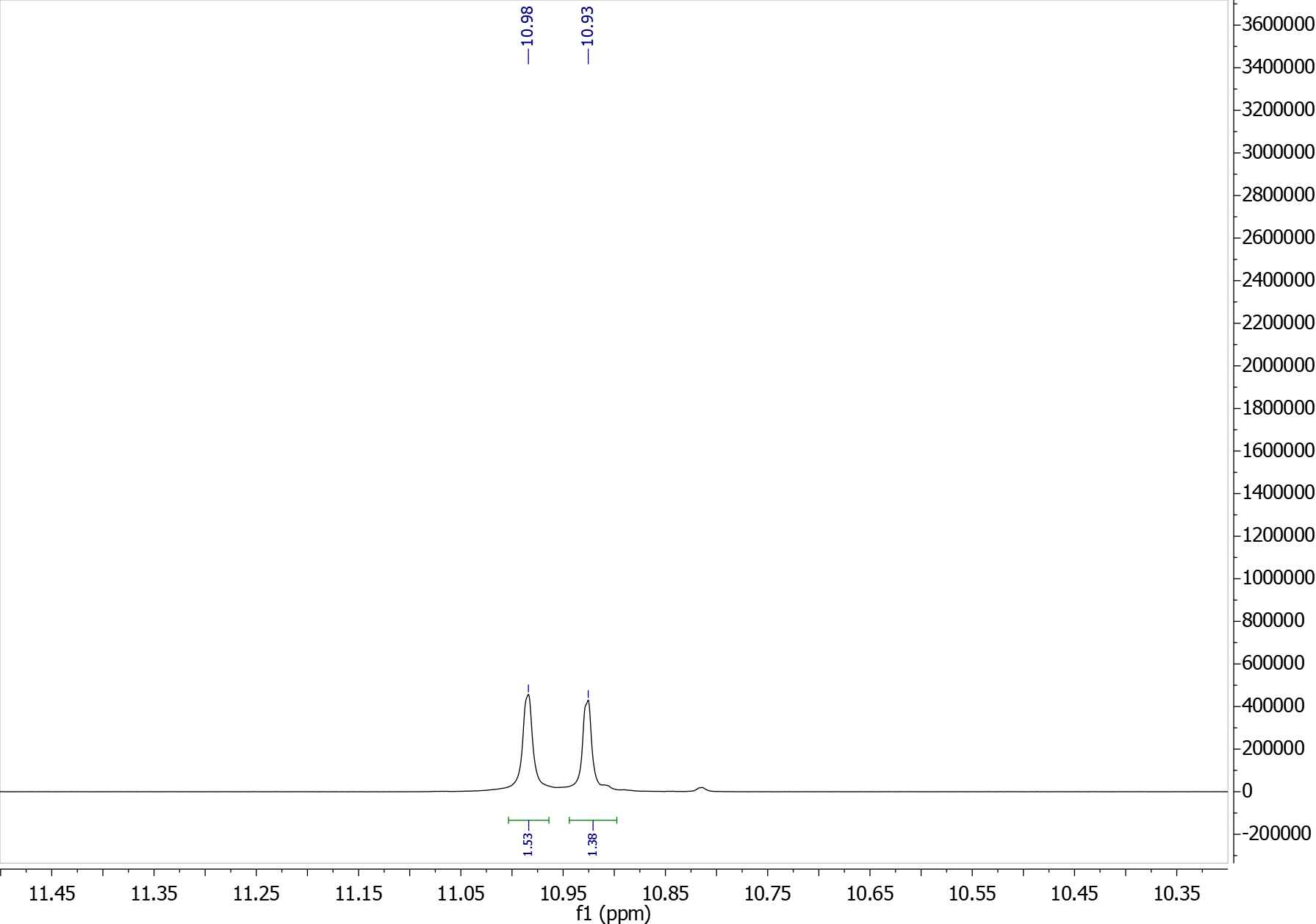

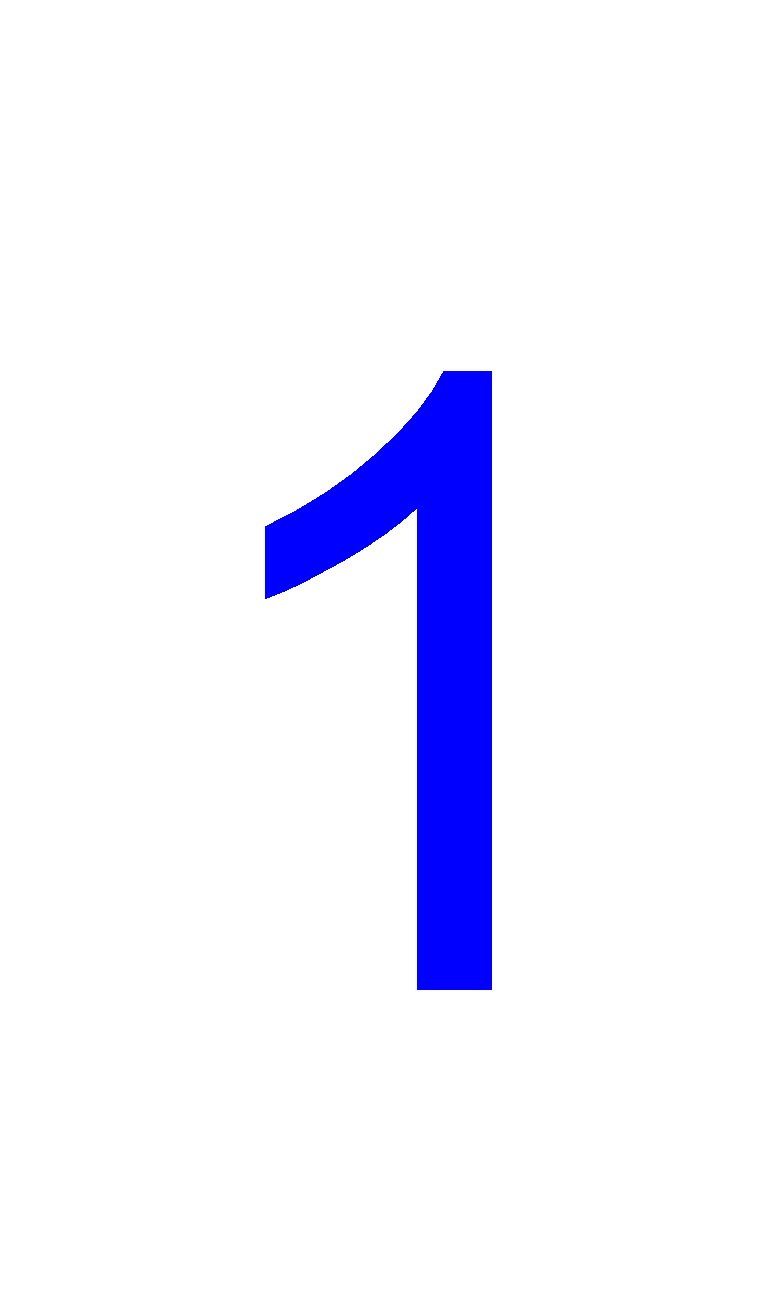

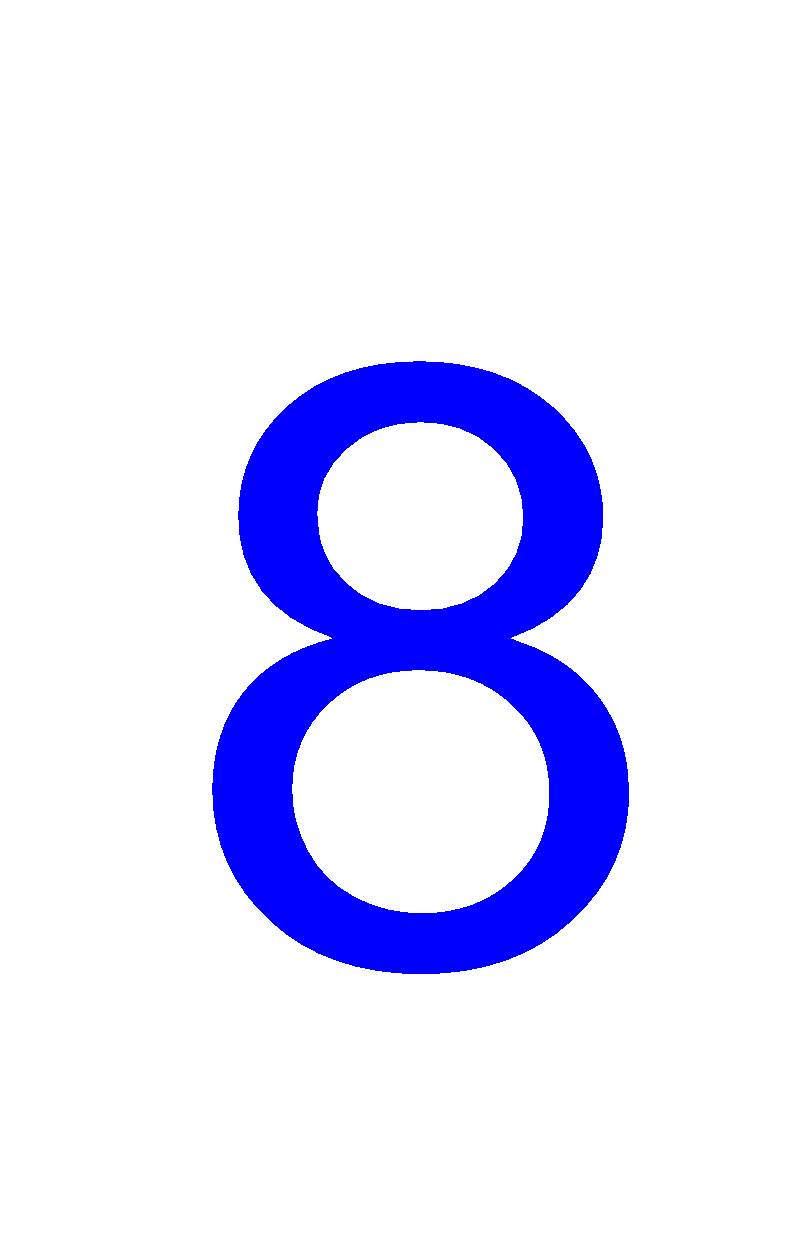


**Figure S4** –^1^H NMR of the isolated mixture. Zoom from 11.50 ppm to 10.40 ppm.


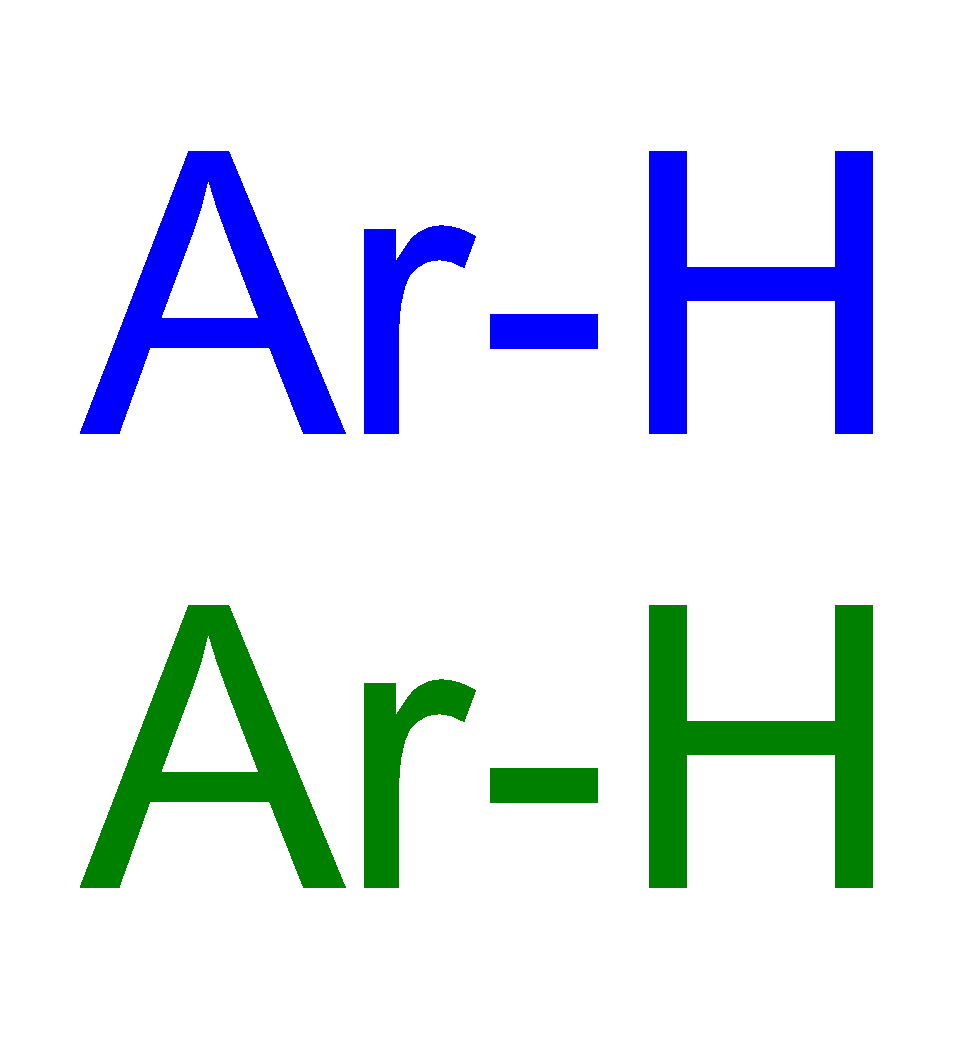


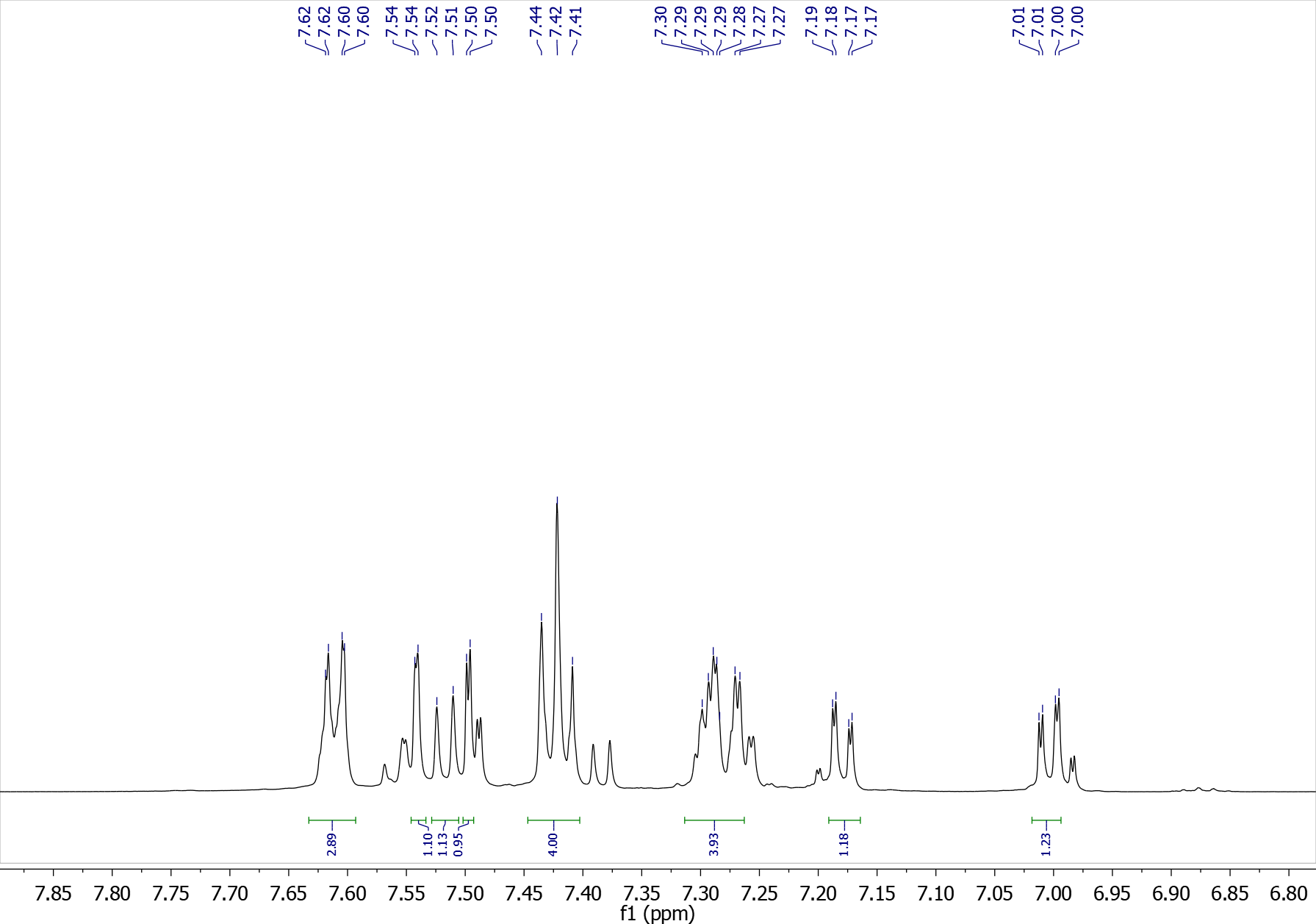

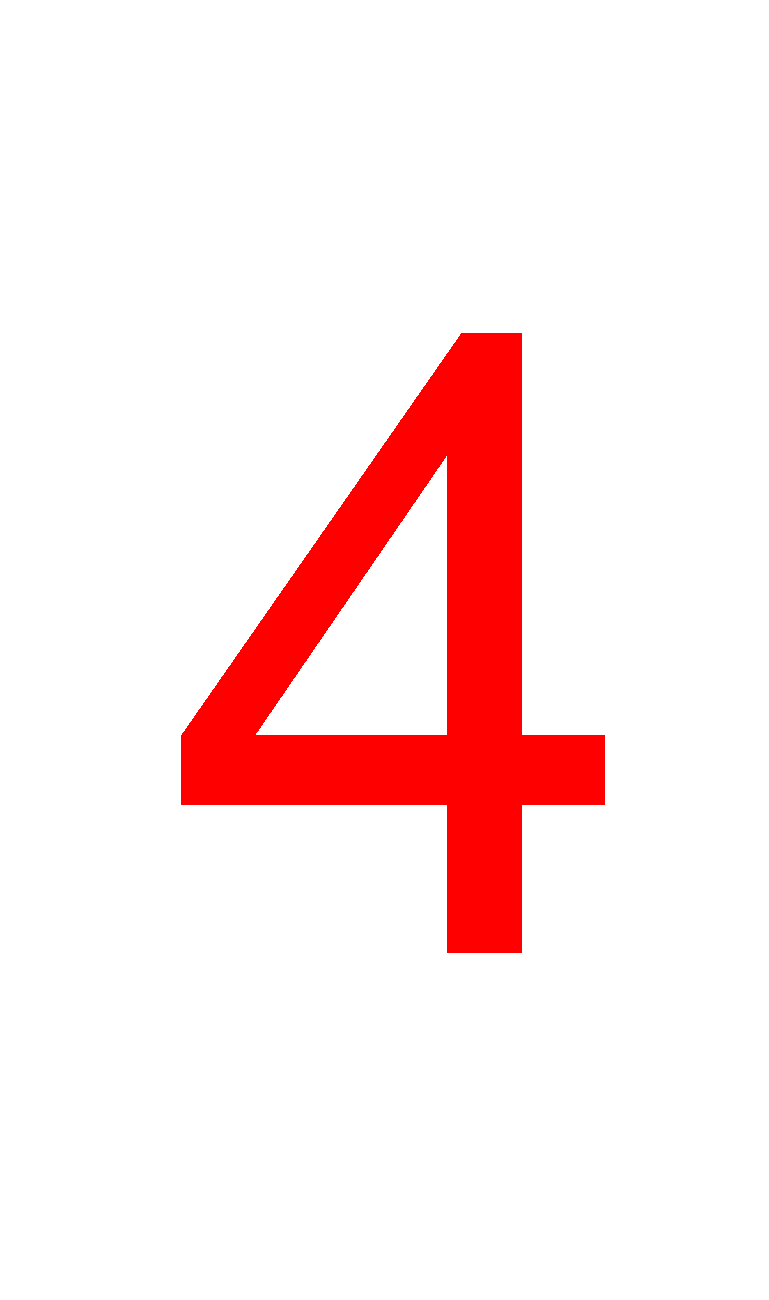

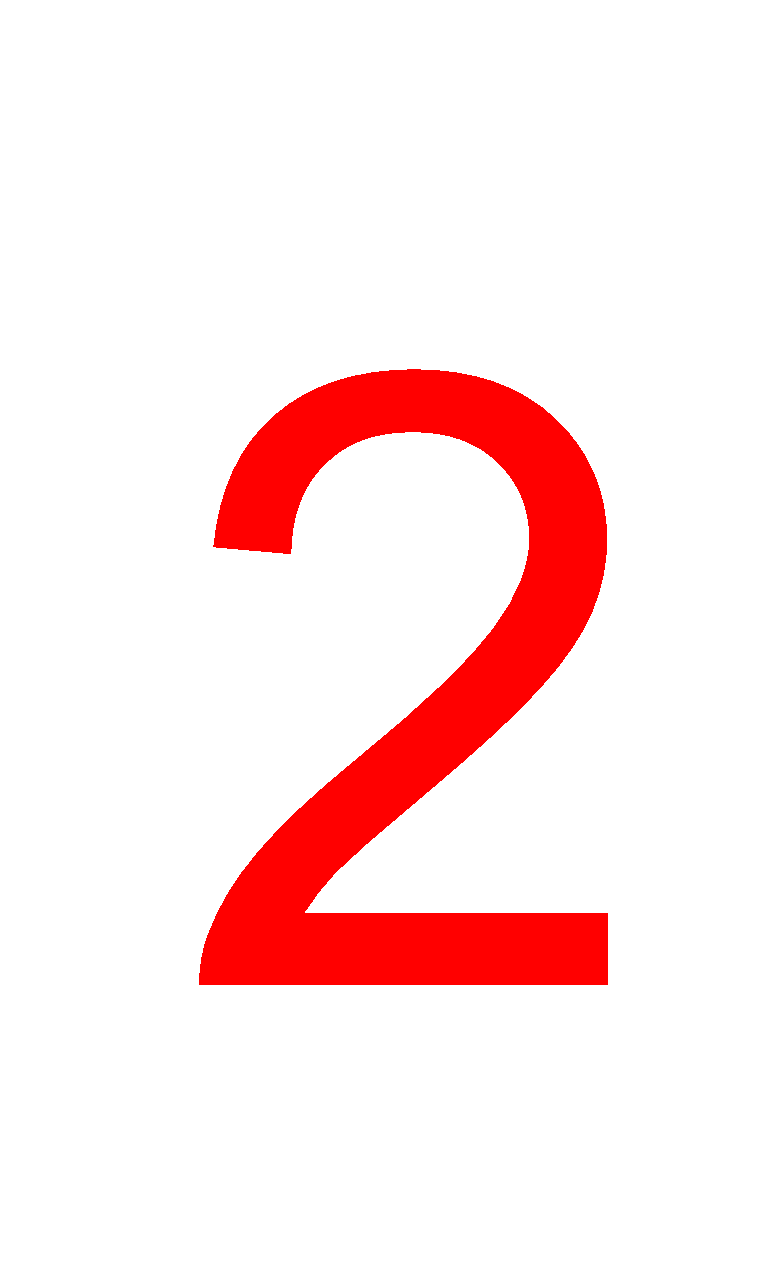

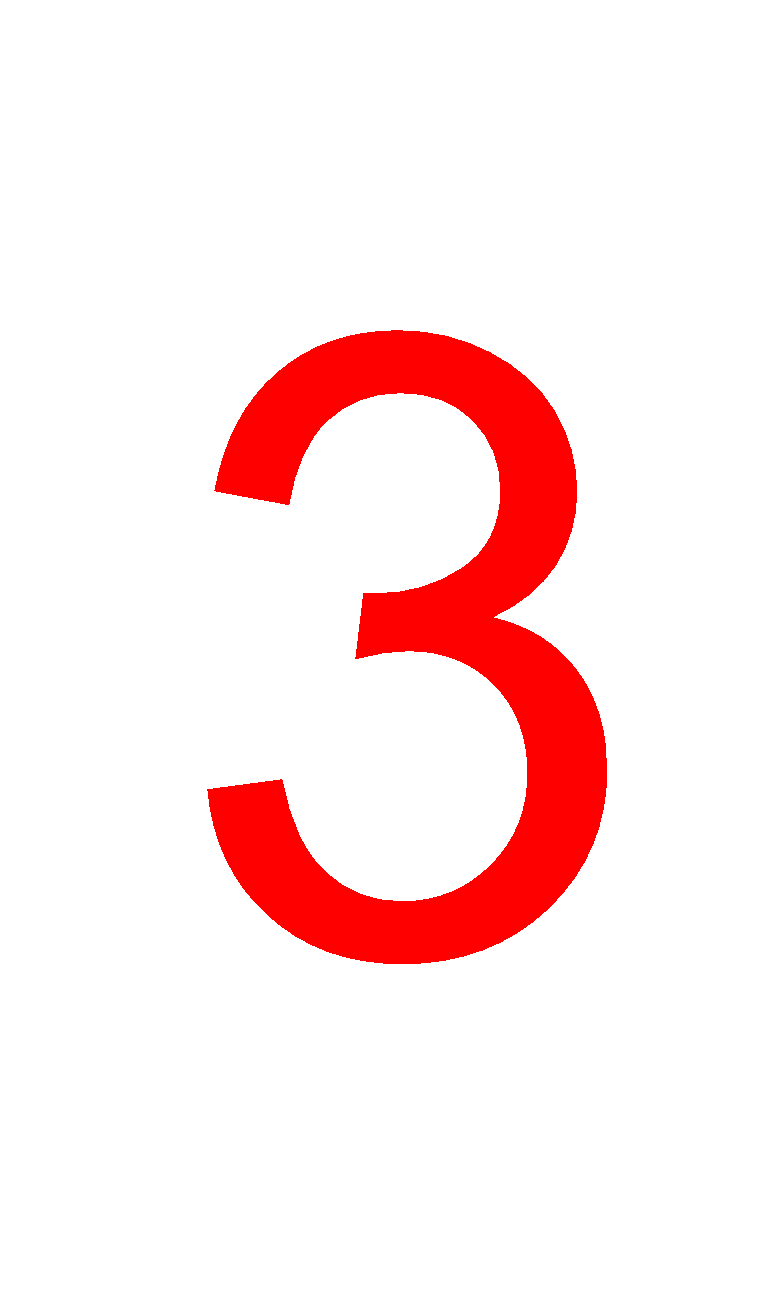

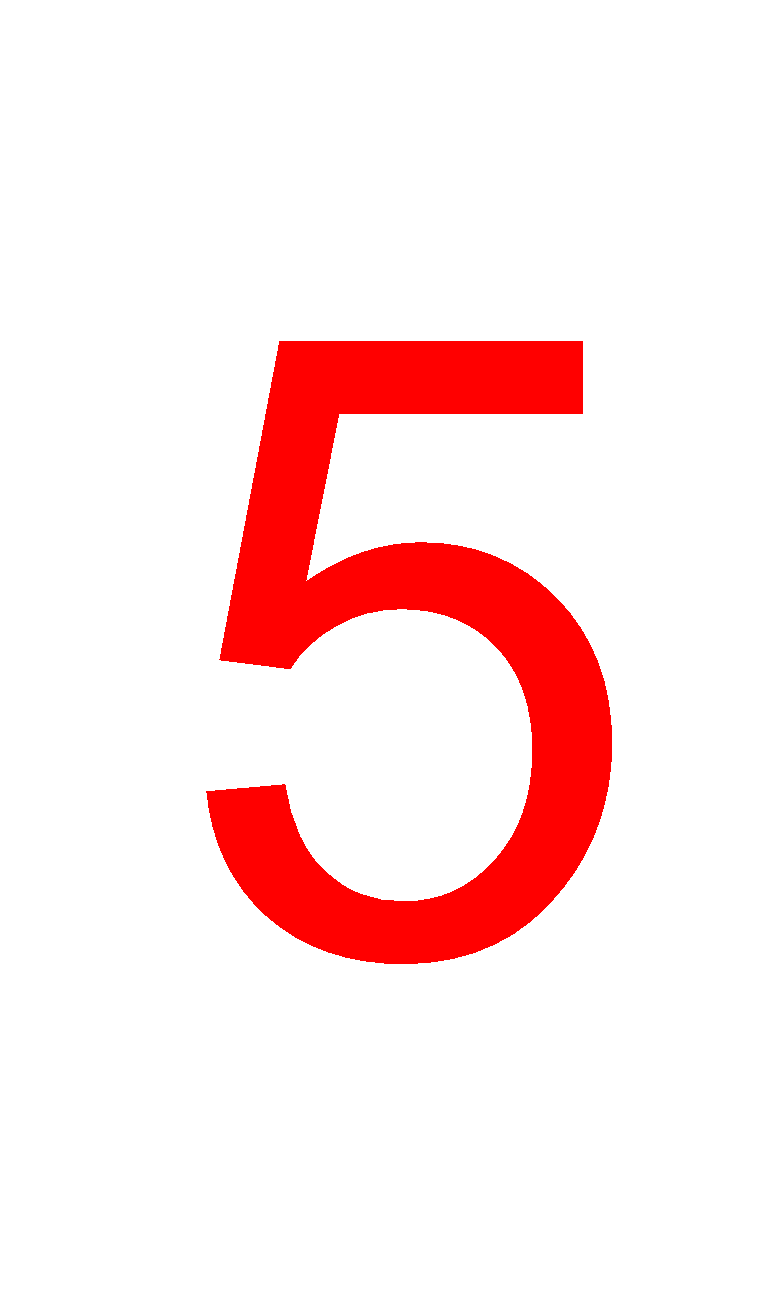

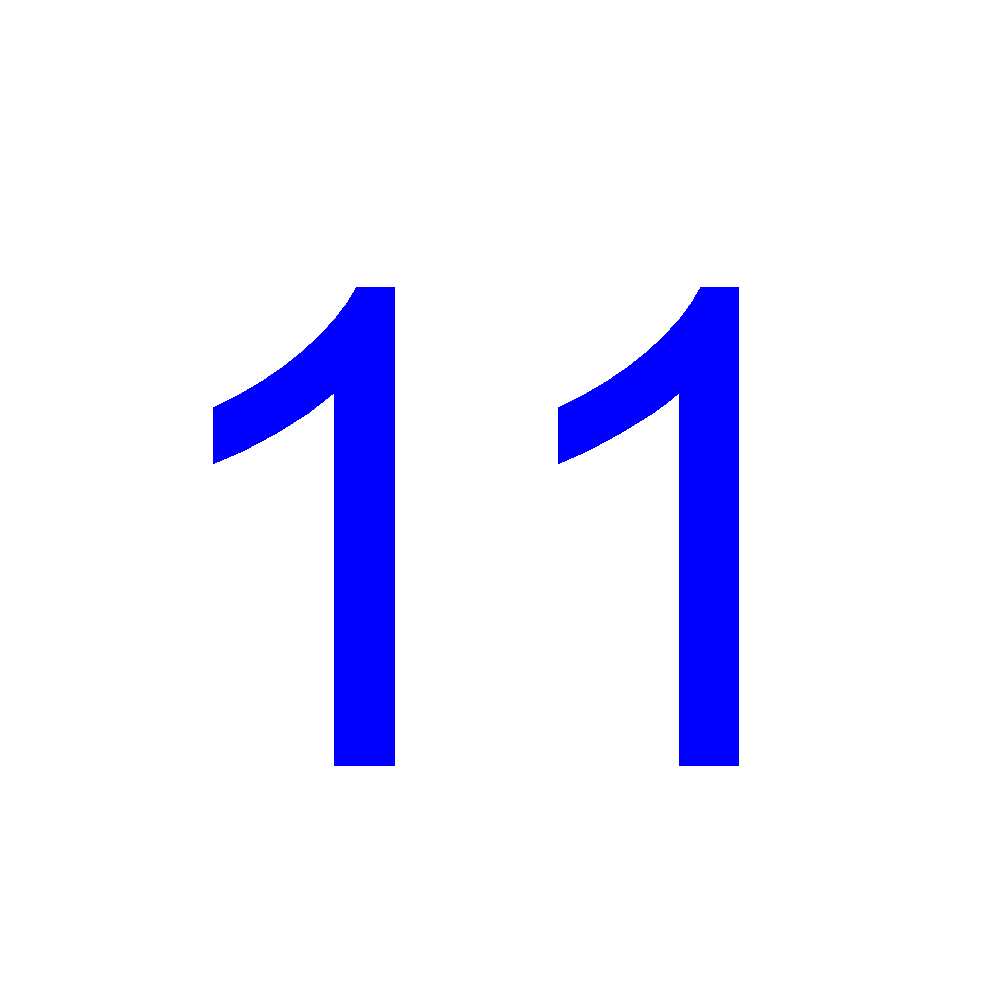

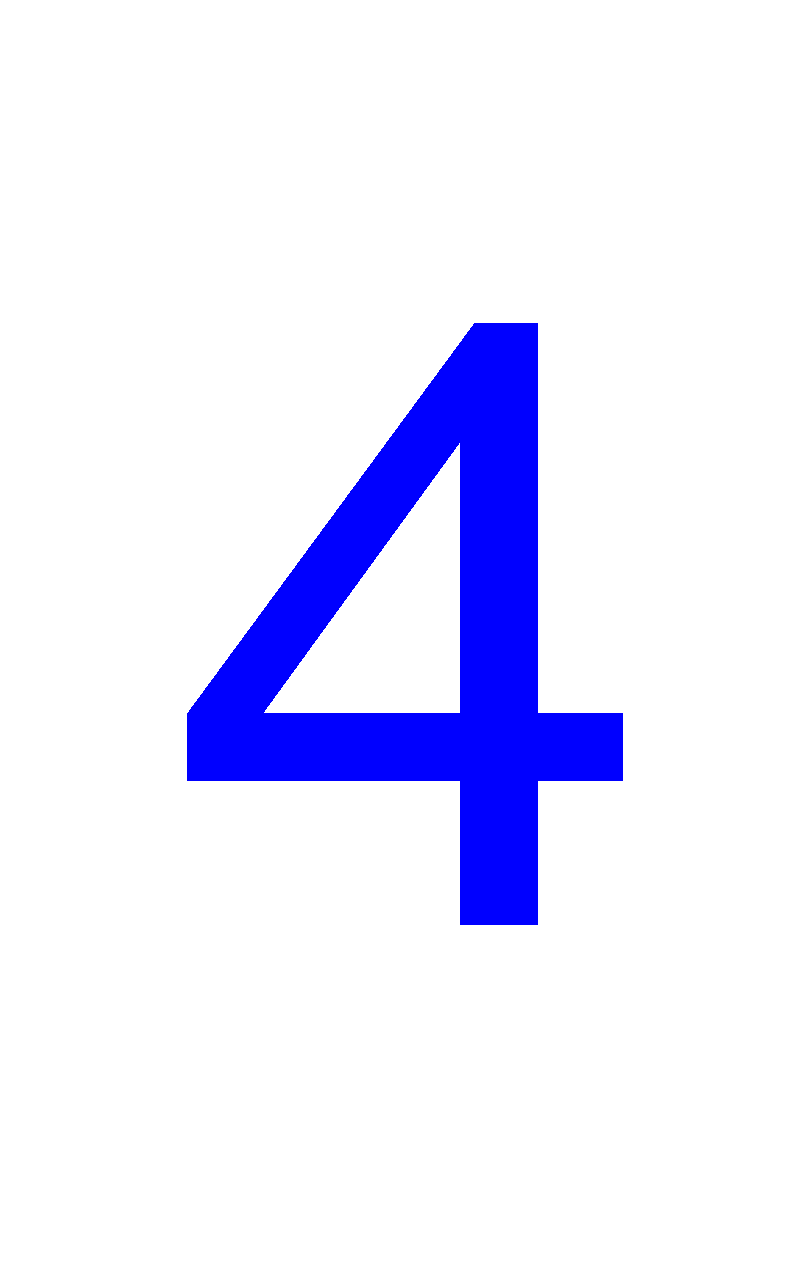

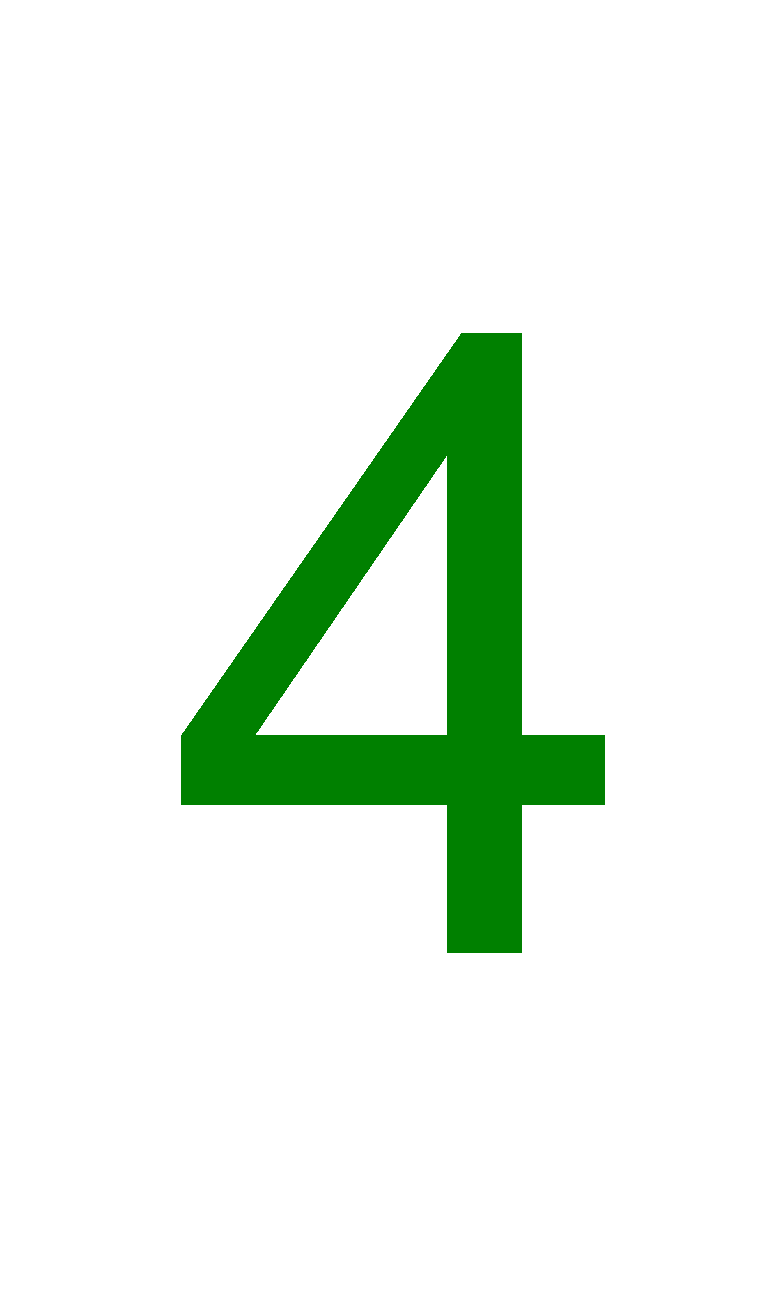

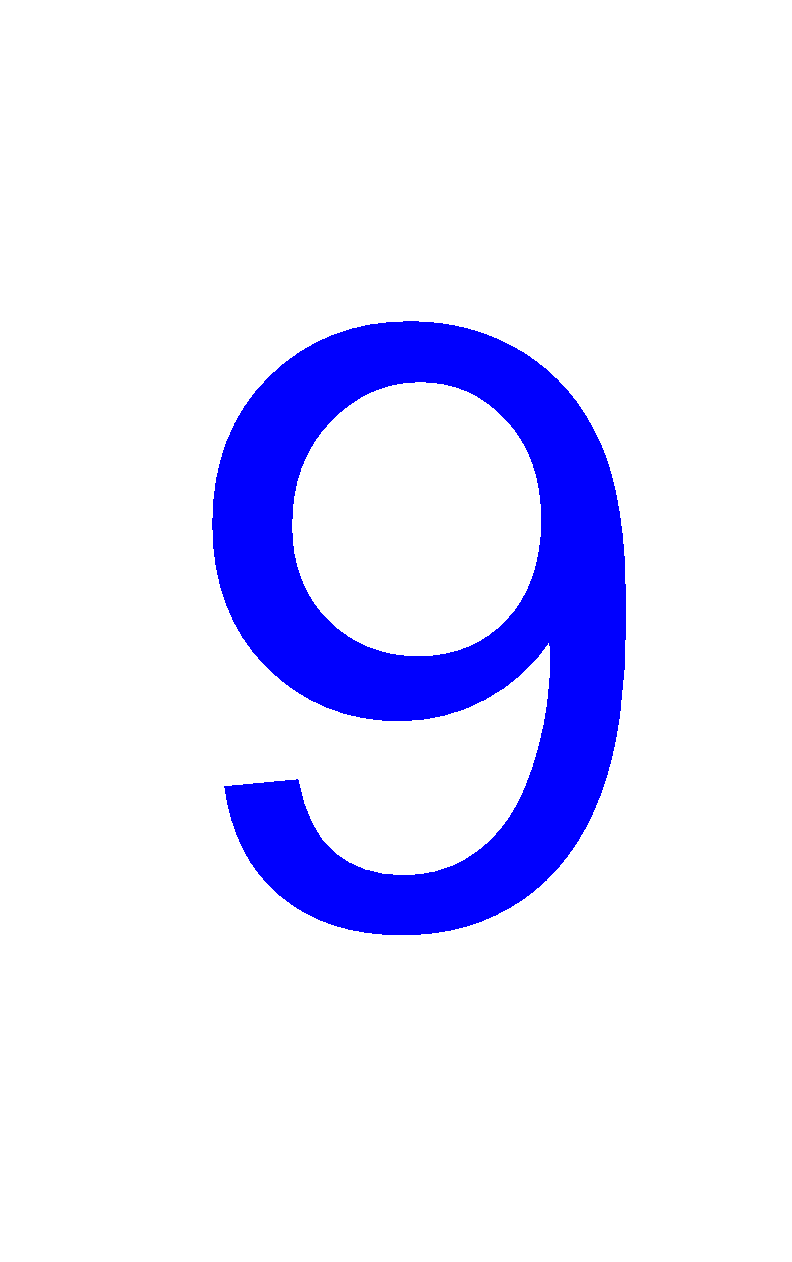

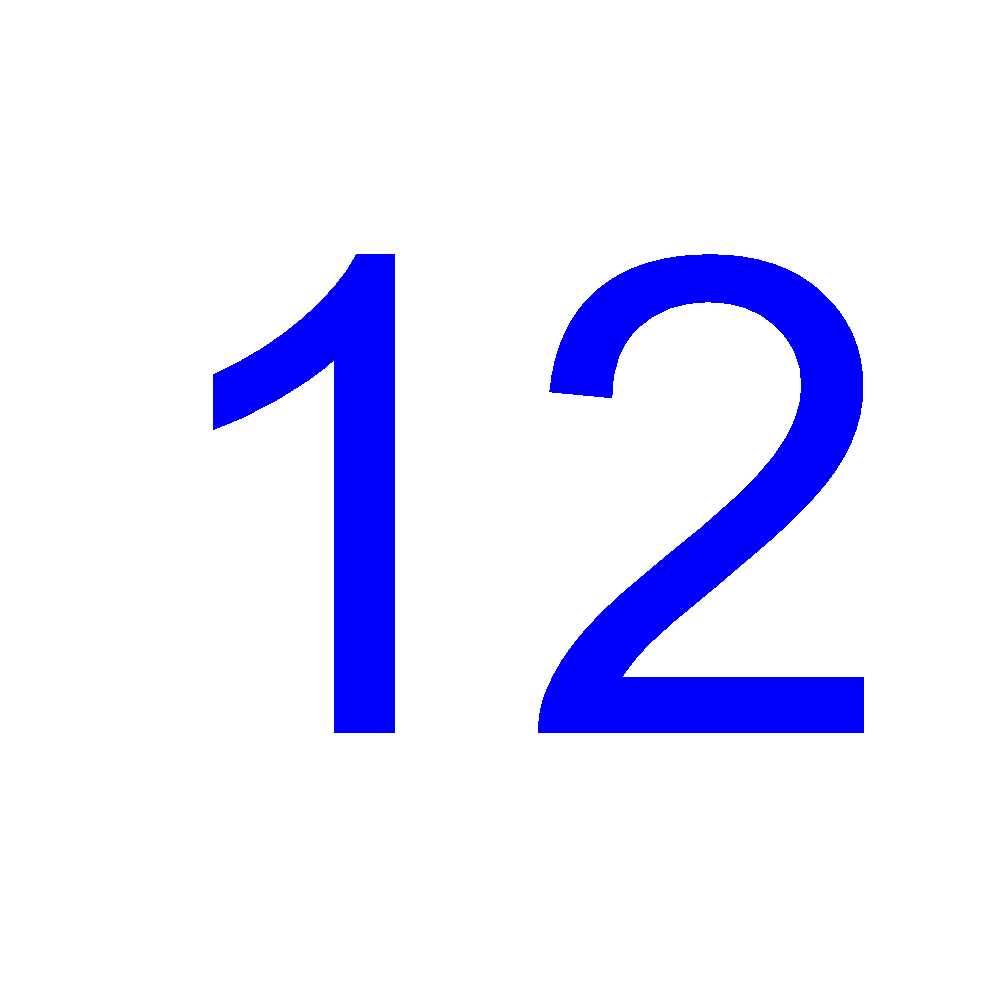

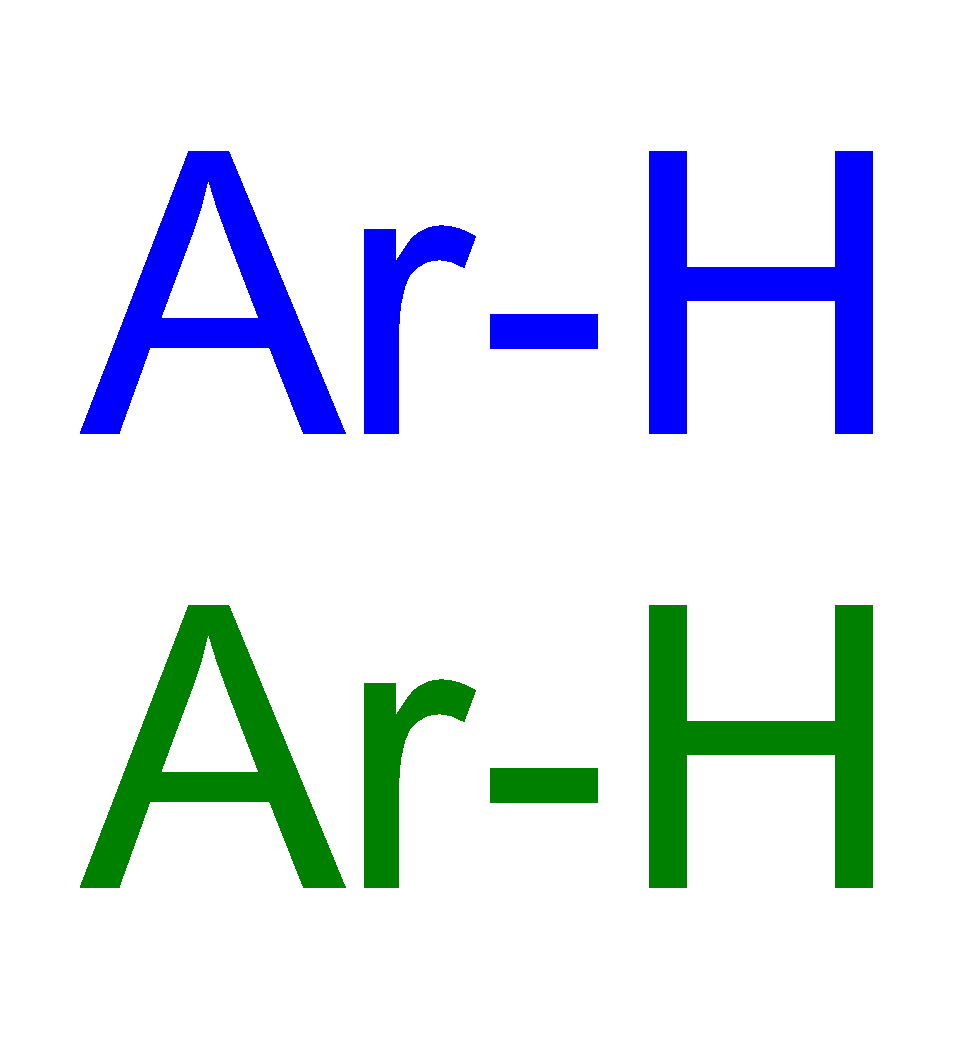

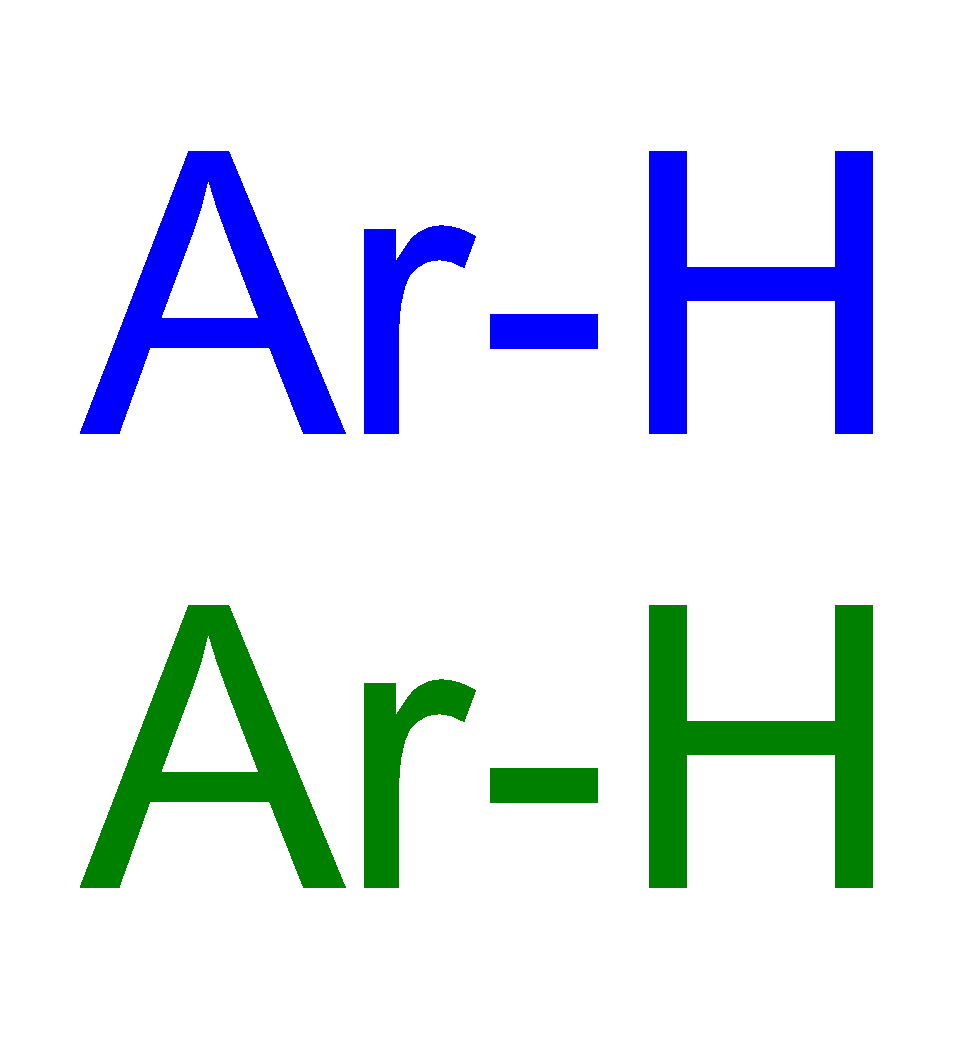

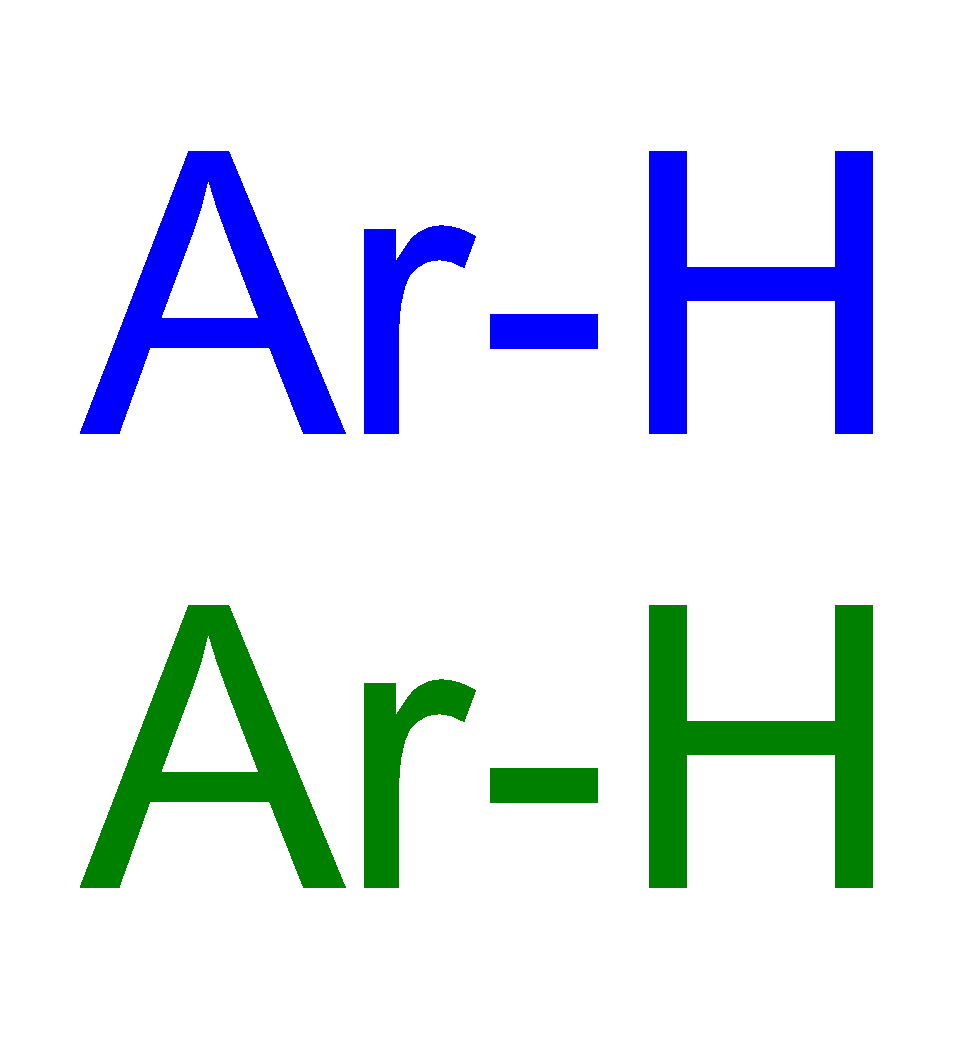


**Figure S5** –^1^H NMR of the isolated mixture. Zoom from 7.80 ppm to 6.85 ppm.


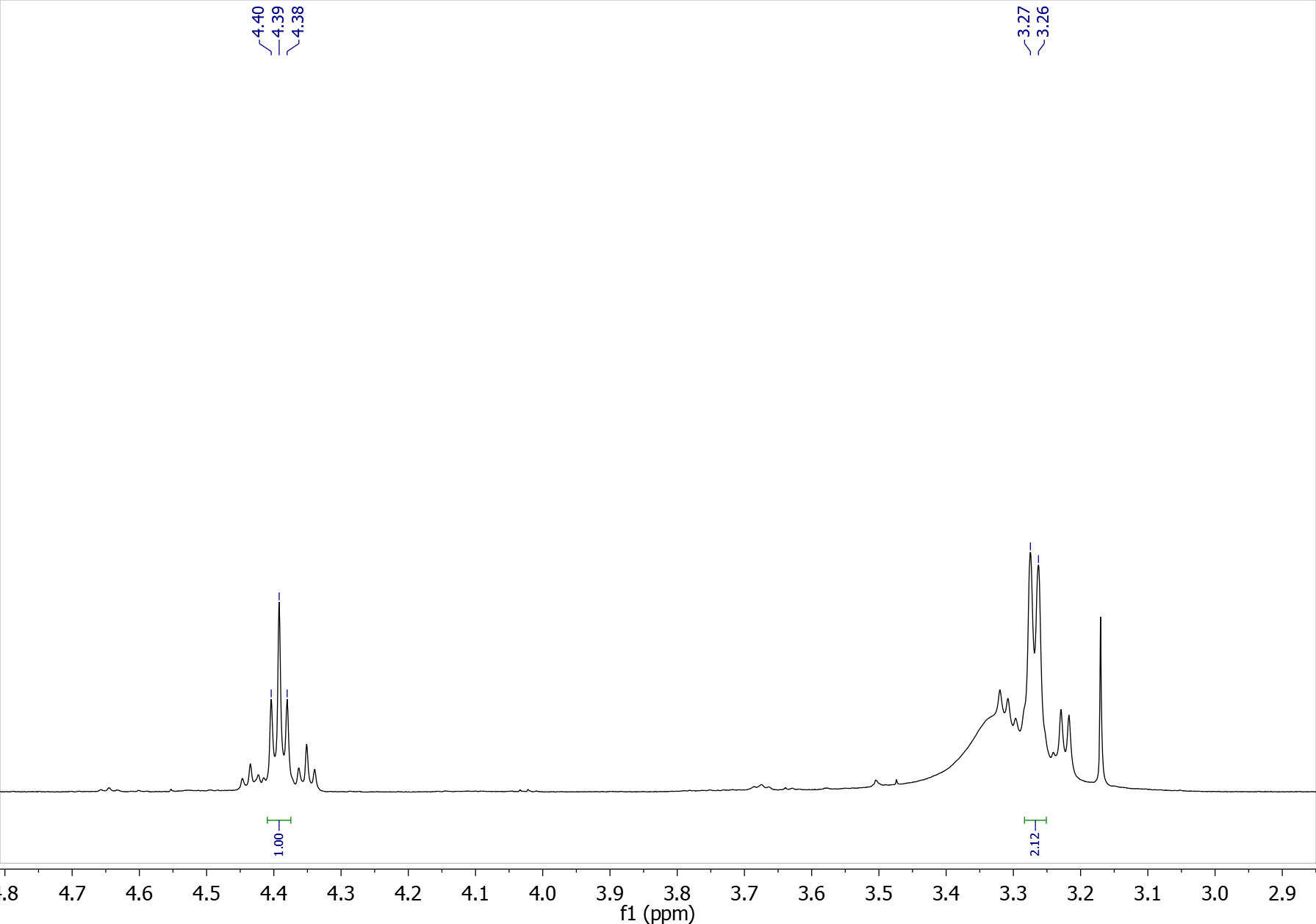

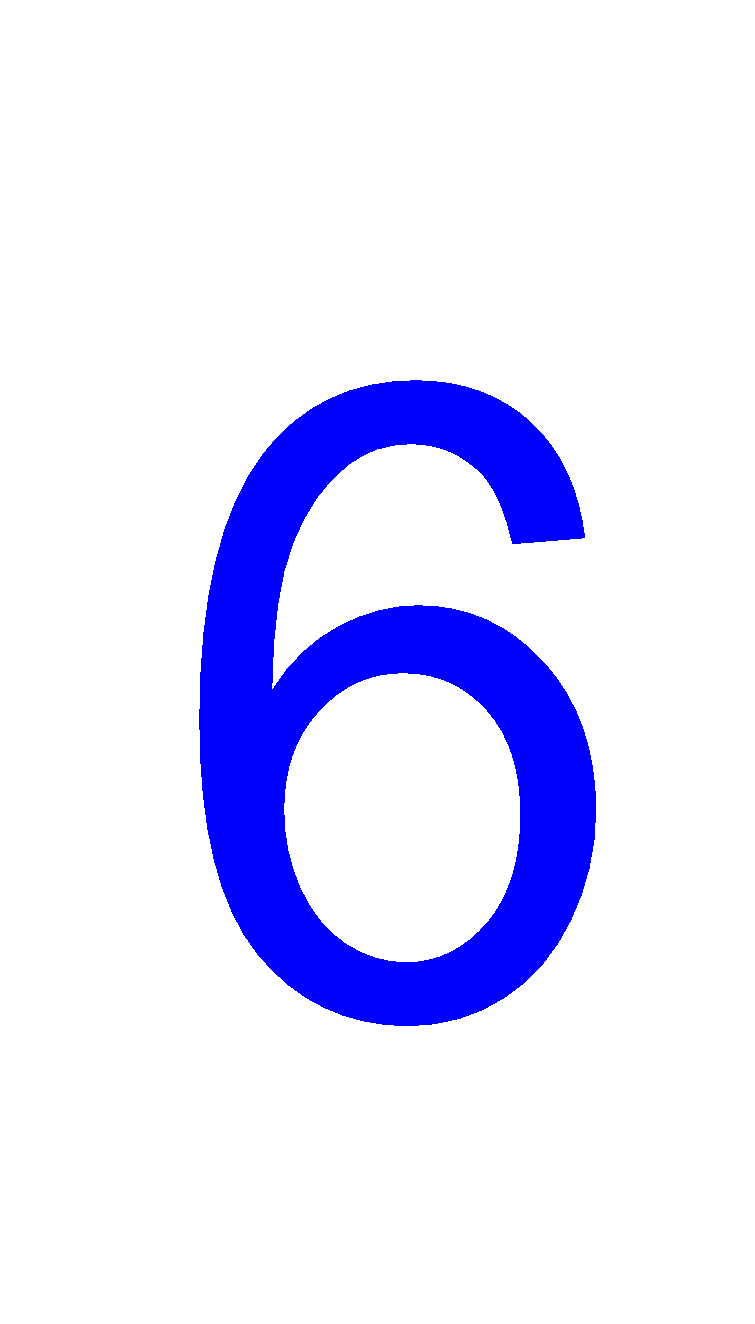

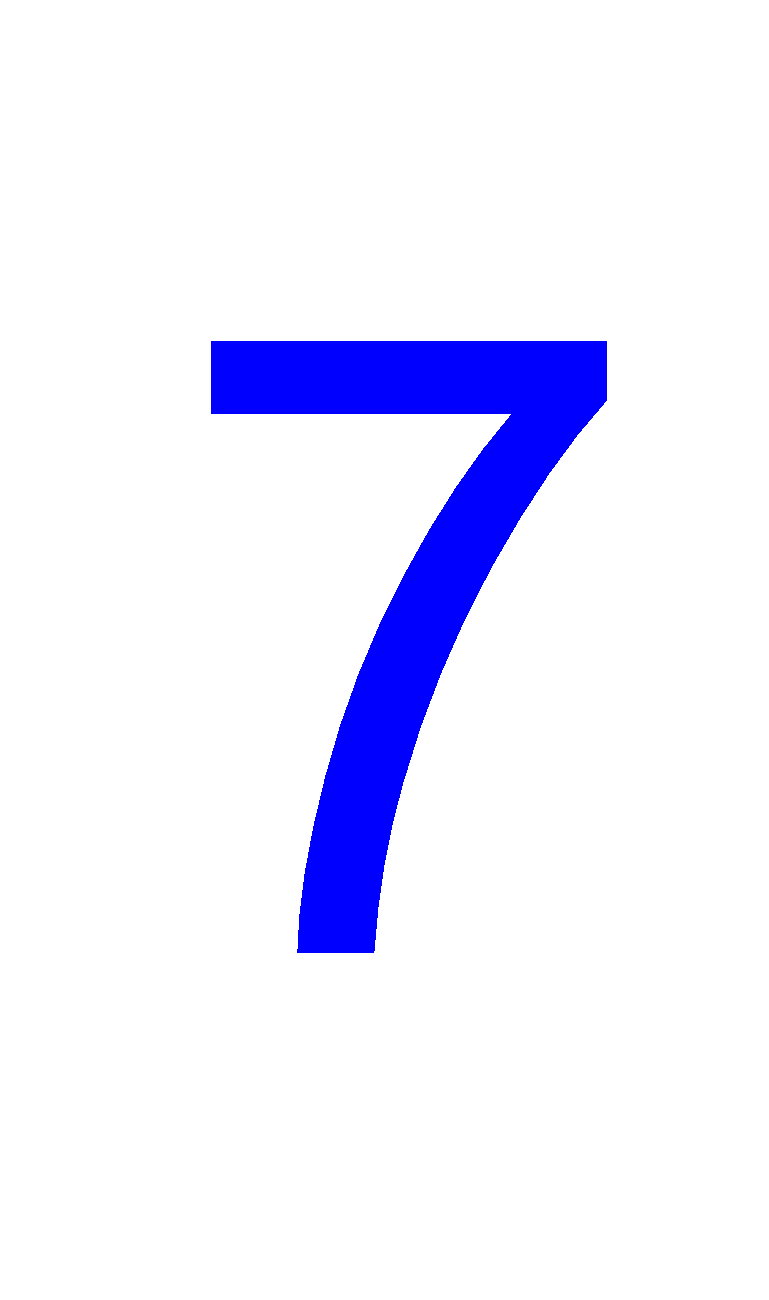

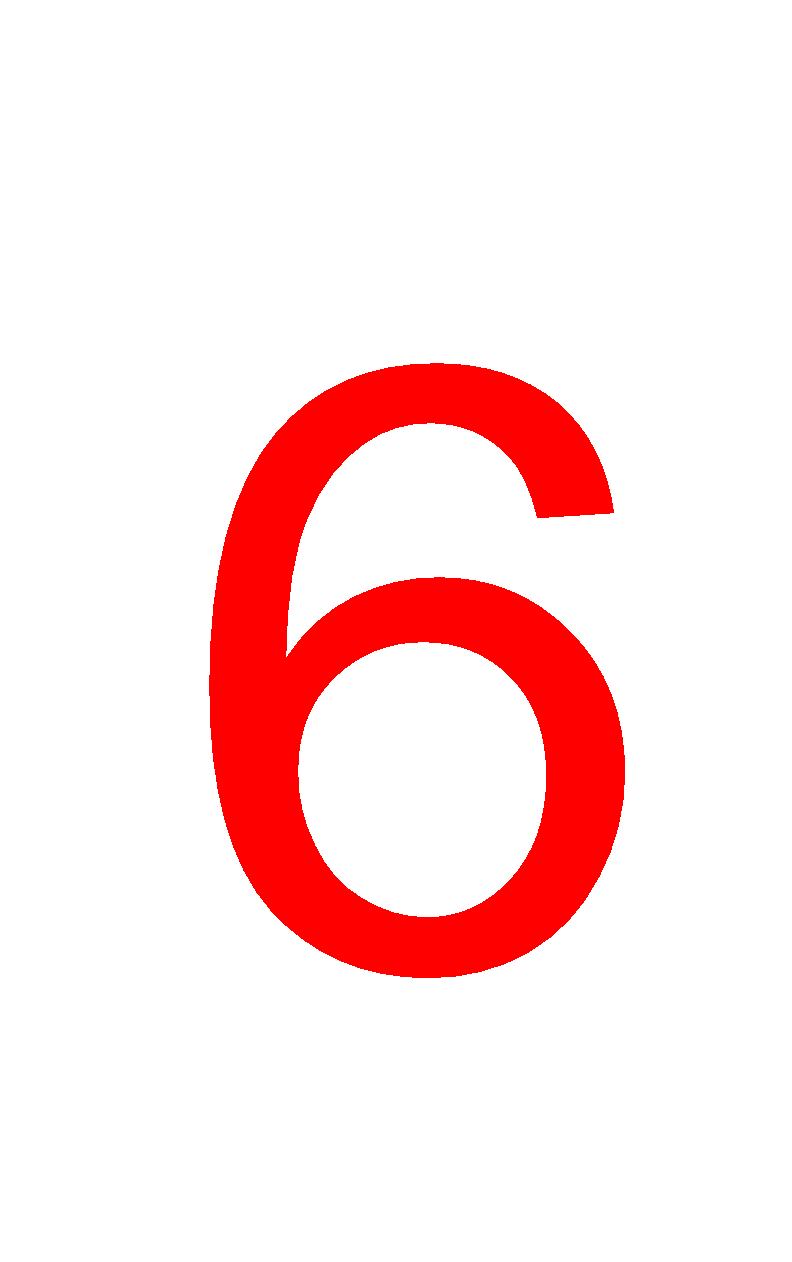

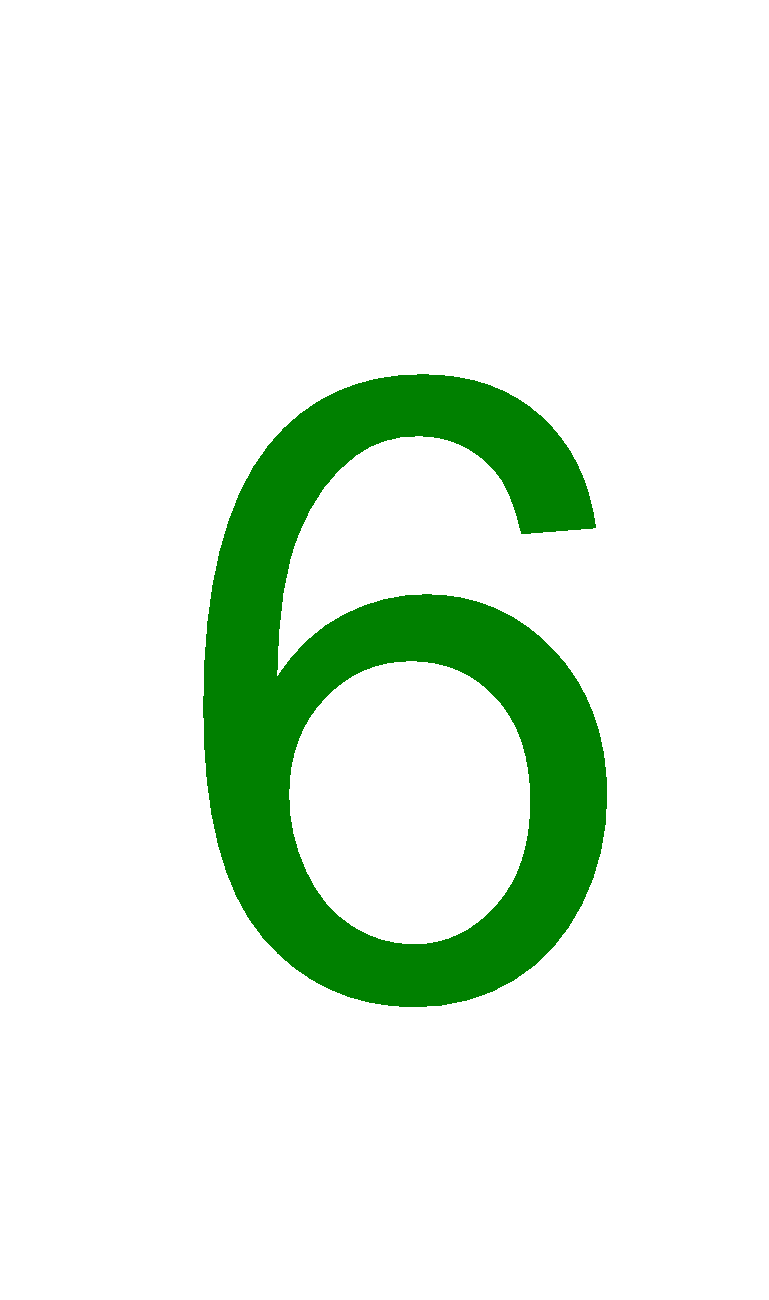

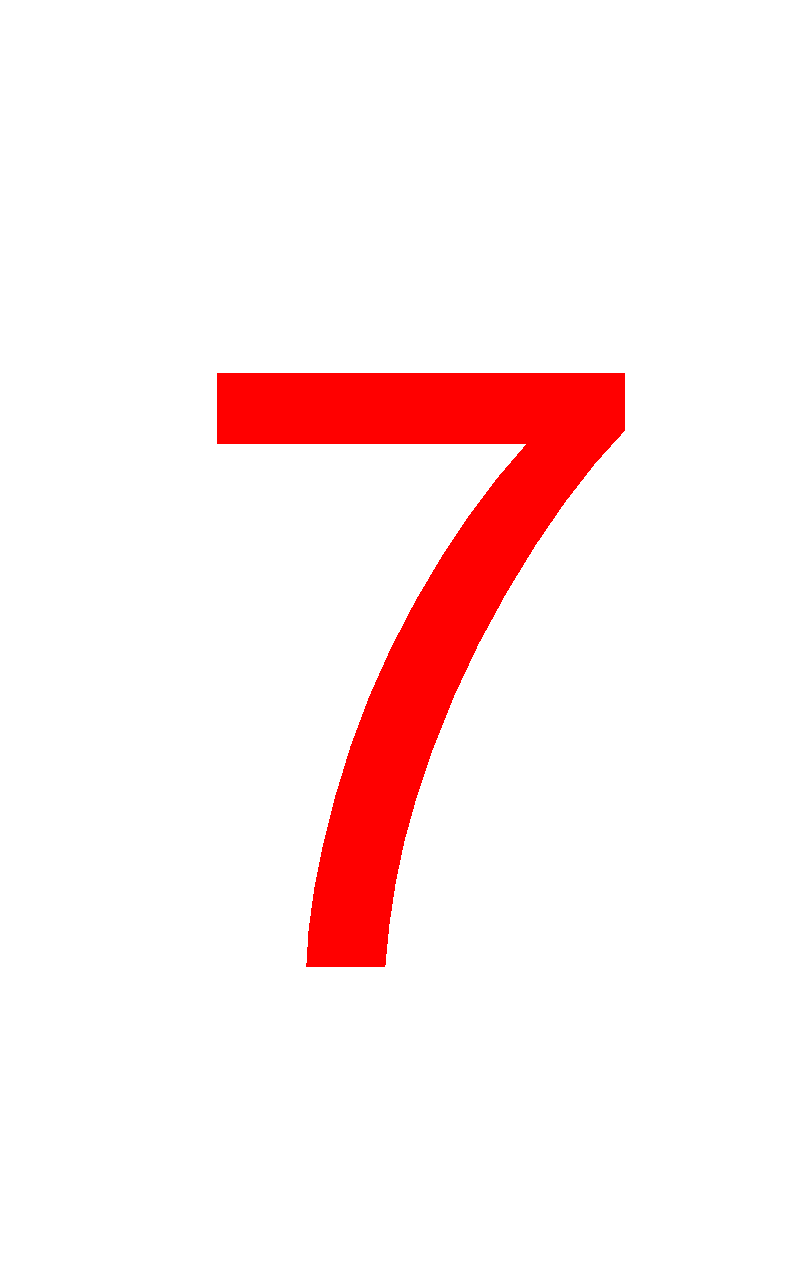

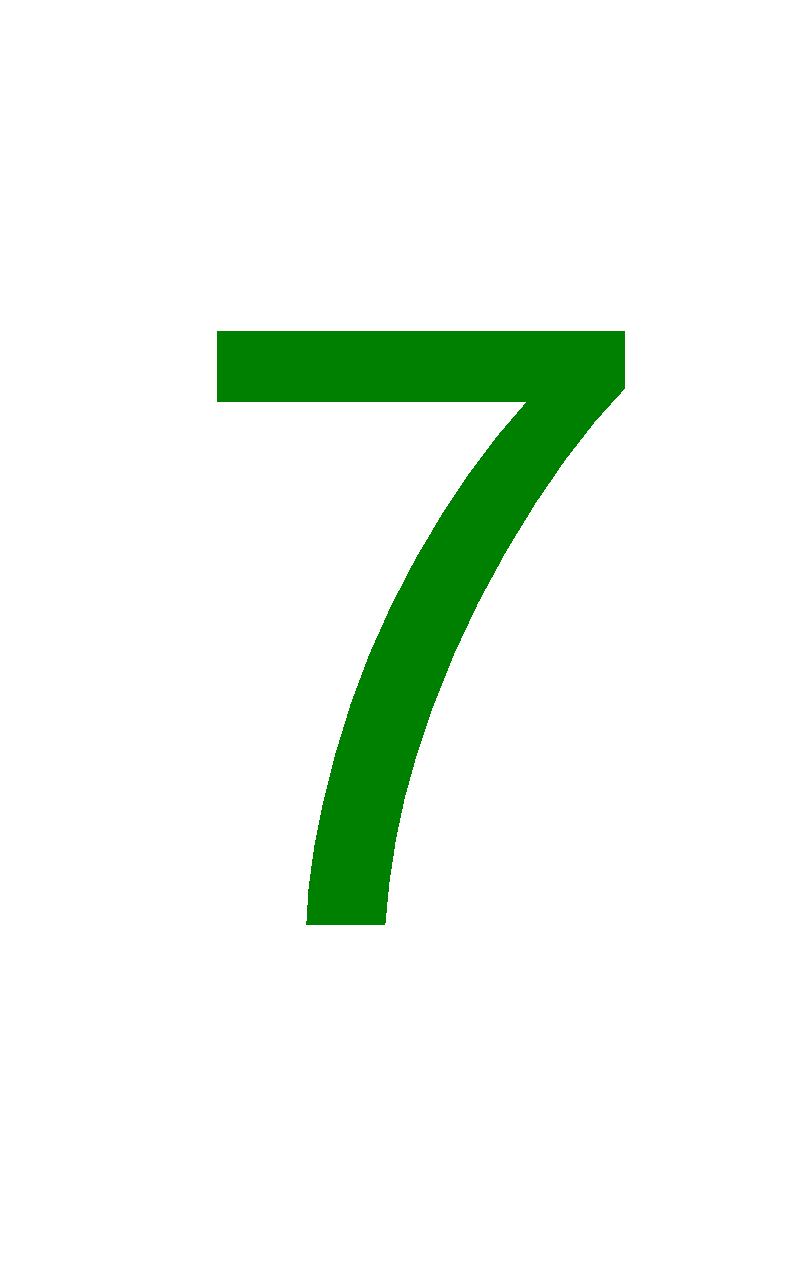


**Figure S6** –^1^H NMR of the isolated mixture. Zoom from 4.80 ppm to 2.90 ppm.


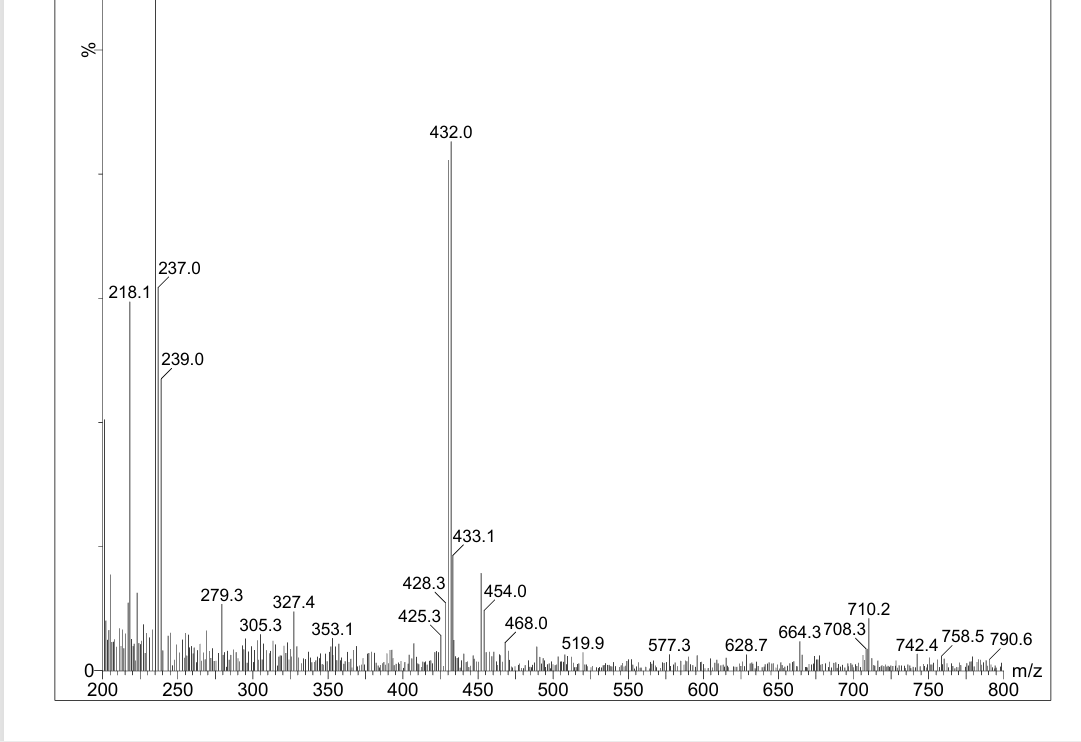


**Figure S7** – ESI(+)-MS spectra compound **4aa**.

Although compound **4aa** was isolated in a mixture, the pure mono-arylated compound **4am**, deriving from the coupling of the bisindole **1a** with the boronic acid **2m** was successfully isolated (*vide infra*).

1. **Synthesis of 2,2-bis(7-bromo-1*H*-indol-3-yl)ethan-1-amine 1c and 2,2-bis(4-bromo-1*H*-indol-3-yl)ethan-1-amine 1d**

The synthesis of the new bromobisindoles **1c** and **1d** has been realized following a literature procedure [1].

*Synthesis of the N-trifluoroacetamide bisindoles* ***Int-1c*** *and* ***Int-1d***

A mixture of diphenyl phosphate (0.1 equiv, 0.15 mmol, 37.5 mg), the appropriate bromoindole derivative (1.53 mmol, 300.0 mg) and (trifluoroacetylamino)acetaldehyde dimethyl acetal (0.5 equiv, 0.77 mmol, 154.0 mg) in anhydrous acetonitrile (0.77 mL) was stirred at 80 °C for 48 h in a sealed tube. After cooling to room temperature, saturated aqueous NaHCO_3_ solution (30 mL) and dichloromethane (30 mL) were added, and the two phases were separated. The aqueous solution was extracted with dichloromethane (3 x 20 mL). After drying over dry Na_2_SO_4_, the combined organic phases were concentrated in vacuo and the resulting crude product **Int-1c** or **Int-1d** was characterized and utilized without further purification.

(**Int-1c**): *R_f_* = 0.28 (cyclohexane/ethyl acetate, 50/50). ^1^H NMR (400 MHz, CDCl_3_): *δ* = 8.28 (br s, 2H, NH_ind_), 7.49 (d, *J* = 8.0 Hz, 2H, H4), 7.36 (d, *J* = 8.0 Hz, 2H, H6), 7.11 (d, *J* = 2.5 Hz, 2H, H2), 6.96 (dd, *J_1_* = *J_2_* = 8.0 Hz, 2H, H5), 6.38 (br s, 1H, N*H*COCF_3_), 4.76 (t, *J* = 7.0 Hz, 1H, C*H*CH_2_NH), 4.12 (t, *J* = 7.0 Hz, 2H, CHC*H_2_*NH) ppm. ^13^C NMR (101 MHz, CDCl_3_): *δ* = 157.5 (q, *J* = 37.0 Hz), 135.5, 127.7, 125.1, 122.7, 121.2, 118.7, 117.0, 115.9 (q, *J* = 289.0 Hz), 105.17, 43.8, 34.6 ppm. HRMS (m/z): [M+Na]^+^ calcd for C_20_H_14_Br_2_F_3_N_3_NaO, 549.9348; found 549.9343 [M+Na]^+^.

(**Int-1d**): *R_f_* = 0.26 (cyclohexane/ethyl acetate, 50/50). ^1^H NMR (400 MHz, CDCl_3_): *δ* = 8.16 (br s, 2H, NH_ind_), 7.30 (dd, *J_1_* = 1.0 Hz, *J_2_* = 8.0 Hz, 2H, H5), 7.27 (dd, *J_1_* = 1.0 Hz, *J_2_* = 8.0 Hz, 2H, H7), 7.02 (dd, *J_1_* = *J_2_* = 8.0 Hz, 2H, H6), 6.91 (d, *J* = 2.5 Hz, 2H, H2), 6.61 (br s, 1H, N*H*COCF_3_), 6.15 (t, *J* = 7.0 Hz, 1H, C*H*CH_2_NH), 4.13 (t, *J* = 7.0 Hz, 2H, CHC*H_2_*NH) ppm. ^13^C NMR (101 MHz, CDCl_3_): *δ* = 157.4 (q, *J* = 37.0 Hz), 138.3, 124.7, 124.6, 124.0, 123.3, 117.8, 116.0 (q, *J* = 289.0 Hz), 114.3, 110.8, 45.0, 33.4 ppm. HRMS (m/z): [M+Na]^+^ calcd for C_20_H_14_Br_2_F_3_N_3_NaO, 549.9348; found 549.9344 [M+Na]^+^.

*Synthesis of bisindoles* ***1c*** *and* ***1d***

A mixture of crude trifluoroacetamide derivative **Int-1c** or **Int-1d** and potassium carbonate (2.5 equiv, 3.8 mmol, 525.2 mg) in MeOH (7 mL) and H_2_O (0.5 mL) was stirred and heated under reflux for 2 h. The solvent was removed under reduced pressure and water was added (30 mL). The aqueous solution was extracted with dichloromethane (3 x 30 mL) and the resulting solution was dried with Na_2_SO_4_ and concentrated in vacuo. The crude material was purified by flash chromatography.

Compound **1c** has been purified by flash column chromatography on silica gel (chloroform/methanol/ammonia, 99/1/1), obtaining a white solid; yield: 55% (182 mg). *R*_f_ = 0.33 (silica gel; chloroform/methanol/ammonia, 90/10/1). ^1^H NMR (400 MHz, DMSO-*d*_6_): *δ* = 11.06 (br s, 2H, NH_ind_), 7.50 (d, *J* = 8.0 Hz, 2H, H4), 7.29 (d, *J* = 2.0 Hz, 2H, H2), 7.23 (d, *J* = 8.0 Hz, 2H, H6), 6.84 (dd, *J_1_* = *J_2_* = 8.0 Hz, 2H, H5), 4.39 (t, *J* = 7.0 Hz, 1H, C*H*CH_2_NH_2_), 3.26 (d, *J* = 7.0 Hz, 2H, CHC*H_2_*NH_2_) ppm. ^13^C NMR (101 MHz, DMSO-*d_6_*): *δ* = 134.6, 128.6, 123.7, 123.3, 119.6, 118.5, 118.1, 104.2, 46.5, 38.0 ppm. HRMS (m/z): [M+H]^+^ calcd for C_18_H_16_Br_2_N_3_, 431.9705; found 431.9713.

Compound **1d** has been purified by flash column chromatography on silica gel (chloroform/methanol/ammonia, 99/1/1), obtaining an off white solid; yield: 52% (171 mg). *R*_f_ = 0.23 (silica gel; chloroform/methanol/ammonia, 90/10/1). ^1^H NMR (400 MHz, DMSO-*d*_6_): *δ* = 11.15 (br s, 2H, NH_ind_), 7.36 (dd, *J_1_* = 0.5 Hz, *J_2_* = 8.0 Hz, 2H, H5), 7.11 (dd, *J_1_* = 0.5 Hz, *J_2_* = 8.0 Hz, 2H, H7), 6.98 (br s, 2H, H2), 6.94 (dd, *J_1_* = *J_2_* = 8.0 Hz, H6), 5.71 (t, *J* = 7.0 Hz, 1H, C*H*CH_2_NH_2_), 3.16 (d, *J* = 7.0 Hz, 2H, CHC*H_2_*NH_2_) ppm. ^13^C NMR (101 MHz, DMSO-*d_6_*): *δ* = 138.3, 124.8, 124.6, 122.8, 122.0, 118.2, 113.3, 111.3, 48.9, 37.0 ppm. HRMS (m/z): [M+H]^+^ calcd for C_18_H_16_Br_2_N_3_, 431.9705; found 431.9721.

1. **Experimental data for compounds 3 and 4**

**2,2-bis(6-phenyl-1*H*-indol-3-yl)ethan-1-amine (3aa):**

From boronic acid **2a**: Following the general procedure (reaction time = 1h), compound **3aa** has been purified by flash column chromatography on silica gel (dichloromethane/methanol/ammonia, 99/1/1), obtaining a white solid; yield: 79% (34 mg).

From phenylboronic acid pinacol ester **2a’**: Following the general procedure (reaction time = 4h), compound **3aa** was obtained by flash column chromatography on silica gel (dichloromethane/methanol/ammonia, 99/1/1) using phenylboronic acid pinacol ester in place of the corresponding boronic acid; yield: 74% (31mg).

*R_f_* = 0.38 (silica gel; dichloromethane/methanol/ammonia, 90/10/1). ^1^H NMR (400 MHz, DMSO-*d_6_*): *δ* = 10.91 (br d, *J* = 2.5 Hz, 2H, NH_ind_), 7.63-7.61 (m, 4H, Ar-H), 7.56 (d, *J* = 8.0 Hz, 2H Ar-H), 7.55 (s, 2H, Ar-H), 7.44-7.40 (m, 4H Ar-H), 7.33-7.25 (m, 4H, Ar-H), 7.19 (dd, *J_1_* = 1.5 Hz, *J_2_* = 8.0 Hz, 2H, Ar-H), 4.43 (t, *J* = 7.0 Hz, 1H, C*H*CH_2_NH_2_), 3.30 (d, *J* = 7.0 Hz, 2H, CHC*H_2_*NH_2_), 1.45 (br s, 2H, CHCH_2_N*H_2_*) ppm. ^13^C NMR (101 MHz, DMSO-*d_6_*): *δ* = 141.8, 137.1, 133.6, 128.8, 126.7, 126.4, 126.3, 123.2, 119.4, 117.5, 116.9, 109.4, 46.7, 38.1 ppm. HRMS (m/z): [M+H]^+^ calcd for C_30_H_26_N_3_, 428.2121; found 428.2121.

**2,2-bis(6-(4-fluorophenyl)-1*H*-indol-3-yl)ethan-1-amine (3ab):**

Following the general procedure (reaction time = 45 min), compound **3ab** has been purified by flash column chromatography on silica gel (dichloromethane/methanol/ammonia, 99/1/1), obtaining a white solid; yield: 82% (38 mg). *R*_f_ = 0.28 (silica gel; dichloromethane/methanol/ammonia, 90/10/1). ^1^H NMR (400 MHz, DMSO-*d_6_*): *δ* = 10.94 (br s, 2H, NH_ind_), 7.66-7.62 (m, 4H, Ar-H), 7.55 (d, *J* = 8.5 Hz, 2H, Ar-H), 7.53 (d, *J* = 1.5 Hz, 2H, Ar-H), 7.30 (d, *J* = 2.5 Hz, 2H, Ar-H), 7.26-7.22 (m, 4H, Ar-H), 7.16 (dd, *J_1_* = 1.5 Hz, *J_2_* = 8.5 Hz, 2H, Ar-H), 4.43 (t, *J* = 7.0 Hz, 1H, C*H*CH_2_NH_2_), 3.31 (d, *J* = 7.0 Hz, 2H, CHC*H_2_*NH_2_), 1.54 (br s, 2H, CHCH_2_N*H_2_*) ^13^C NMR (101 MHz, DMSO-*d_6_*): *δ* = 161.3 (d, *J* = 244.0), 138.3 (d, *J* = 3.0 Hz), 137.1, 132.3, 128.5 (d, *J* = 8.0 Hz), 126.3, 123.3, 119.5, 117.5, 116.9, 115.5 (d, *J* = 21.0 Hz), 109.4, 46.7, 38.0 ppm. HRMS (m/z): [M+H]^+^ calcd for C_30_H_24_F_2_N_3_, 464.1933; found 464.1937.

**2,2-bis(6-(4-isobutylphenyl)-1*H*-indol-3-yl)ethan-1-amine (3ac):**

Following the general procedure (reaction time = 2h), compound **3ac** has been purified by flash column chromatography on silica gel (dichloromethane/methanol/ammonia, 99/1/1), obtaining a white solid; yield: 76% (41 mg). *R*_f_ = 0.39 (silica gel; dichloromethane/methanol/ammonia, 90/10/1). ^1^H NMR (400 MHz, DMSO-*d_6_*): *δ* = 10.88 (br d, *J* = 2.5 Hz, 2H, NH_ind_), 7.56-7.50 (m, 8H, Ar-H), 7.28 (d, *J* = 2.5 Hz, 2H, H2_ind_), 7.22-7.15 (m, 6H, Ar-H), 4.42 (t, *J* = 7.0 Hz, 1H, C*H*CH_2_NH_2_), 3.30 (d, *J* = 6.5 Hz, 2H, CHC*H_2_*NH_2_) 2.46 (d, *J* = 7.0 Hz, 2H, C*H_2_*CH(CH_3_)_2_), 1.85 (hept, *J* = 7.0 Hz, 2H, CH_2_C*H*(CH_3_)_2_), 0.89 (d, *J* = 6.5 Hz, 12H, CH_2_CH*(CH_3_)_2_*) ppm. ^13^C NMR (101 MHz, DMSO-*d_6_*): *δ* = 139.3, 139.2, 137.2, 133.3, 129.4, 126.4, 126.2, 123.1, 119.4, 117.4, 116.8, 109.1, 46.6, 44.2, 37.9, 29.6, 22.2 ppm. HRMS (m/z): [M+H]^+^ calcd for C_38_H_42_N_3_, 540.3373; found 540.3374.

**2,2-bis(6-(4-methoxyphenyl)-1*H*-indol-3-yl)ethan-1-amine (3ad):**

Following the general procedure (reaction time = 2h), compound **3ad** has been purified by flash column chromatography on silica gel (dichloromethane/methanol/ammonia, 99/1/1), obtaining a white solid; yield: 86% (42 mg). *R*_f_ = 0.33 (silica gel; dichloromethane/methanol/ammonia, 90/10/1). ^1^H NMR (400 MHz, DMSO-*d_6_*): *δ* = 10.85 (br d, *J* = 2.5 Hz, 2H, NH_ind_), 7.56-7.50 (m, 6H, Ar-H), 7.48 (s, 2H, Ar-H), 7.25 (d, *J* = 2.5 Hz, 2H, H2_ind_), 7.14 (dd, *J_1_* = 1.5 Hz, *J_2_* = 8.5 Hz, 2H, Ar-H), 7.01-6.97 (m, 4H, Ar-H), 4.41 (t, *J* = 7.0 Hz, 1H, C*H*CH_2_NH_2_), 3.77 (s, 6H, OC*H_3_*), 3.30 (d, *J* = 7.0 Hz, 2H, CHC*H_2_*NH_2_), 1.85 (br s, 2H, CHCH_2_N*H_2_*) ppm. ^13^C NMR (101 MHz, DMSO-*d_6_*): *δ* = 158.2, 137.2, 134.2, 133.1, 127.6, 125.9, 122.9, 119.3, 117.3, 116.7, 114.3, 108.8, 55.1, 46.5, 37.9 ppm. HRMS (m/z): [M+H]^+^ calcd for C_32_H_30_N_3_O_2_, 488.2333; found 488.2336.

**2,2-bis(6-(4-((tert-butyldimethylsilyl)oxy)phenyl)-1*H*-indol-3-yl)ethan-1-amine (3ae):**

Following the general procedure (reaction time = 1h), compound **3ae** has been purified by flash column chromatography on silica gel (dichloromethane/methanol/ammonia, 99/1/1), obtaining a white solid; yield: 66% (45 mg). *R*_f_ = 0.43 (silica gel; dichloromethane/methanol/ammonia, 90/10/1). ^1^H NMR (400 MHz, DMSO-*d_6_*): *δ* = 10.86 (br d, *J* = 2.5 Hz, 2H, NH_ind_), 7.55-7.47 (m, 8H, Ar-H), 7.26 (d, *J* = 2.5 Hz, 2H, H2_ind_), 7.14 (dd, *J_1_* = 1.5 Hz, *J_2_* = 8.0 Hz, 2H, Ar-H), 6.90 (m, 4H, Ar-H), 4.42 (t, *J* = 7.0 Hz, 1H, C*H*CH_2_NH_2_), 3.31 (d, *J* = 7.0 Hz, 2H, CHC*H_2_*NH_2_), 0.97 (s, 18H, Si*t*-Bu), 0.21 (s, 12H, Si(CH_3_)_2_) ppm. ^13^C NMR (101 MHz, DMSO-*d_6_*): *δ* = 154.0, 137.2, 135.1, 133.0, 127.7, 125.9, 123.0, 120.1, 119.3, 117.3, 116.7, 108.9, 46.5, 37.7, 25.6, 18.0, -4.5. HRMS (m/z): [M+H]^+^ calcd for C_42_H_54_N_3_O_2_Si_2_, 688.3749; found 688.3740.

**2,2-bis(6-(4-vinylphenyl)-1*H*-indol-3-yl)ethan-1-amine (3af):**

Following the general procedure (reaction time = 1h), compound **3af** has been purified by flash column chromatography on silica gel (dichloromethane/ammonia, 99/1 to dichloromethane/methanol/ammonia, 99/1/1), obtaining a yellow solid; yield: 29% (14 mg). *R_f_* = 0.32 (silica gel; dichloromethane/methanol/ammonia, 90/10/1). ^1^H NMR (400 MHz, CDCl_3_): *δ* = 8.12 (br s, 2H, NH_ind_), 7.66 (d, *J* = 8.5 Hz, 2H, Ar-H), 7.61-7.55 (m, 6H, Ar-H), 7.48-7.46 (m, 4H, Ar-H), 7.33 (dd, *J_1_* = 1.5 Hz, *J_2_* = 8.5 Hz, 2H, Ar-H), 7.10 (br s, 2H, H2_ind_), 6.76 (dd, *J_1_* = 11.0 Hz, *J_2_* = 17.5 Hz, 2H, C*H*=CH_2_), 5.78 (dd, *J_1_* = 1.0 Hz, *J_2_* = 17.5 Hz, 2H, CH=C*H_2_*), 5.26 (dd, *J_1_* = 1.0 Hz, *J_2_* = 11.0 Hz, 2H, CH=C*H_2_*), 4.60 (t, *J* = 7.0 Hz, 1H, C*H*CH_2_NH_2_), 3.51 (d, *J* = 7.0 Hz, 2H, CHC*H_2_*NH_2_) ppm. ^13^C NMR (101 MHz, CDCl_3_): *δ* = 141.8, 137.4, 136.7, 136.1, 135.3, 127.5, 126.7, 126.6, 122.8, 120.0, 119.2, 117.9, 113.7, 109.8, 46.9, 38.5 ppm. HRMS (m/z): [M+H]^+^ calcd for C_34_H_30_N_3_, 480.2434; found 480.2434.

**2,2-bis(6-(3-chlorophenyl)-1*H*-indol-3-yl)ethan-1-amine (3ag):**

Following the general procedure (reaction time = 30 min), compound **3ag** has been purified by flash column chromatography on silica gel (dichloromethane/methanol/ammonia, 99/1/1), obtaining a white solid; yield: 73% (36 mg). *R*_f_ = 0.51 (silica gel; dichloromethane/methanol/ammonia, 90/10/1). ^1^H NMR (400 MHz, DMSO-*d_6_*): *δ* = 10.91 (br s, 2H, NH_ind_), 7.65 (s, 2H, Ar-H), 7.61-7.54 (m, 6H, Ar-H), 7.44 (dd, *J_1_* = *J_2_* = 8.0 Hz, 2H, Ar-H), 7.36-7.31 (m, 4H, Ar-H), 7.21 (dd, *J_1_* = 1.5, *J_2_* = 7.5 Hz, 2H, Ar-H), 4.44 (t, *J* = 7.0 Hz, 1H, C*H*CH_2_NH_2_), 3.31 (d, *J* = 7.0 Hz, 2H, CHC*H_2_*NH_2_), 1.49 (br s, 2H, CHCH_2_N*H_2_*) ppm. ^13^C NMR (101 MHz, DMSO-*d_6_*): *δ* = 144.0, 137.0, 133.5, 131.6, 130.6, 126.8, 126.3, 126.1, 125.3, 123.8, 119.5, 117.5, 116.9, 109.7, 46.7, 38.0 ppm. HRMS (m/z): [M+H]^+^ calcd for C_30_H_24_Cl_2_N_3_, 496.1342; found 496.1339.

**2,2-bis(6-([1,1'-biphenyl]-3-yl)-1*H*-indol-3-yl)ethan-1-amine (3ah):**

Following the general procedure (reaction time = 1h), compound **3ah** has been purified by flash column chromatography on silica gel (dichloromethane/methanol/ammonia, 99/1/1), obtaining a white solid; yield: 90% (52 mg). *R*_f_ = 0.37 (silica gel; dichloromethane/methanol/ammonia, 90/10/1). ^1^H NMR (400 MHz, DMSO-*d_6_*): *δ* = 10.93 (br d, *J* = 2.5 Hz, 2H, NH_ind_), 7.65 (dd, *J_1_* = *J_2_* = 1.5 Hz, 2H, Ar-H), 7.64-7.55 (m, 4H, Ar-H), 7.65 (d, *J* = 1.5 Hz, 2H, Ar-H), 7.64-7.54 (m, 6H, Ar-H), 7.53 (d, *J* = 7.5 Hz, 2H, Ar-H), 7.50-7.45 (m, 4H, Ar-H), 7.41-7.35 (m, 2H, Ar-H), 7.32 (d, *J* = 2.5 Hz, 2H, H2_ind_), 7.29 (dd, *J_1_* = 1.5 Hz, *J_2_* = 8.0 Hz, 2H, Ar-H), 4.47 (t, *J* = 7.0 Hz, 1H, C*H*CH_2_NH_2_), 3.34 (d, *J* = 7.0 Hz, 2H, CHC*H_2_*NH_2_) ppm. ^13^C NMR (101 MHz, DMSO-*d_6_*): *δ* = 142.5, 140.8, 140.4, 137.1, 133.2, 129.4, 128.9, 127.5, 126.9, 126.5, 125.9, 125.1, 124.9, 123.4, 119.5, 117.7, 116.9, 109.6, 46.6, 37.9 ppm. HRMS (m/z): [M+H]^+^ calcd for C_42_H_34_N_3_, 580.2747; found 580.2753.

**2,2-bis(6-(3-(benzyloxy)phenyl)-1*H*-indol-3-yl)ethan-1-amine (3ai):**

Following the general procedure (reaction time = 1h), compound **3ai** has been purified by flash column chromatography on silica gel (dichloromethane/methanol/ammonia, 99/1/1), obtaining a white solid; yield: 80% (51 mg). *R*_f_ = 0.37 (silica gel; dichloromethane/methanol/ammonia, 90/10/1). ^1^H NMR (400 MHz, DMSO-*d_6_*): *δ* = 10.91 (br d, *J* = 2.5 Hz, 2H, NH_ind_), 7.56-7.52 (m, 4H, Ar-H), 7.50-7.45 (m, 4H, Ar-H), 7.42-7.37 (m, 4H, Ar-H), 7.35-7.30 (m, 4H, Ar-H), 7.30 (d, *J* = 2.5 Hz, 2H, H2_ind_), 7.24-7.16 (m, 6H, Ar-H), 6.93 (ddd, *J_1_* = 1.0 Hz, *J_2_* = 2.5 Hz, *J_3_* = 8.0 Hz, 2H, Ar-H), 5.17 (s, 4H, OC*H_2_*Ph), 4.42 (t, 7.0 Hz, 1H, C*H*CH_2_NH_2_), 3.30 (d, *J* = 7.0 Hz, 2H, CHC*H_2_*NH_2_), 1.48 (br s, 2H, CHCH_2_N*H_2_*) ppm. ^13^C NMR (101 MHz, DMSO-*d_6_*): *δ* = 158.8, 143.3, 137.2, 137.0, 133.1, 129.8, 128.4, 127.8, 127.7, 126.5, 123.3, 119.4, 119.3, 117.6, 116.9, 113.1, 112.9, 109.5, 69.2, 46.7, 38.1 ppm. HRMS (m/z): [M+H]^+^ calcd for C_44_H_38_N_3_O_2_, 640.2959; found 640.2955.

**2,2-bis(6-(3-methoxyphenyl)-1*H*-indol-3-yl)ethan-1-amine (3aj):**

Following the general procedure (reaction time = 2h), compound **3aj** has been purified by flash column chromatography on silica gel (Chloroform/methanol/ammonia, 99/1/1), obtaining a white solid; yield: 82% (40 mg). *R*_f_ = 0.36 (silica gel; Chloroform/methanol/ammonia, 90/10/1). ^1^H NMR (400 MHz, DMSO-*d_6_*): *δ* = 10.91 (br d, *J* = 2.5 Hz, 2H, NH_ind_), 7.56-7.51 (m, 4H, Ar-H), 7.33 (dd, *J_1_* = *J_2_* = 8.0 Hz, 2H, Ar-H), 7.30 (d, *J* = 2.5 Hz, 2H, H2_ind_), 7.21-7.16 (m, 4H, Ar-H), 7.13 (dd, *J_1_* = *J_2_* = 2.0 Hz, 2H, Ar-H), 6.86 (ddd, *J_1_* = 1.0 Hz, *J_2_* = 2.5 Hz, *J_3_* = 8.0 Hz, 2H, Ar-H), 4.42 (t, *J* = 7.0 Hz, 1H, C*H*CH_2_NH_2_), 3.80 (s, 6H, OC*H_3_*), 3.30 (d, *J* = 7.0 Hz, 2H, CHC*H_2_*NH_2_) ppm. ^13^C NMR (101 MHz, DMSO-*d_6_*): *δ* = 159.6, 143.3, 137.0, 133.2, 129.8, 126.5, 123.3, 119.4, 119.1, 117.6, 116.9, 112.2, 111.9, 109.5, 55.0, 46.6, 38.0 ppm. HRMS (m/z): [M+H]^+^ calcd for C_32_H_30_N_3_O_2_, 488.2333; found 488.2340.

**2,2-bis(6-(2-methoxyphenyl)-1*H*-indol-3-yl)ethan-1-amine (3ak):**

Following the general procedure (reaction time = 2h), compound **3ak** has been purified by flash column chromatography on silica gel (dichloromethane/methanol/ammonia, 99/1/1), obtaining a white solid; yield: 76% (37 mg). *R_f_* = 0.38 (silica gel; dichloromethane/methanol/ammonia, 90/10/1). ^1^H NMR (400 MHz, DMSO-*d_6_*): *δ* = 10.82 (br d, *J* = 2.5 Hz, 2H, NH_ind_), 7.50 (d, *J* = 8.5 Hz, 2H, Ar-H), 7.41 (dd, *J_1_* = 0.5 Hz, *J_2_* = 1.5 Hz, 2H, Ar-H), 7.30-7.25 (m, 6H, Ar-H), 7.07 (d, *J* = 8.0 Hz, 2H, Ar-H), 7.02-6.96 (m, 4H, Ar-H), 4.41 (t, *J* = 7.0 Hz, 1H, C*H*CH_2_NH_2_), 3.72 (s, 6H, OC*H_3_*), 3.30 (d, *J* = 7.0 Hz, 2H, CHC*H_2_*NH_2_) ppm. ^13^C NMR (101 MHz, DMSO-*d_6_*): *δ* = 156.2, 136.4, 131.3, 130.8, 130.6, 127.9, 125.8, 122.7, 120.7, 120.0, 118.3, 116.8, 112.1, 111.7, 55.4, 46.6, 38.1 ppm. HRMS (m/z): [M+H]^+^ calcd for C_32_H_30_N_3_O_2_, 488.2333; found 488.2333.

**2,2-bis(6-(2,6-dimethoxyphenyl)-1*H*-indol-3-yl)ethan-1-amine (3al):**

Following the general procedure (reaction time = 20 min), compound **3al** has been purified by flash column chromatography on silica gel (dichloromethane/methanol/ammonia, 99/1/1), obtaining a white solid; yield: 58% (32 mg). *R_f_* = 0.44 silica gel; (dichloromethane/methanol/ammonia, 90/10/1). ^1^H NMR (400 MHz, DMSO-*d_6_*): *δ* = 10.70 (br d, *J* = 2.5 Hz, 2H, NH_ind_), 7.49 (d, *J* = 8.0 Hz, 2H, Ar-H), 7.26 (dd, *J_1_* = *J_2_* = 8.5 Hz, 2H, Ar-H), 7.24 (s, 2H, Ar-H), 7.11 (dd, *J*_1_ = 0.5 Hz, *J*_2_ = 1.5 Hz, 2H, Ar-H), 6.73 (dd, *J_1_* = 1.5 Hz, *J_2_* = 8.0 Hz, 2H, Ar-H), 6.72-6.69 (m, 4H, Ar-H), 4.41 (t, *J* = 7,0 Hz, 1H, C*H*CH_2_NH_2_), 3.62 (s, 12H, OC*H_3_*), 3.31 (d, *J* = 7.0 Hz, 2H, CHC*H_2_*NH_2_) ppm. ^13^C NMR (101 MHz, DMSO-*d_6_*): *δ* = 157.4, 136.3, 128.2, 126.5, 125.5, 122.1, 121.3, 120.1, 117.8, 116.8, 113.4, 104.3, 55.5, 46.6, 38.0 ppm. HRMS (m/z): [M+H]^+^ calcd for C_34_H_34_N_3_O_4_, 548.2544; found 548.2564.

**2,2-bis(6-(2,6-dimethylphenyl)-1*H*-indol-3-yl)ethan-1-amine (3am):**

Following the general procedure (reaction time = 4h), compound **3am** has been purified by flash column chromatography on silica gel (dichloromethane/ammonia, 99/1 to dichloromethane/methanol/ammonia, 99/1/1), obtaining an off white solid; yield: 23% (11 mg). *R*_f_ = 0.56 (silica gel; dichloromethane/methanol/ammonia, 90/10/1). ^1^H NMR (400 MHz, DMSO-*d_6_*): *δ* = 10.83 (br s, 2H, NH_ind_), 7.66 (d, *J* = 8.0 Hz, 2H, Ar-H), 7.31 (d, *J* = 2.5 Hz, 2H, Ar-H), 7.14-7.07 (m, 6H, Ar-H), 7.05 (s, 2H, Ar-H), 6.67 (dd, *J_1_* = 1.5 Hz, *J_2_* = 8.0 Hz, 2H, Ar-H), 4.47 (t, *J* = 7.5 Hz, 1H, C*H*CH_2_NH_2_), 3.34 (d, *J* = 7.5 Hz, 2H, CHC*H_2_*NH_2_), 1.96 (s, 6 H, CH_3_), 1.95 (s, 6H, CH_3_) ppm. ^13^C NMR (101 MHz, DMSO-*d_6_*): *δ* = 142.6, 136.7, 135.6, 133.0, 127.1, 126.5, 125.6, 122.4, 119.2, 119.1, 117.0, 111.2, 46.8, 38.0, 20.8, 20.7 ppm. HRMS (m/z): [M+H]^+^ calcd for C_34_H_34_N_3_, 484.2747; found 484.2750.

**2,2-bis(6-(naphthalen-1-yl)-1*H*-indol-3-yl)ethan-1-amine (3an):**

Following the general procedure (reaction time = 15 min), compound **3an** has been purified by flash column chromatography on silica gel (dichloromethane/methanol/ammonia, 99/1/1), obtaining an off white solid; yield: 93% (49 mg). *R*_f_ = 0.35 (silica gel; dichloromethane /methanol/ammonia, 90/10/1). ^1^H NMR (400 MHz, CDCl_3_): *δ* = 8.19 (br s, 2H, NH_ind_), 8.00 (d, *J* = 8.5 Hz, 2H, Ar-H), 7.91 (d, *J* = 8.0 Hz, 2H, Ar-H), 7.85 (d, *J* = 8.0 Hz, 2H, Ar-H), 7.75 (d, *J* = 8.0 Hz, 2H, Ar-H), 7.54-7.44 (m, 8H, Ar-H), 7.38 (dd, *J_1_* = *J_2_* = 8.0 Hz, 2H, Ar-H), 7.23 (dd, *J_1_* = 1.5 Hz, *J_2_* = 8.0 Hz, 2H, Ar-H), 7.15 (d, *J* = 2.0 Hz, 2H, Ar-H), 4.69 (t, *J* = 7.0 Hz, 1H, C*H*CH_2_NH_2_), 3.58 (d, *J* = 7.0 Hz, 2H, CHC*H_2_*NH_2_) ppm. ^13^C NMR (101 MHz, CDCl_3_): *δ* = 141.3, 136.9, 134.9, 134.0, 132.2, 128.3, 127.3 (2C), 126.6, 126.4, 126.0, 125.8, 125.5, 122.7, 122.2, 119.3, 117.8, 112.8, 46.9, 38.6 ppm. HRMS (m/z): [M+H]^+^ calcd for C_38_H_30_N_3_, 528.2434; found 528.2445.

**2,2-bis(6-(naphthalen-2-yl)-1*H*-indol-3-yl)ethan-1-amine (3ao):**

Following the general procedure (reaction time = 2h), compound **3ao** has been purified by flash column chromatography on silica gel (dichloromethane/methanol/ammonia, 99/1/1), obtaining a white solid; yield: 75% (40 mg). *R*_f_ = 0.29 (silica gel; dichloromethane/methanol/ammonia, 90/10/1). ^1^H NMR (400 MHz, DMSO-*d_6_*): *δ* = 10.99 (br d, *J* = 2.5 Hz, 2H, NH_ind_), 8.15 (d, *J* = 2.0 Hz, 2H, Ar-H), 7.99-7.95 (m, 4H, Ar-H), 7.91 (d, *J* = 8.0 Hz, 2H, Ar-H), 7.84 (dd, *J_1_* = 2.0 Hz, *J_2_* = 8.5 Hz, 2H, Ar-H), 7.72 (d, *J* = 1.5 Hz, 2H, Ar-H), 7.63 (d, *J* = 8.5 Hz, 2H, Ar-H), 7.54-7.45 (m, 4H, Ar-H), 7.38 (dd, *J_1_* = 1.5 Hz, *J_2_* = 8.5 Hz, 2H, Ar-H), 7.35 (d, *J* = 2.5 Hz, 2H, H2_ind_), 4.51 (t, *J* = 7.0 Hz, 1H, C*H*CH_2_NH_2_), 3.33 (d, *J* = 7.0 Hz, 2H, CHC*H_2_*NH_2_) ppm. ^13^C NMR (101 MHz, DMSO-*d_6_*): *δ* = 139.6, 137.7, 134.0, 133.5, 132.3, 128.7, 128.5, 127.9, 127.0, 126.7, 126.1 (2C), 125.1, 124.0, 120.0, 118.3, 117.2, 110.3, 47.0, 38.1 ppm. HRMS (m/z): [M+H]^+^ calcd for C_38_H_30_N_3_, 528.2434; found 528.2442.

**di-*tert*-butyl 2,2'-((2-aminoethane-1,1-diyl)bis(1*H*-indole-3,6-diyl))bis(1*H*-pyrrole-1-carboxylate) (3ap)**

Following the general procedure (reaction time = 15 min), compound **3ap** has been purified by flash column chromatography on silica gel (Chloroform/methanol/ammonia, 99/1/1), obtaining a white solid; yield: 91% (55 mg). *R*_f_ = 0.42 (silica gel; Chloroform/methanol/ammonia, 90/10/1). ^1^H NMR (400 MHz, CDCl_3_): *δ* = 8.07 (br s, 2H, NH_ind_), 7.49 (d, *J* = 8.5 Hz, 2H, H4_ind_), 7.35 (dd, *J_1_* = 0.5 Hz, *J_2_* = 1.5 Hz, 2H, H7_ind_), 7.33 (dd, *J_1_* = 2.0 Hz, *J_2_* = 3.5 Hz, 2H, H4_pyr_), 7.03 (dd, *J_1_* = 1.5 Hz, *J_2_* = 8.5 Hz, 2H, H5_ind_), 6.85 (d, *J* = 2.0 Hz, 2H, H2_ind_), 6.22 (dd, *J*_1_ = *J*_2_ = 3.5 Hz, 2H, H3_pyr_), 6.17 (dd, *J*_1_ = 2.0, *J*_2_ = 3.5 Hz, 2H, H2_pyr_), 4.50 (t, *J* = 7.0 Hz, 1H, C*H*CH_2_NH_2_), 3.41 (d, *J* = 7.0 Hz, 2H, CHC*H_2_*NH_2_), 1.32 (s, 18H, Boc) ppm.^13^C NMR (101 MHz, CDCl_3_): *δ* = 149.6, 136.4, 136.3, 128.2, 126.4, 122.6, 122.3, 121.5, 118.6, 117.7, 114.3, 111.8, 110.6, 83.5, 46.9, 38.4, 27.8 ppm. HRMS (m/z): [M+H]^+^ calcd for C_36_H_40_N_5_O_4_, 606.3075; found 606.3087.

**2,2-di(1*H*,1'*H*-[4,6'-biindol]-3'-yl)ethan-1-amine (3aq):**

Following the general procedure (reaction time = 4h), compound **3aq** has been purified by flash column chromatography on silica gel (dichloromethane/methanol/ammonia, 99/1/1), obtaining a white solid; yield: 55% (28 mg). *R*_f_ = 0.37 (silica gel; dichloromethane/methanol/ammonia, 90/10/1). ^1^H NMR (400 MHz, DMSO-*d_6_*): *δ* = 11.18 (br s, 2H, NH_ind_), 10.88 (br d, *J* = 2,5 Hz, 2H, NH_ind_), 7.65 (d, *J* = 8.5 Hz, 2H, Ar-H), 7.62 (d, *J* = 1.5 Hz, 2H, Ar-H), 7.37 (dd, *J*_1_ = *J*_2_ = 3.0 Hz, 2H, Ar-H), 7.35 (ddd, *J*_1_ = *J*_2_ = 1.5 Hz, *J*_3_ = 8.0 Hz, 2H, Ar-H), 7.32 (d, *J* = 2,5 Hz, 2H, Ar-H), 7.25 (dd, *J_1_* = 1.5 Hz, *J_2_* = 8.0 Hz, 2H, Ar-H), 7.15 (dd, *J_1_* = *J_2_* = 7.5 Hz, 2H, Ar-H), 7.06 (dd, *J_1_* = 1.0 Hz, *J_2_* = 7.5 Hz, 2H, Ar-H), 6.60-6.57 (m, 2H, Ar-H), 4.48 (t, *J* = 7.0 Hz, 1H, C*H*CH_2_NH_2_), 3.35 (d, *J* = 7.0 Hz, 2H, CHC*H_2_*NH_2_) ppm. ^13^C NMR (101 MHz, DMSO-*d_6_*): *δ* = 137.0, 136.4, 134.6, 134.0, 126.0, 125.8, 125.4, 122.9, 121.3, 119.1, 119.0, 118.5, 116.9, 110.9, 109.9, 100.5, 46.7, 38.1 ppm. HRMS (m/z): [M+H]^+^ calcd for C_34_H_28_N_5_, 506.2339; found 506.2347.

**2,2-bis(6-((E)-4-chlorostyryl)-1*H*-indol-3-yl)ethan-1-amine (3ar):**

Following the general procedure (reaction time = 25 min), compound **3ar** has been purified by flash column chromatography on silica gel (dichloromethane/methanol/ammonia, 99/1/1), obtaining a yellow solid; yield: 78% (43 mg). *R*_f_ = 0.29 (silica gel; dichloromethane/methanol/ammonia, 90/10/1). ^1^H NMR (600 MHz, DMSO-*d_6_*): *δ* = 10.96 (br d, *J* = 2.5 Hz, 2H, NH_ind_), 7.62-7.59 (m, 4H, Ar-H), 7.51-7.48 (m, 4H, Ar-H), 7.41-7.38 (m, 4H, Ar-H), 7.34 (d, *J* = 16.5 Hz, 2H, CH=CH), 7.28 (d, *J* = 2.5 Hz, 2H, H2_ind_), 7.24 (dd, *J_1_* = 1.5 Hz, *J_2_* = 8.5 Hz, 2H, Ar-H), 7.12 (d, *J* = 16.5 Hz, 2H, CH=CH), 4.43 (t, *J* = 7.0 Hz, 1H, C*H*CH_2_NH_2_), 3.29 (d, *J* = 7.0 Hz, 2H, CHC*H_2_*NH_2_) ppm. ^13^C NMR (151 MHz, DMSO-*d_6_*): *δ* = 136.8, 136.4, 131.1, 131.0, 129.8, 128.9, 127.7, 127.0, 124.1, 123.7, 119.1, 117.1, 116.8, 110.5, 46.4, 37.3 ppm. HRMS (m/z): [M+H]^+^ calcd for C_34_H_28_Cl_2_N_3_, 548.1655; found 548.1652.

**2,2-bis(5-phenyl-1*H*-indol-3-yl)ethan-1-amine (3ba):**

Following the general procedure (reaction time = 2h), compound **3ba** has been purified by flash column chromatography on silica gel (dichloromethane/methanol/ammonia, 99/1/1), obtaining a white solid; yield: 70% (30 mg). *R_f_* = 0.37 (silica gel; dichloromethane/methanol/ammonia, 90/10/1). ^1^H NMR (400 MHz, CDCl_3_): *δ* = 8.10 (br s, 2H, NH_ind_), 7.83 (s, 2H, H4_ind_), 7.60-7.55 (m, 4H, Ar-H), 7.43-7.36 (m, 8H, Ar-H), 7.29 (d, *J* = 7.5 Hz, 2H, Ar-H), 7.06 (br s, 2H, H2_ind_), 4.66 (t, *J* = 7.0 Hz, 1H, C*H*CH_2_NH_2_), 3.51 (d, *J* = 7.0 Hz, CHC*H_2_*NH_2_) ppm. ^13^C NMR (101 MHz, CDCl_3_): *δ* = 142.6, 136.3, 133.1, 128.8, 127.6, 127.5, 126.5, 122.9, 122.1, 118.2, 117.9, 111.6, 46.5, 38.1 ppm. HRMS (m/z): [M+H]^+^ calcd for C_30_H_26_N_3_, 428.2121; found 428.2127.

**2,2-bis(7-phenyl-1*H*-indol-3-yl)ethan-1-amine (3ca):**

Following the general procedure (reaction time = 1.5h), compound **3ca** has been purified by flash column chromatography on silica gel (dichloromethane/methanol/ammonia, 99/1/1), obtaining an off white solid; yield: 89% (38 mg). *R_f_* = 0.39 (silica gel; dichloromethane/methanol/ammonia, 90/10/1). ^1^H NMR (400 MHz, CDCl_3_): *δ* = 8.27 (br s, 2H, NH_ind_), 7.64-7.59 (m, 6H, Ar-H), 7.51-7.46 (m, 4H, Ar-H), 7.41-7.36 (m, 2H, Ar-H), 7.19 (dd, *J_1_* = 1.5 Hz, *J_2_* = 7.5 Hz, 2H, H4_ind_), 7.14 (dd, *J_1_* = *J_2_* = 7.5 Hz, 2H, H5_ind_) 7.06 (d, *J* = 2.5 Hz, 2H, H2_ind_), 4.64 (t, *J* = 7.0 Hz, 1H, C*H*CH_2_NH_2_), 3.50 (d, *J* = 7.0 Hz, 2H, CHC*H_2_*NH_2_) ppm. ^13^C NMR (101 MHz, CDCl_3_): *δ* = 139.3, 134.6, 129.3, 128.4, 127.6, 127.5, 125.8, 122.3, 122.1, 120.0, 119.0, 118.3, 46.9, 38.6 ppm. HRMS (m/z): [M+H]^+^ calcd for C_30_H_26_N_3_, 428.2121; found 428.2121.

**2-(6-phenyl-1*H*-indol-3-yl)ethan-1-amine (3ea).**

Following the general procedure (reaction time = 2h), compound **3ea** has been purified by flash column chromatography on silica gel (dichloromethane/methanol/ammonia, 99/1/1), obtaining a white solid; yield: 51% (12 mg). *R_f_* = 0.30 (silica gel; dichloromethane/methanol/ammonia, 90/10/1). ^1^H NMR (400 MHz, DMSO-*d_6_*): *δ* = 10.89 (br s, 1H, NH_ind_), 7.67-7.63 (m, 2H, Ar-H), 7.59 (d, *J* = 8.5 Hz, 1H, H4_ind_), 7.57 (d, *J* = 1.5 Hz, 1H, H7_ind_), 7.47-7.41 (m, 2H, Ar-H), 7.33-7.28 (m, 1H), 7.28 (dd, *J_1_* = 8.0 Hz, *J_2_* = 1.5 Hz, 1H, H5_ind_), 7.18 (d, J = 2.0 Hz, 1H, H2_ind_), 2.87-2.80 (m, 2H, C*H_2_*-C*H_2_*-NH_2_), 2.80-2.72 (m, 2H, C*H_2_*-C*H_2_*-NH_2_) ppm. ^13^C NMR (101 MHz, DMSO-*d_6_*): *δ* = 141.8, 136.9, 133.3, 128.8, 126.9, 126.7, 126.4, 123.6, 118.8, 117.6, 112.6, 109.4, 42.7, 29.5 ppm. HRMS (m/z): [M+H]^+^ calcd for C_16_H_17_N_2_, 237.1386; found 237.1385.

**2-(6-bromo-1*H*-indol-3-yl)-2-(6-(2,6-dimethylphenyl)-1*H*-indol-3-yl)ethan-1-amine (4am).**

Following the general procedure (reaction time = 4h), compound **4am** has been purified by flash column chromatography on silica gel (dichloromethane/ammonia, 99/1 to dichloromethane/methanol/ammonia, 99/1/1), obtaining a white solid; yield: 27% (13 mg). *R_f_* = 0.44 (silica gel; dichloromethane/methanol/ammonia, 90/10/1). ^1^H NMR (400 MHz, DMSO-*d_6_*): *δ* = 11.00 (br s, 1H, NH_ind_), 10.81 (br s, 1H, NH_ind_), 7.55 (d, *J* = 8.0 Hz, 1H, H4_ind_), 7.53 (d, *J* = 8.5 Hz, 1H, H4_ind_), 7.51 (d, *J* = 2.0 Hz, 1H, H2_ind_), 7.32 (d, *J* = 2.0 Hz, 1H, H7_ind_), 7.24 (d, *J* = 2.5 Hz, 1H, H7_ind_), 7.13-7.02 (m, 5H, Ar-H), 6.63 (dd, *J_1_* = 1.5 Hz, *J_2_* = 8.0 Hz, 1H, H5_ind_), 4.41 (t, *J* = 7.0 Hz, 1H, C*H*CH_2_NH_2_), 3.28 (d, *J* = 7.0 Hz, 2H, CHC*H_2_*NH_2_), 1.95 (s, 3H, C*H_3_*), 1.94 (s, 3H, C*H_3_*) ppm. ^13^C NMR (101 MHz, DMSO-*d_6_*): *δ* = 142.6, 137.3, 136.7, 135.7, 135.6, 133.1, 127.1, 126.5, 126.0, 125.5, 123.5, 122.4, 120.9, 120.7, 119.0, 117.3, 116.7, 113.9, 113.6, 111.2, 46.7, 37.8, 20.79, 20.76 ppm. HRMS (m/z): [M+H]^+^ calcd for C_26_H_25_BrN_3_, 458.1226; found 458.1233.

1. **^1^H NMR and ^13^C NMR Spectra**


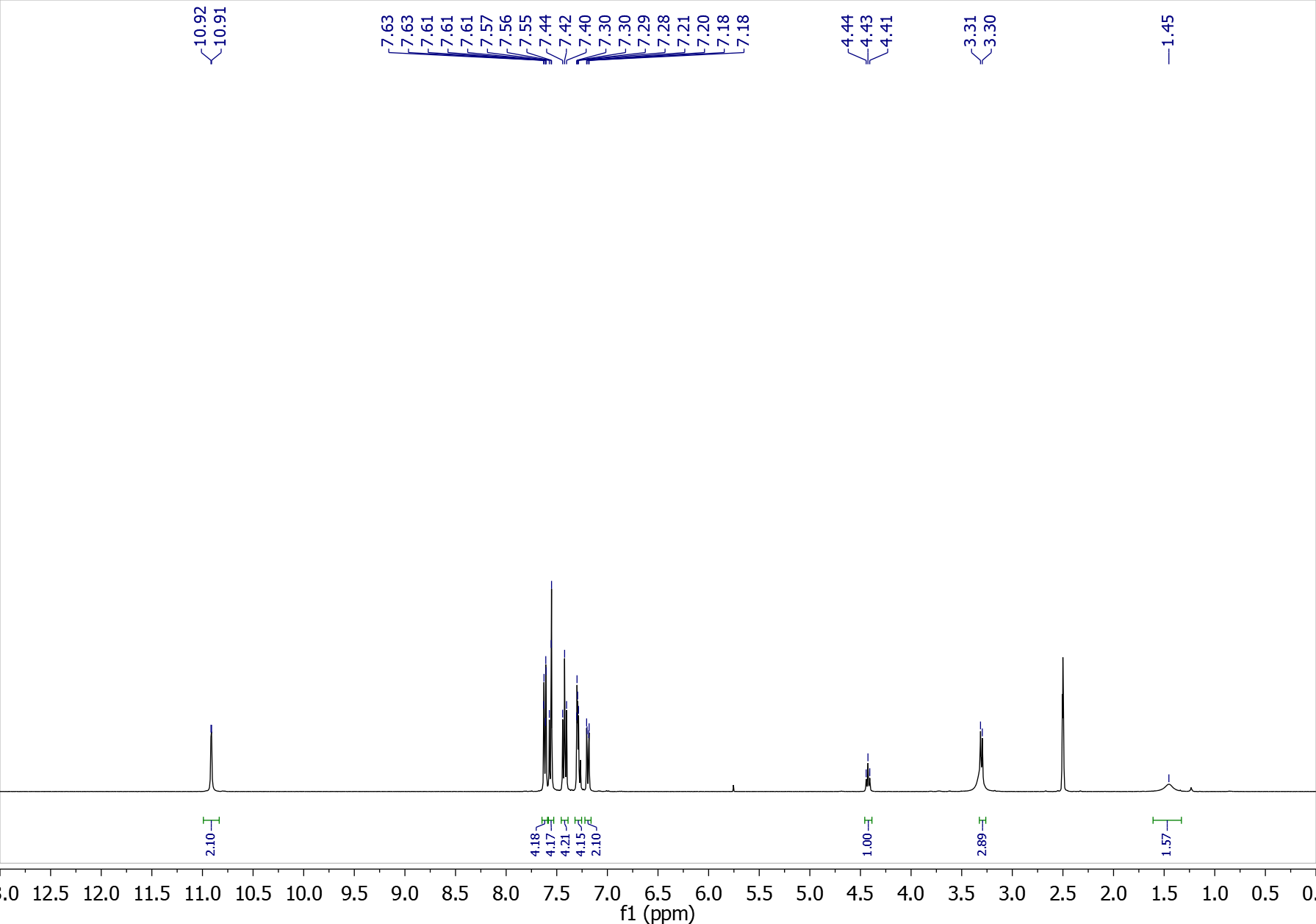

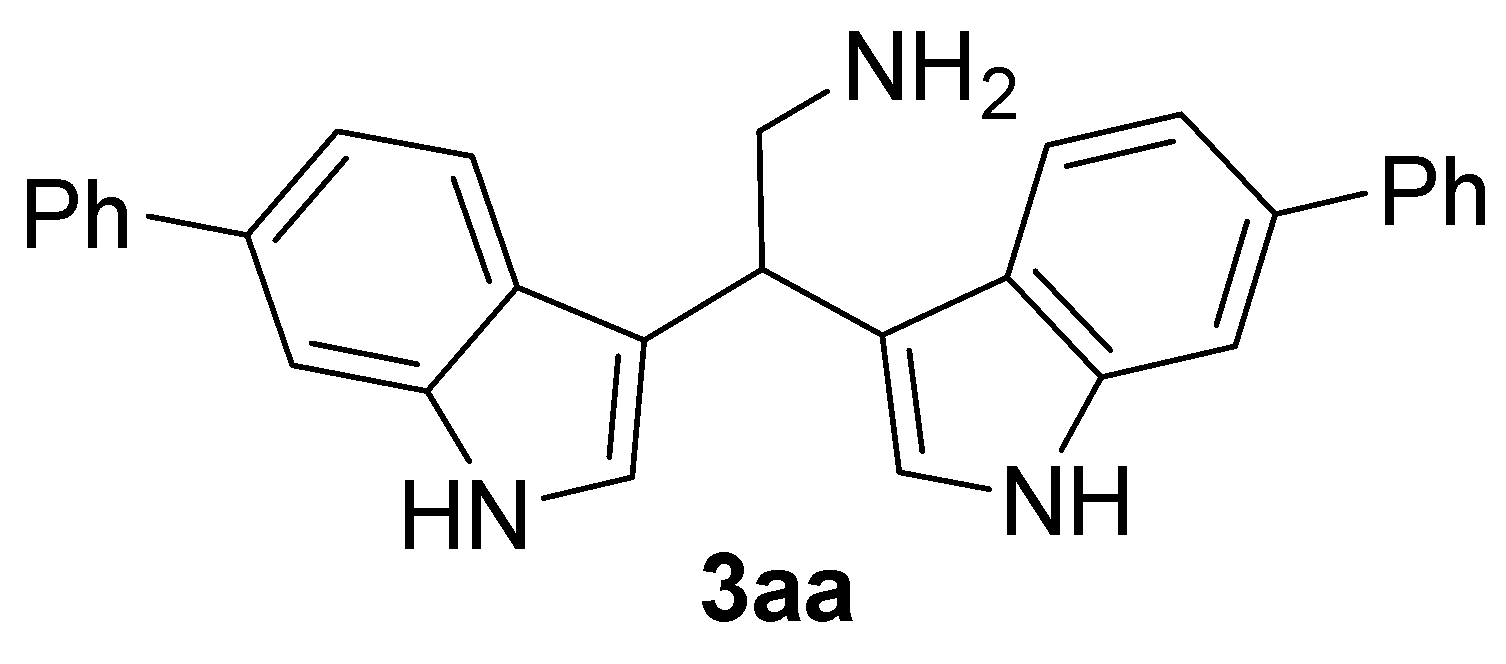


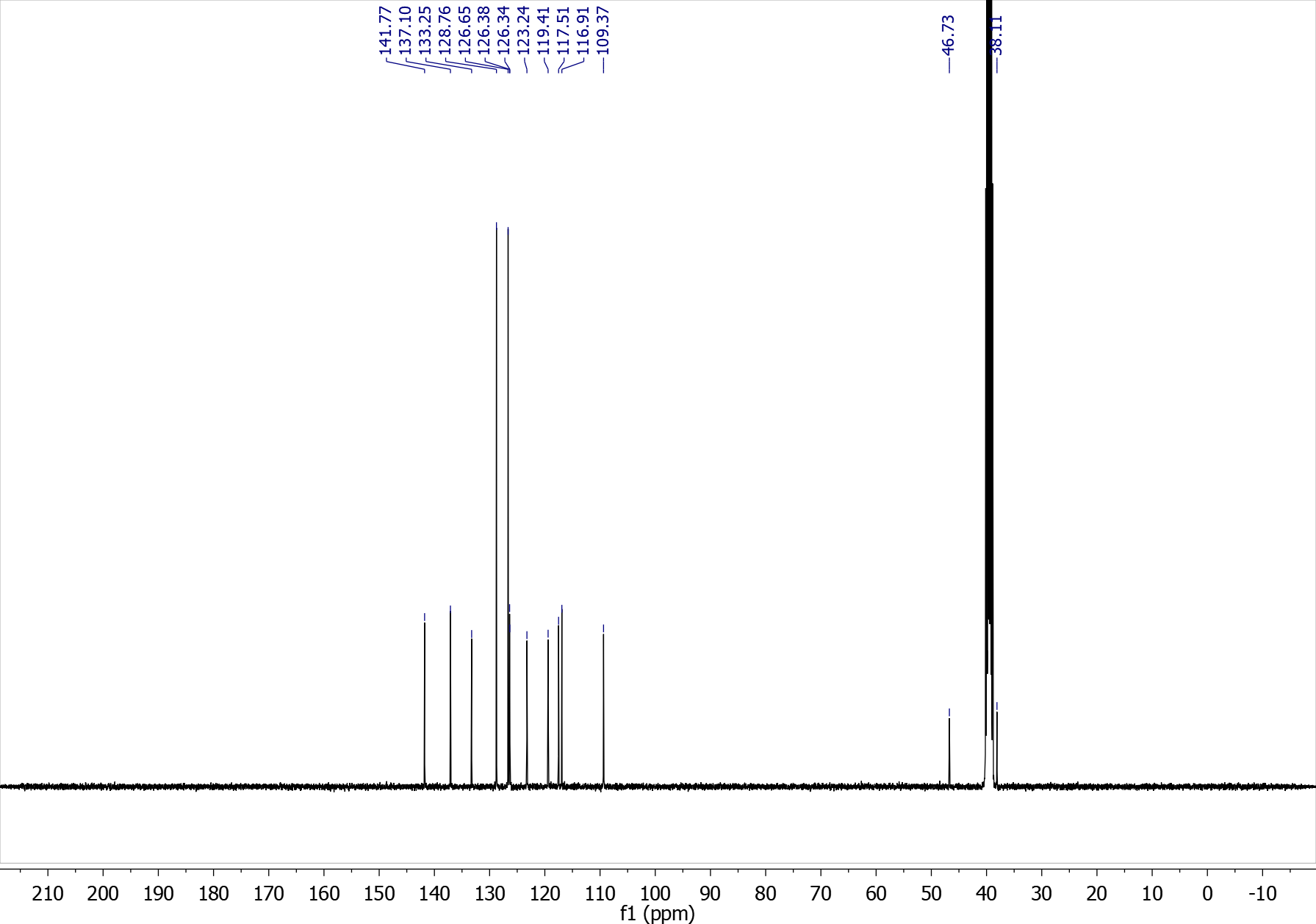


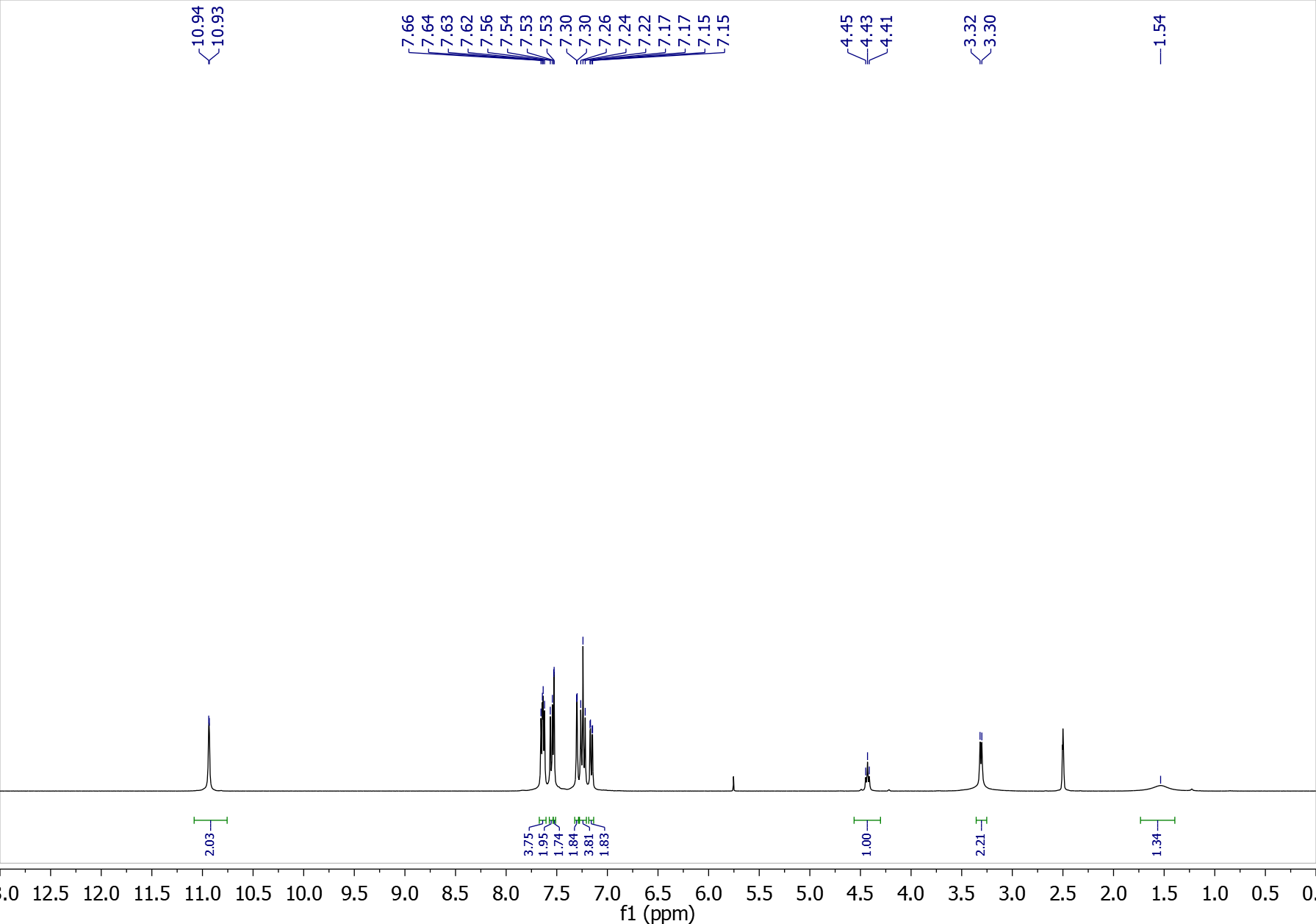

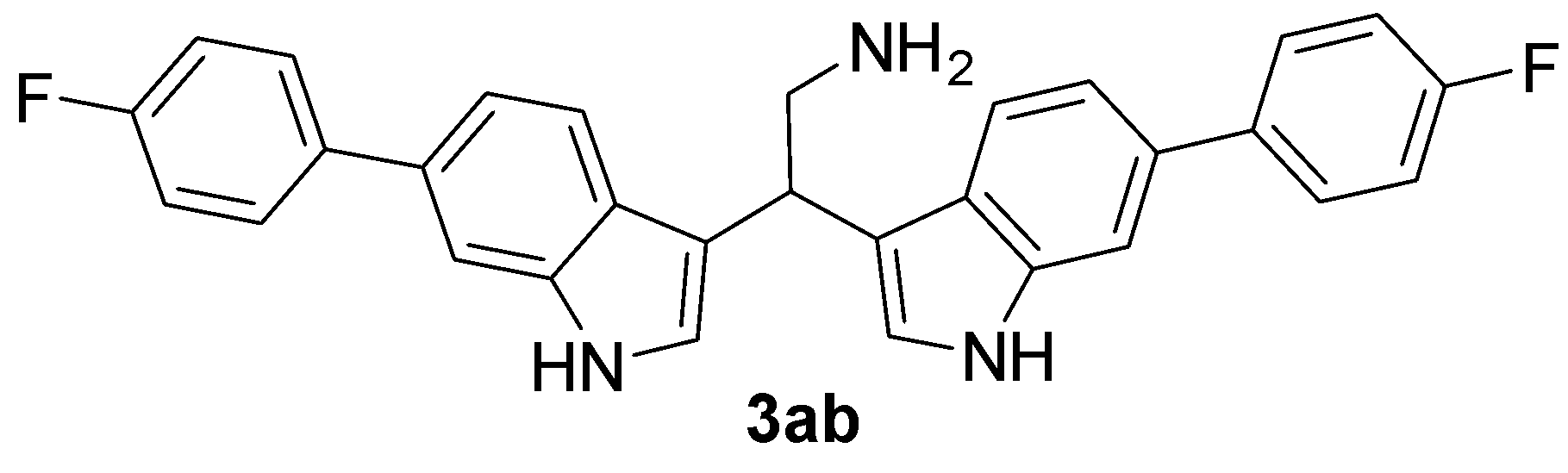


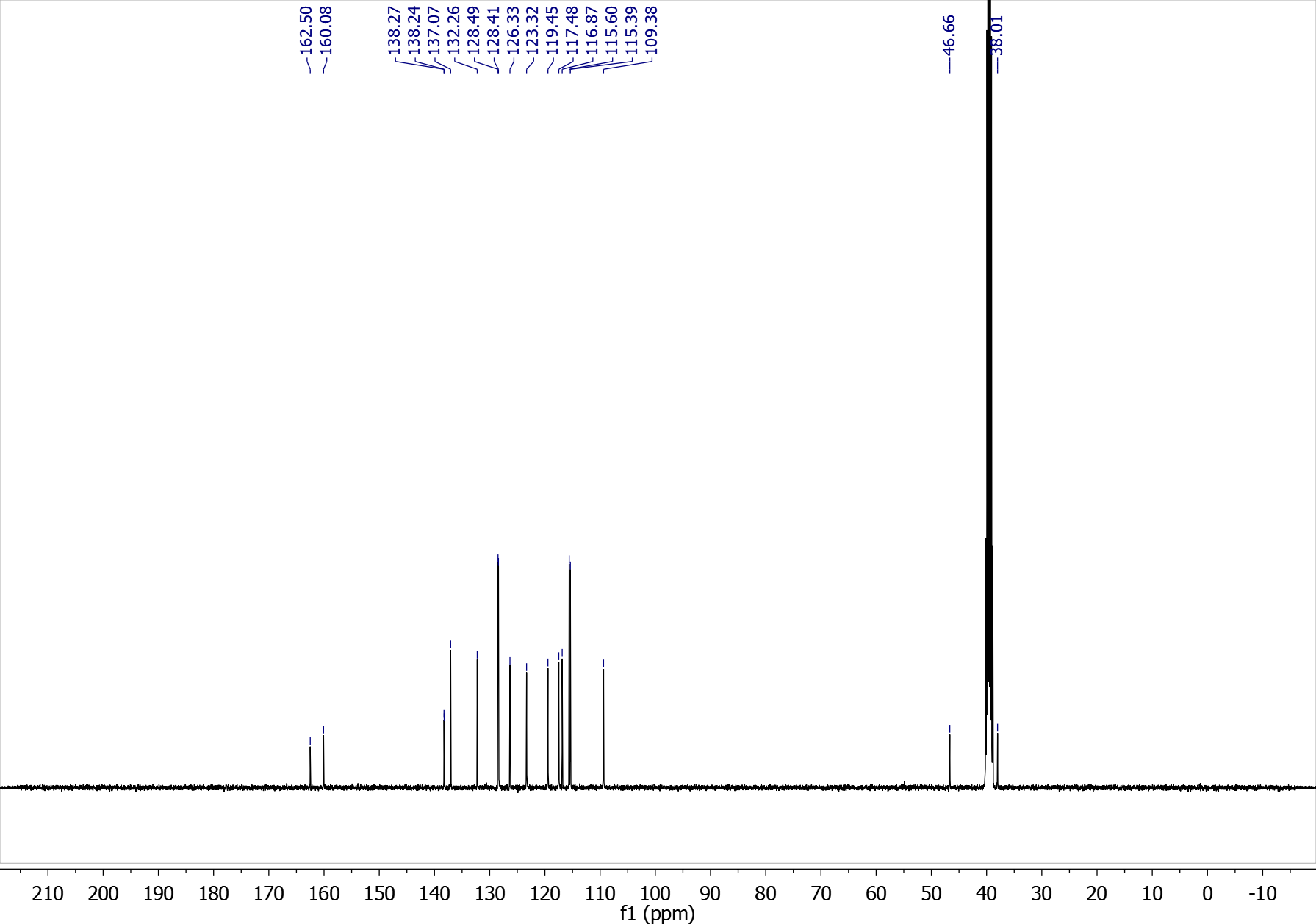


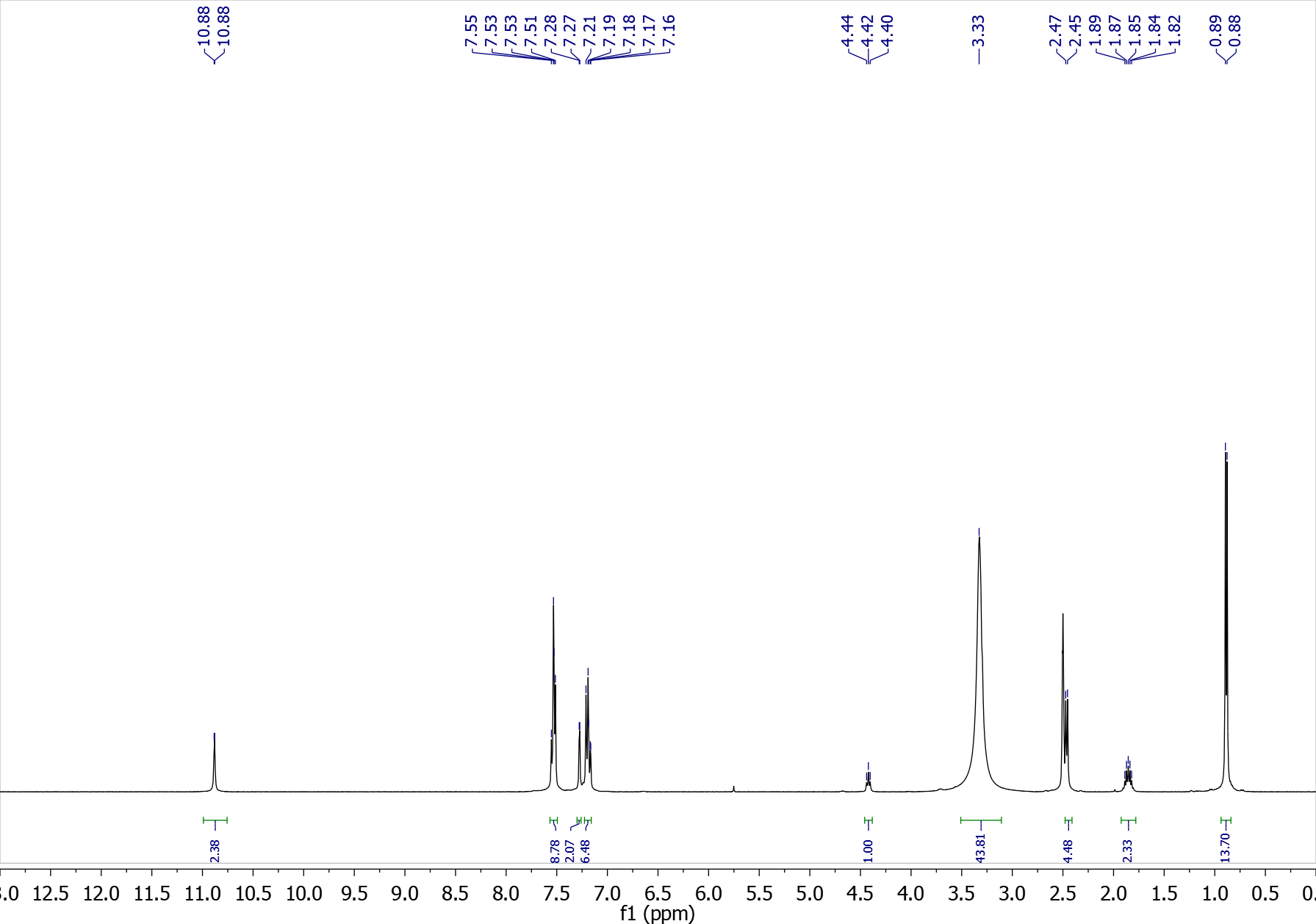

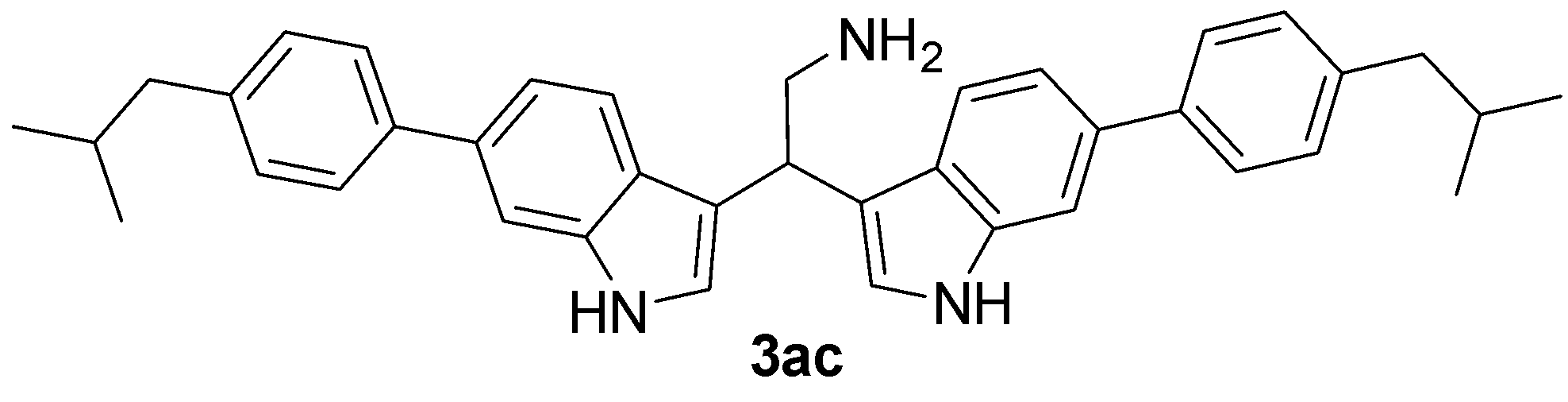


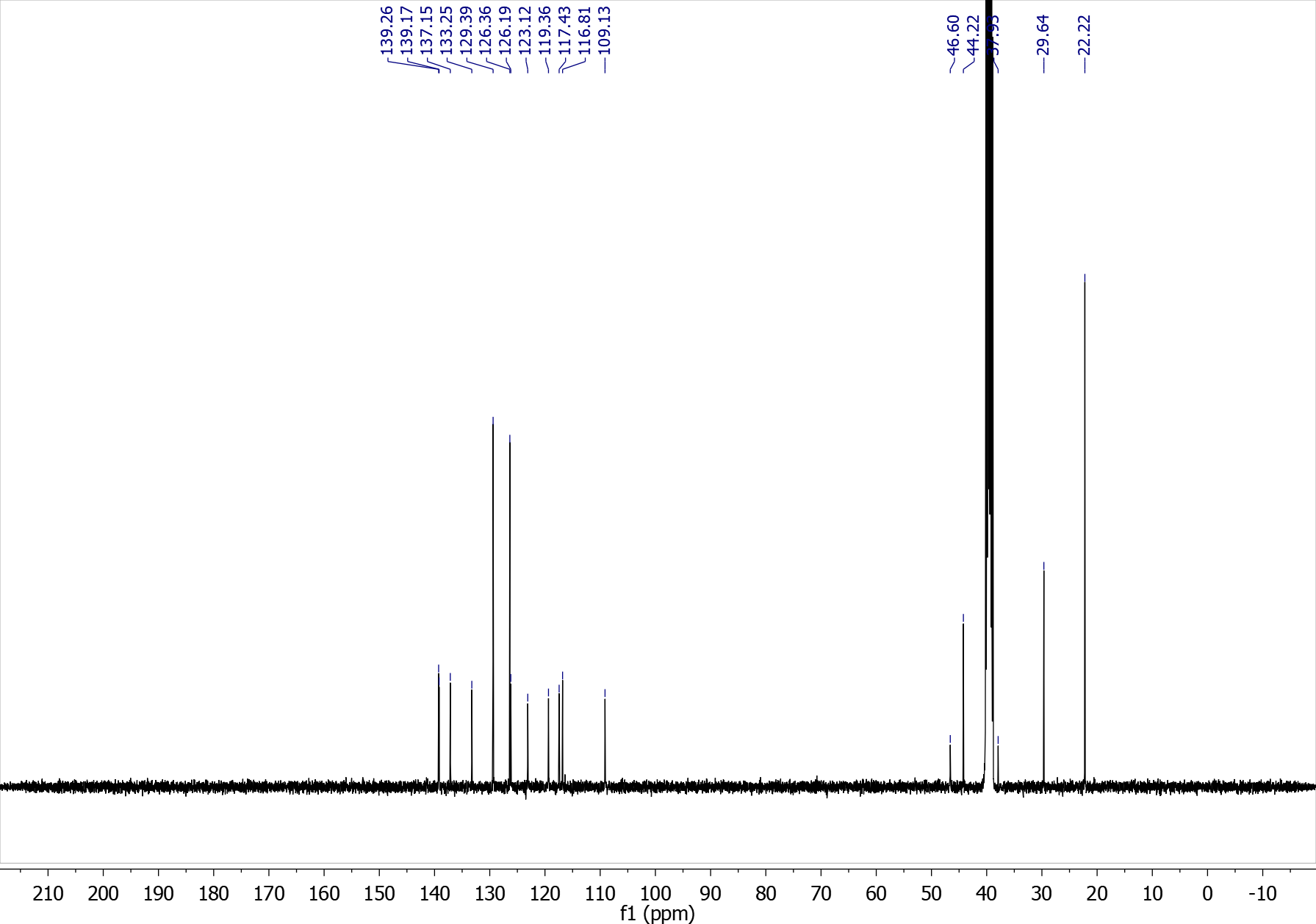


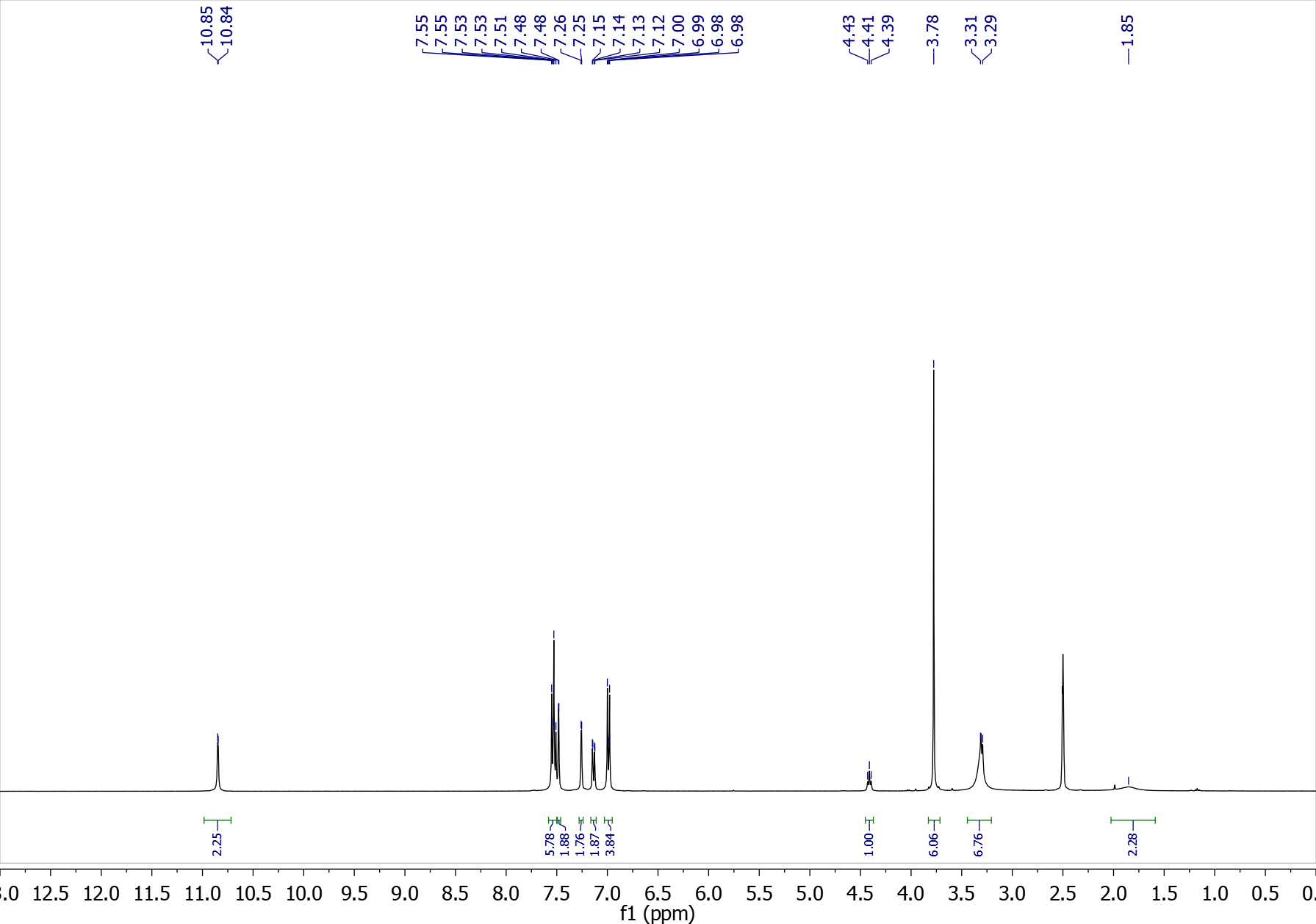

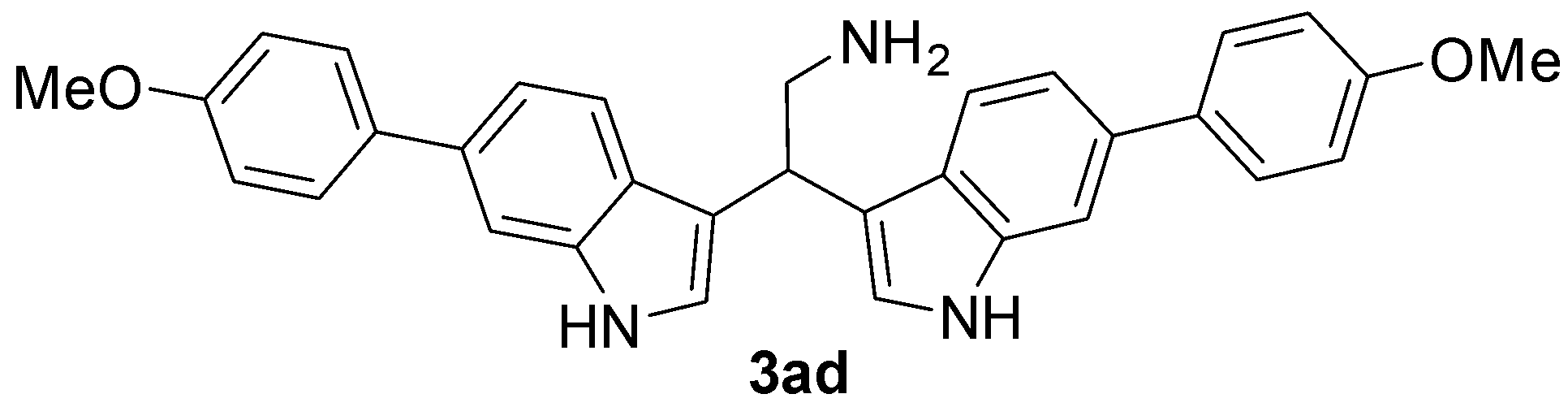


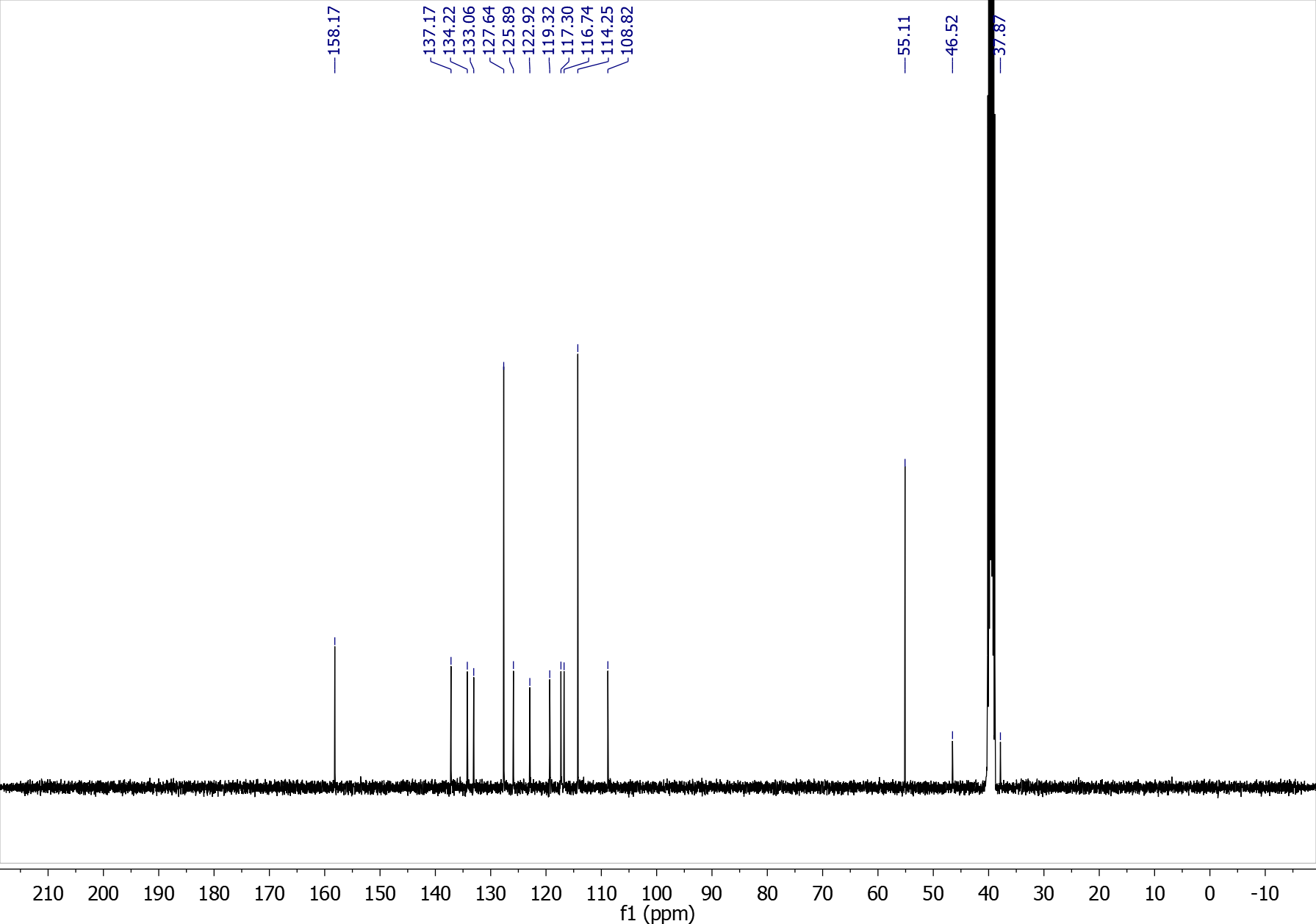


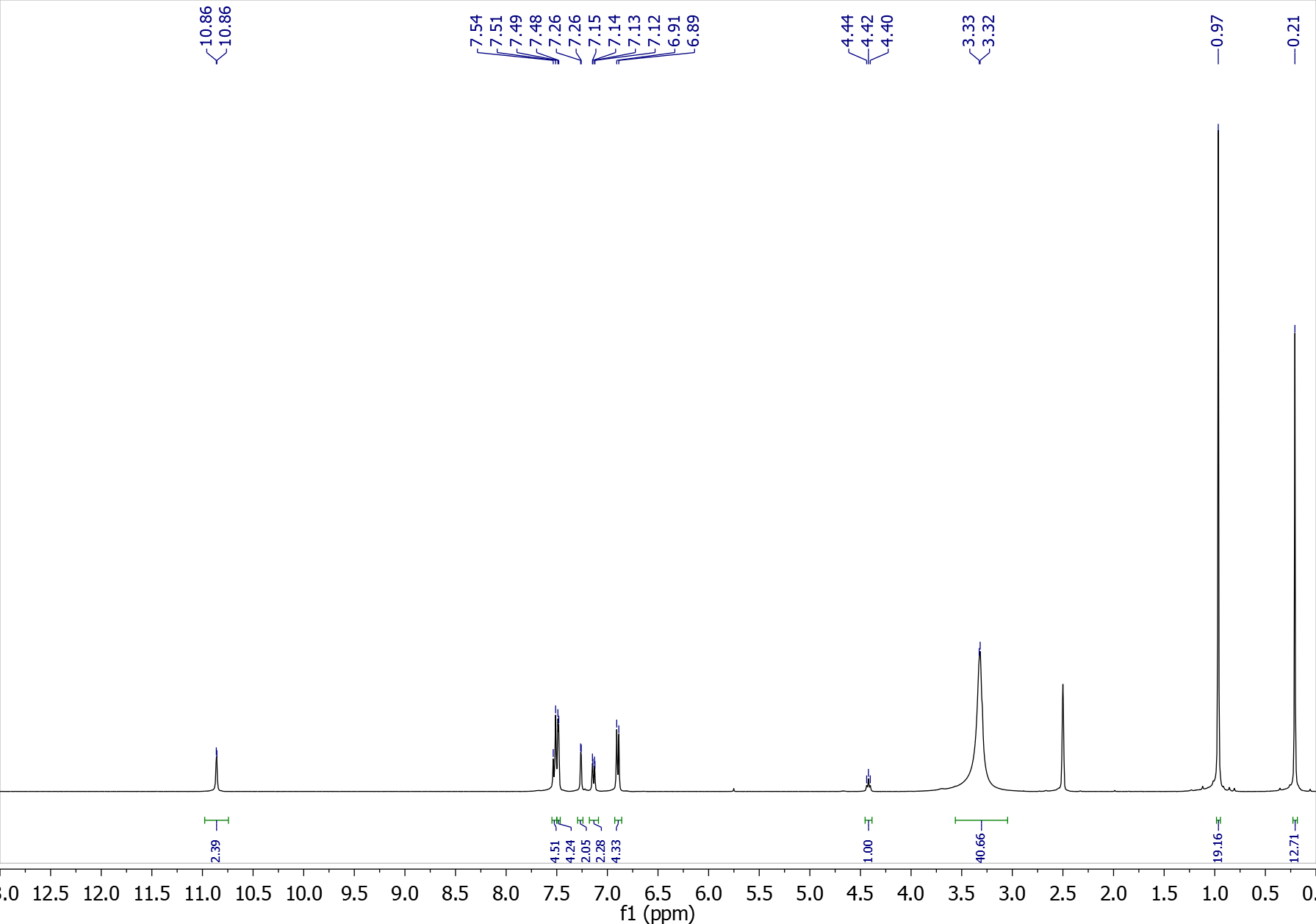

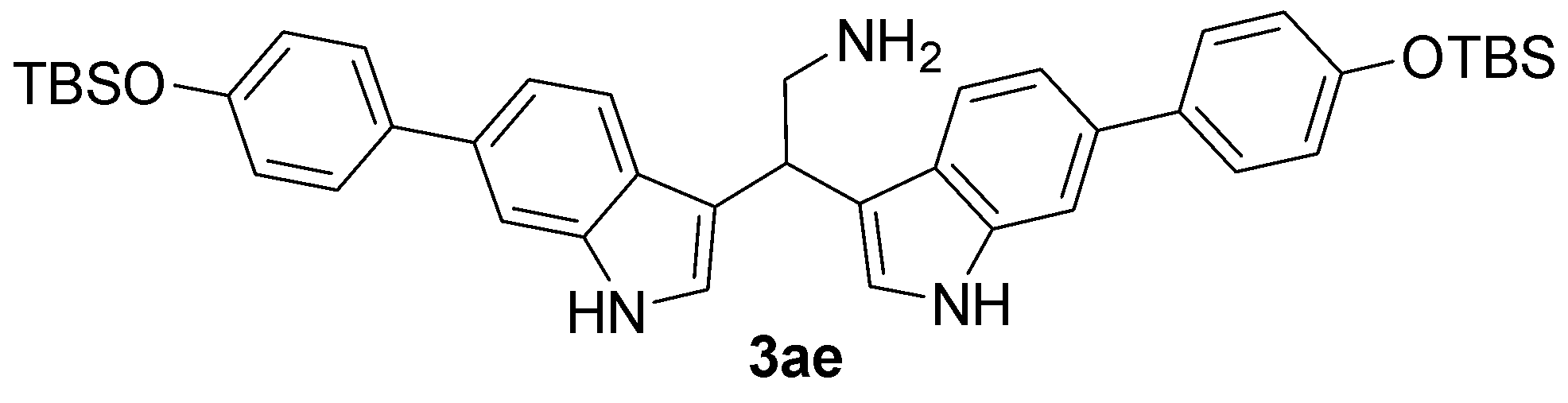


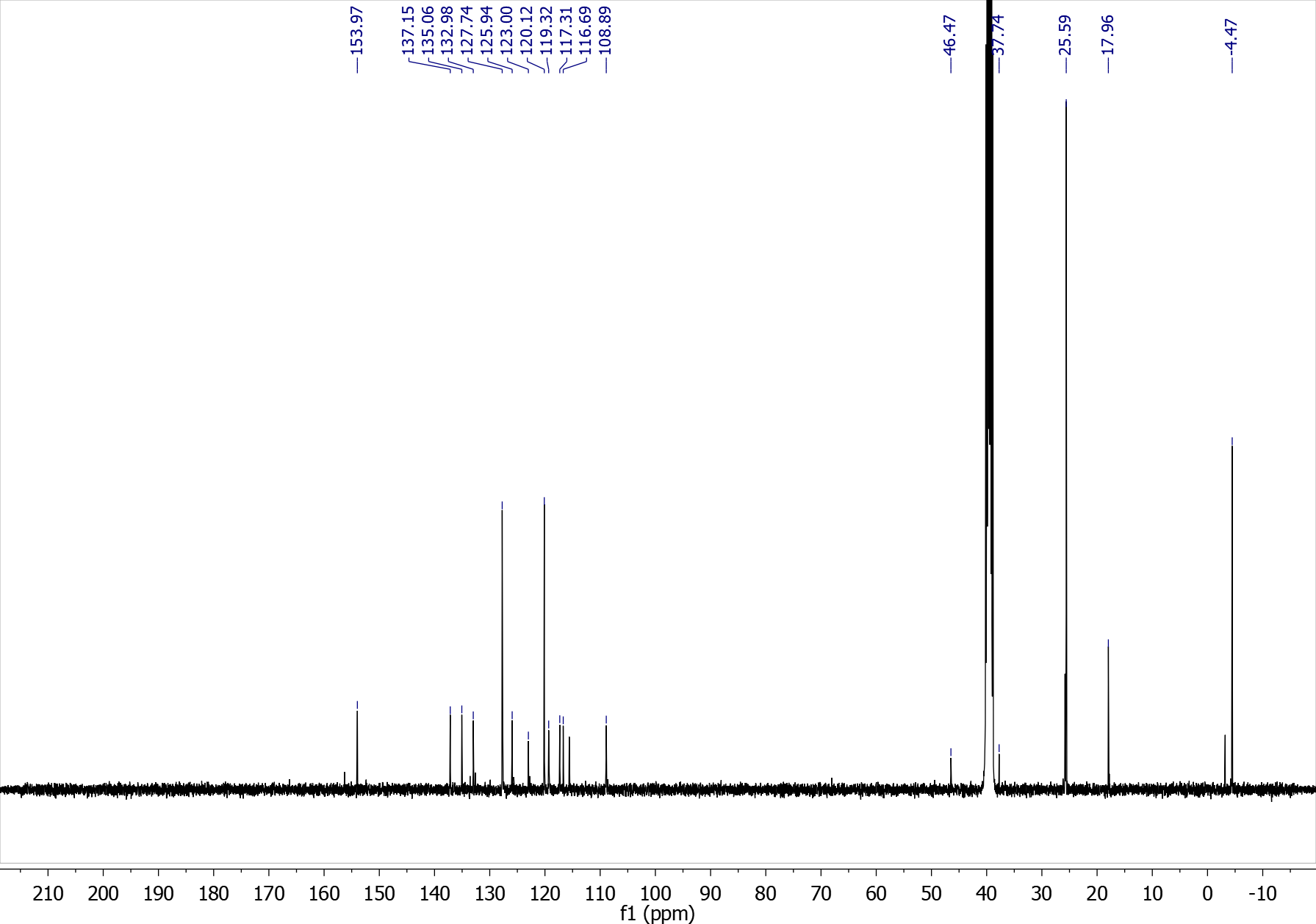


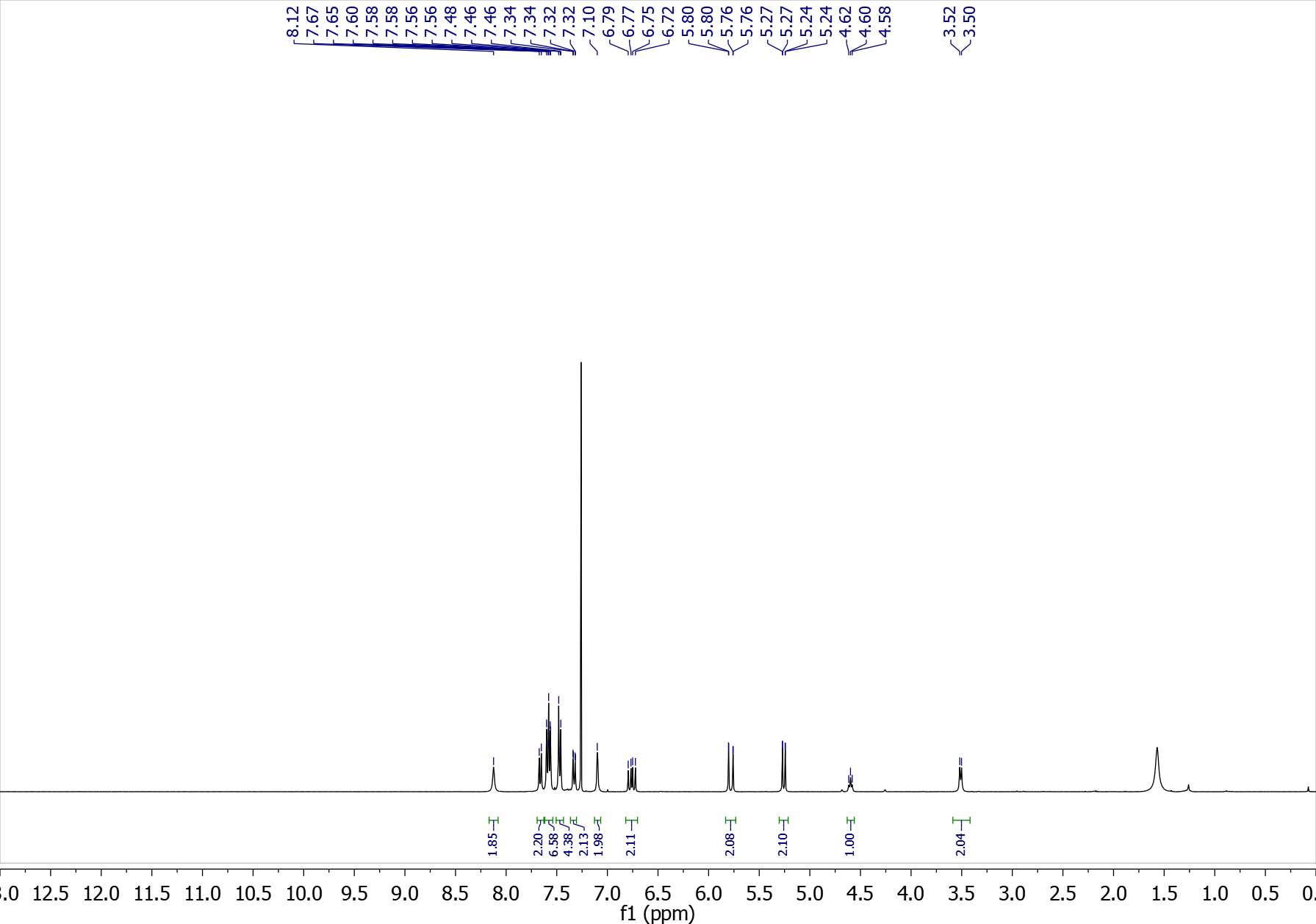

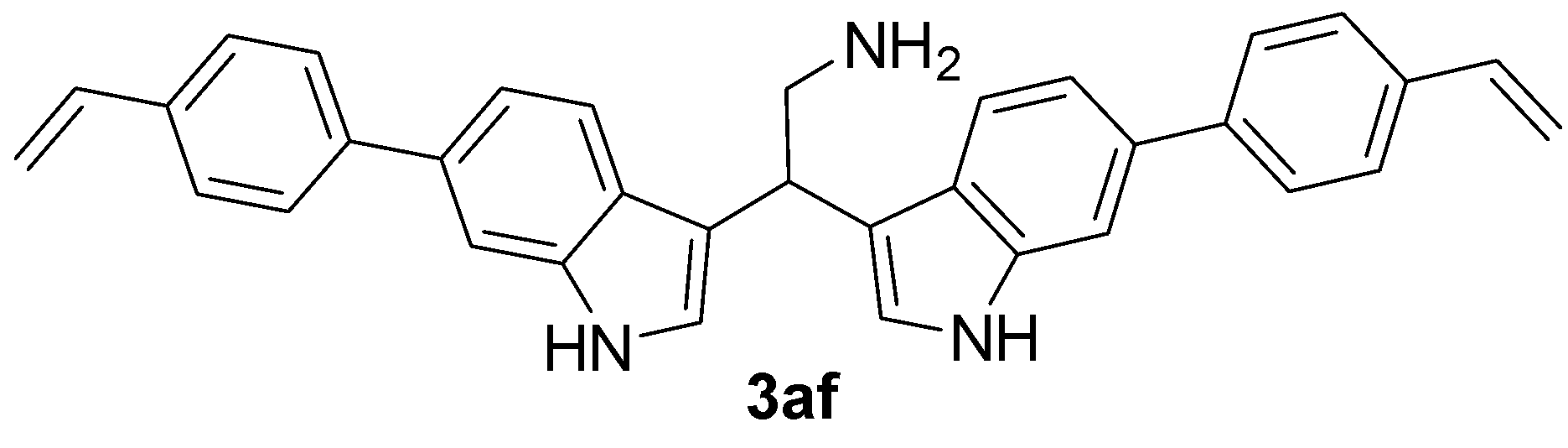


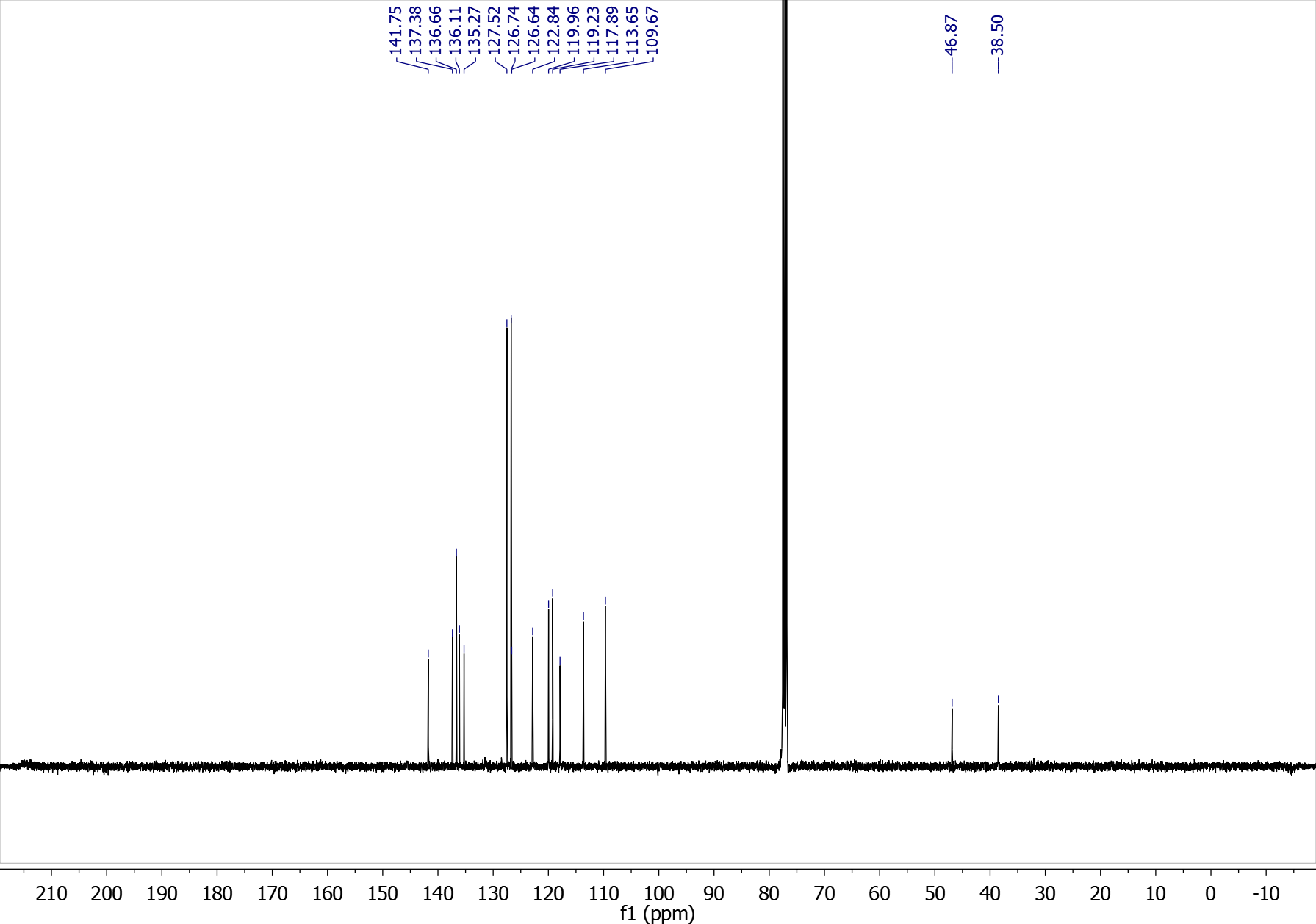


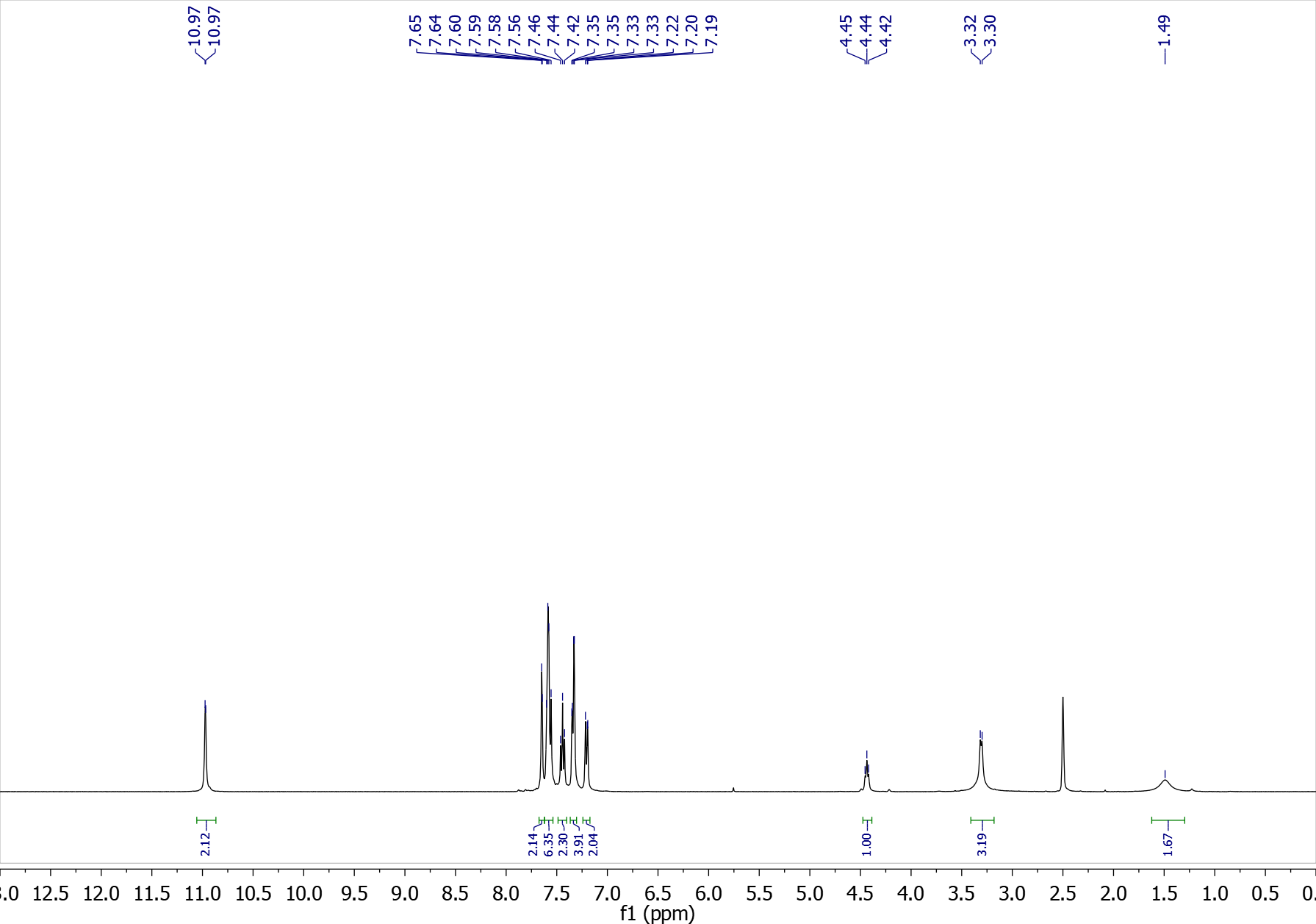

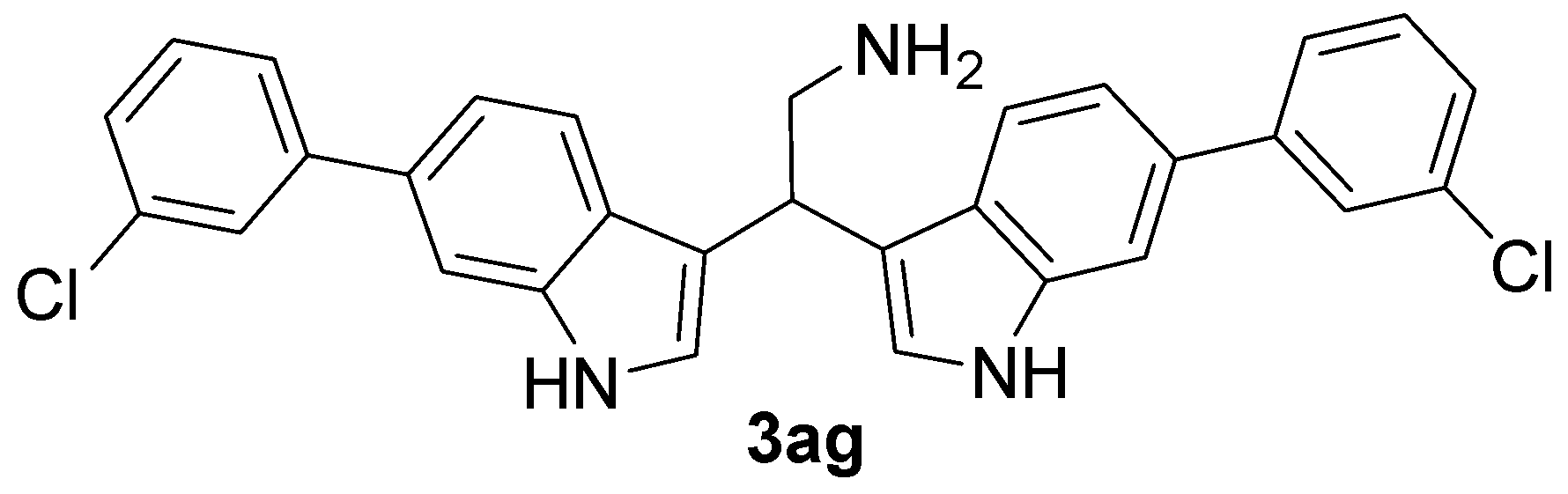


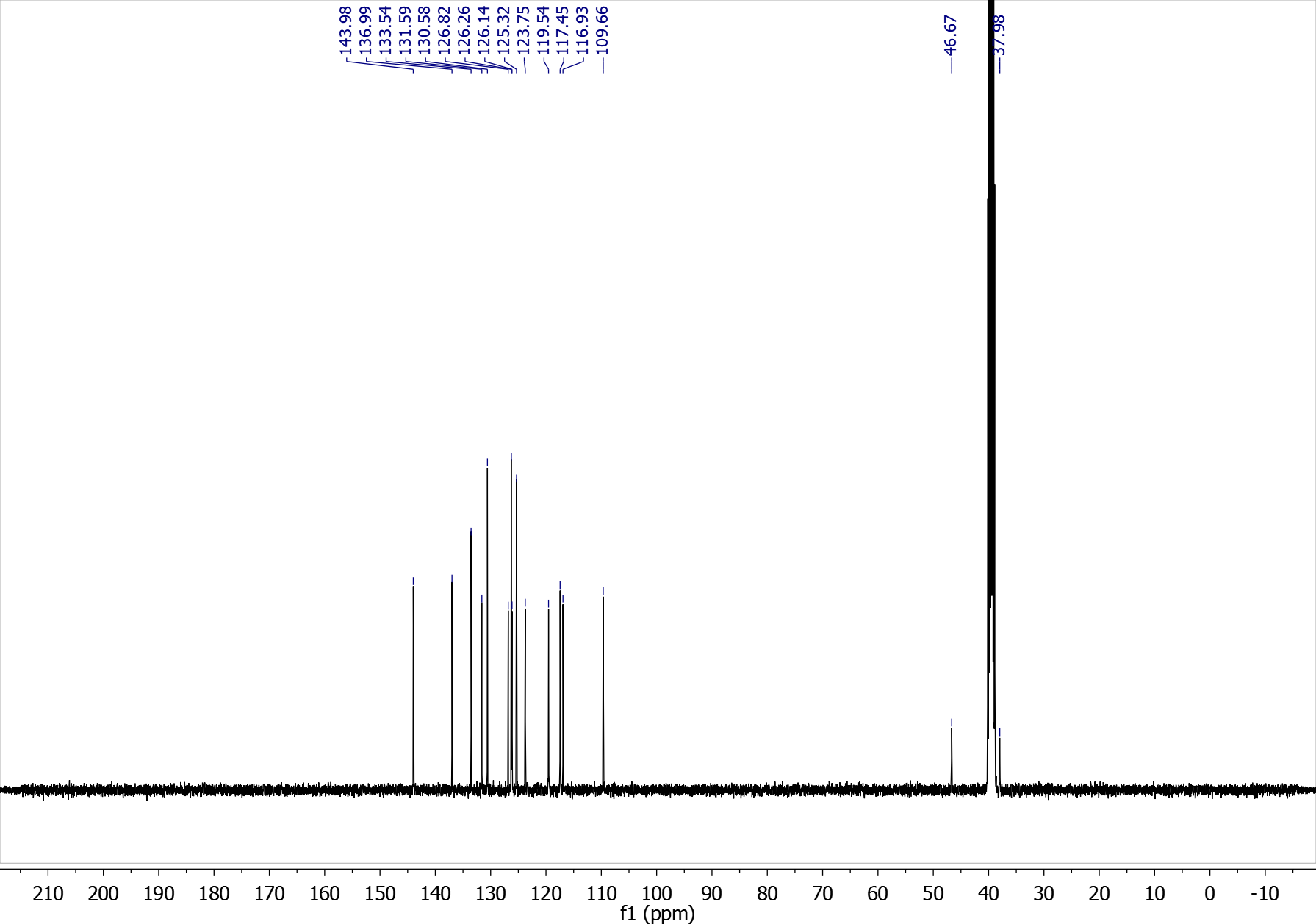


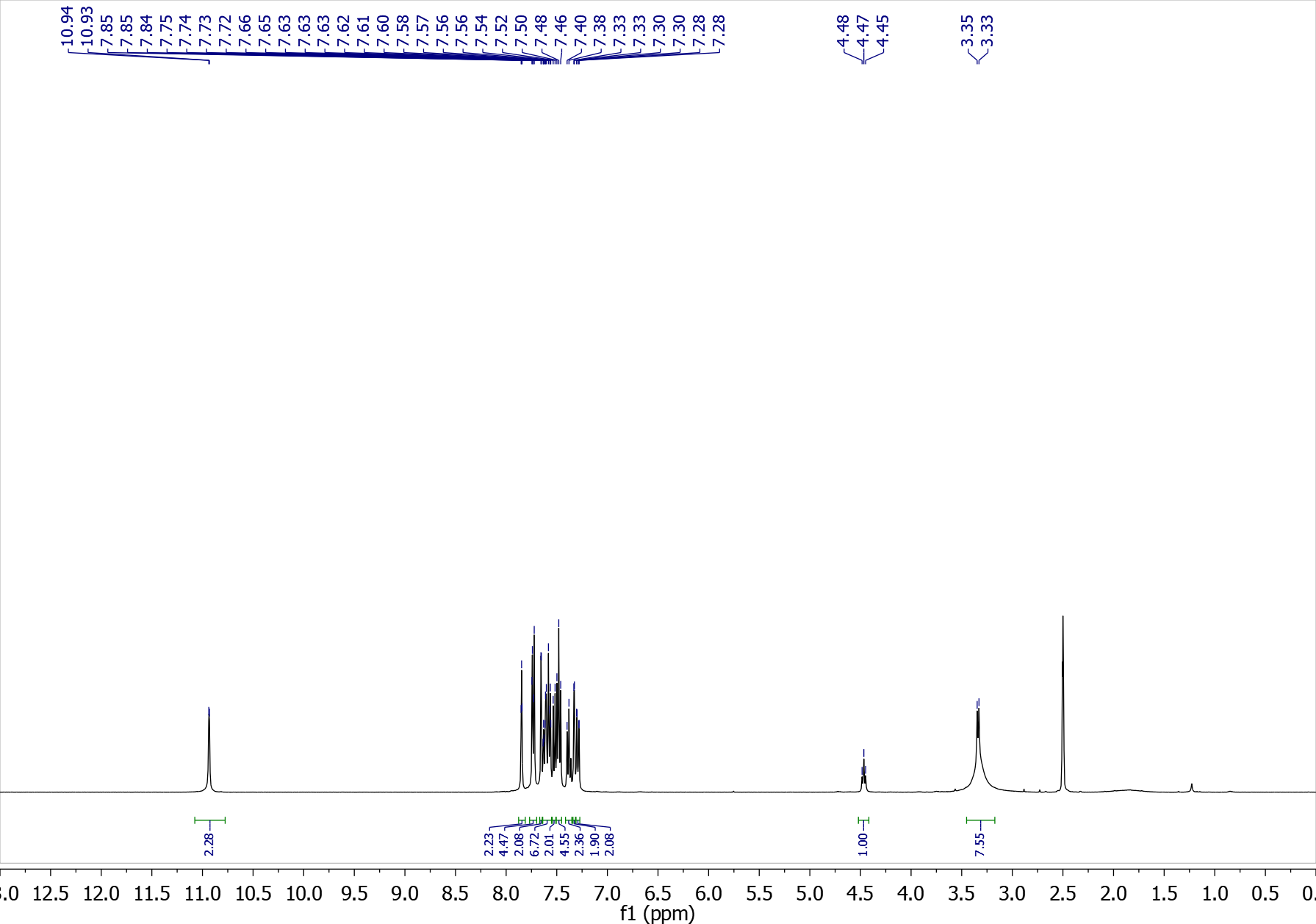

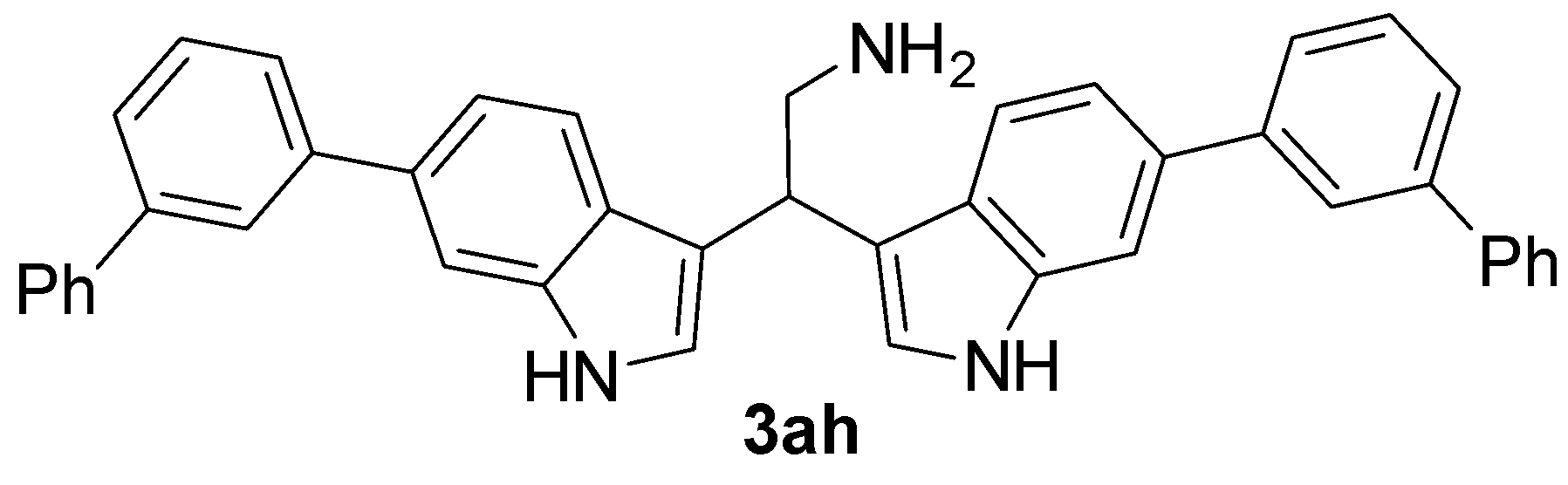


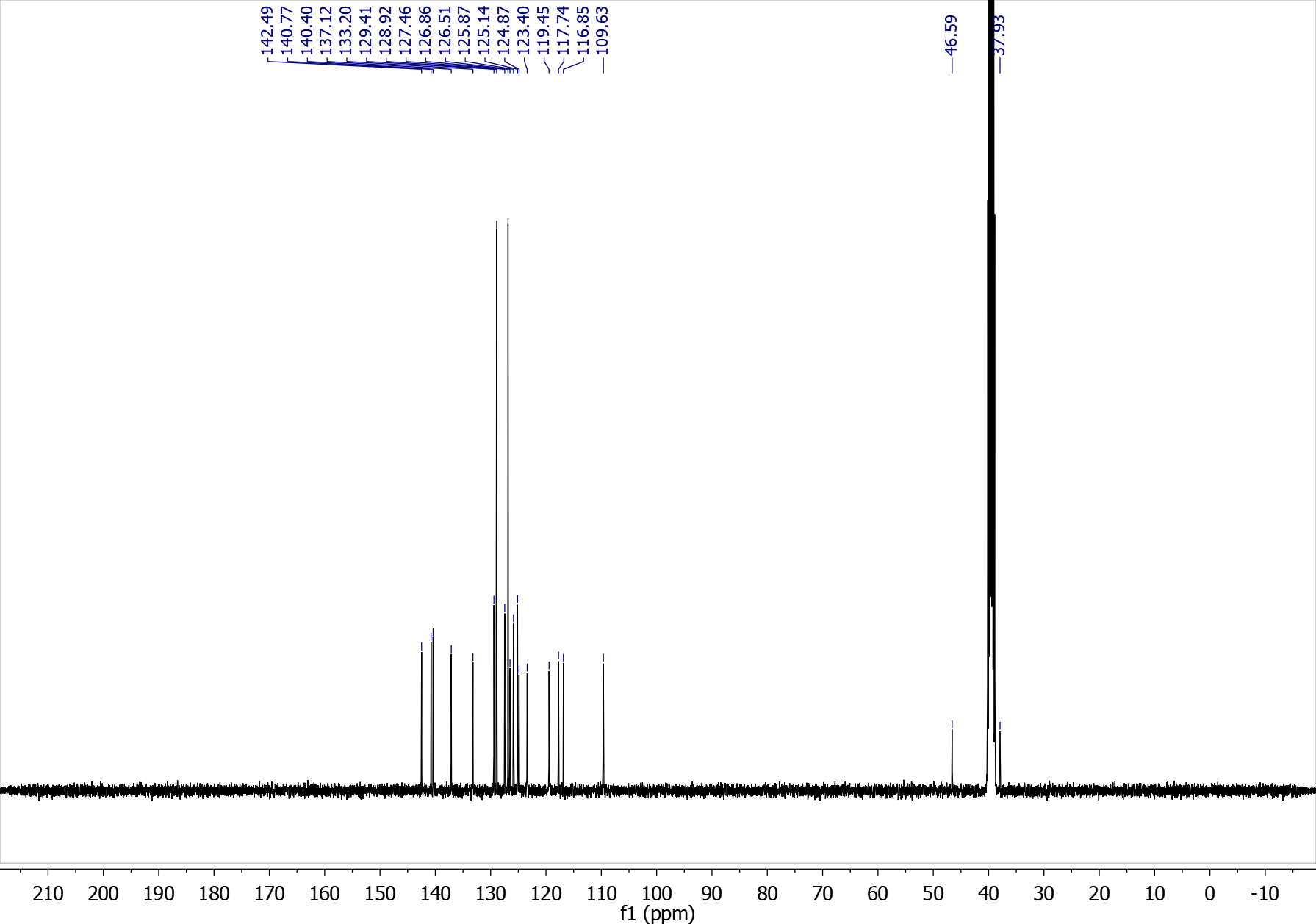


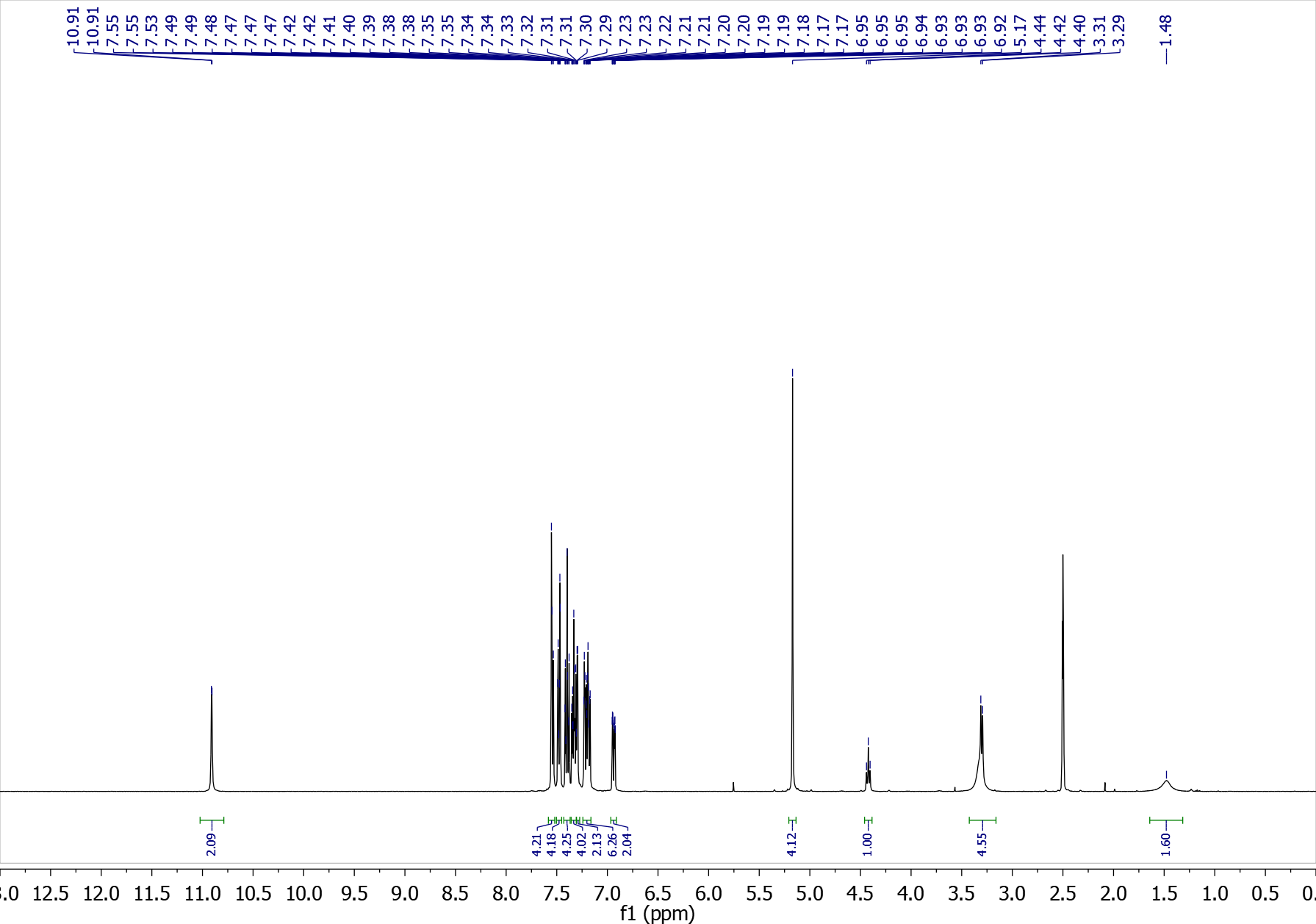

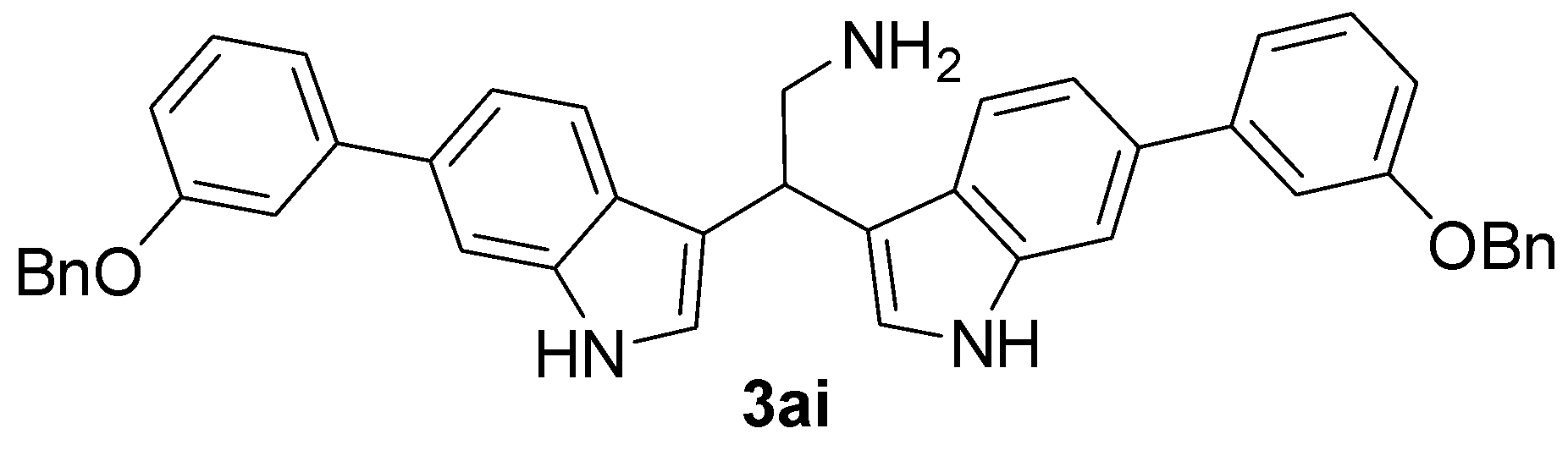


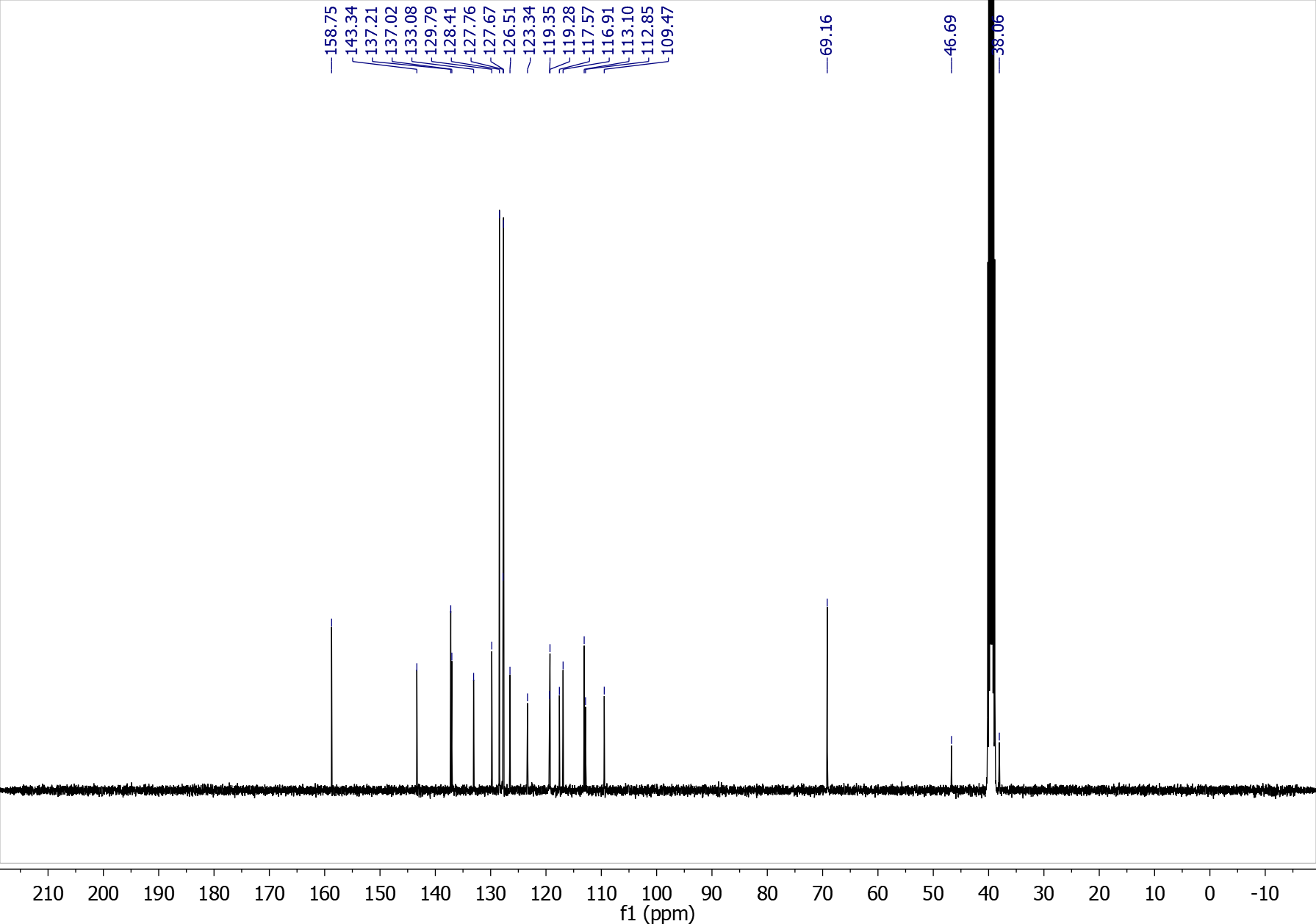


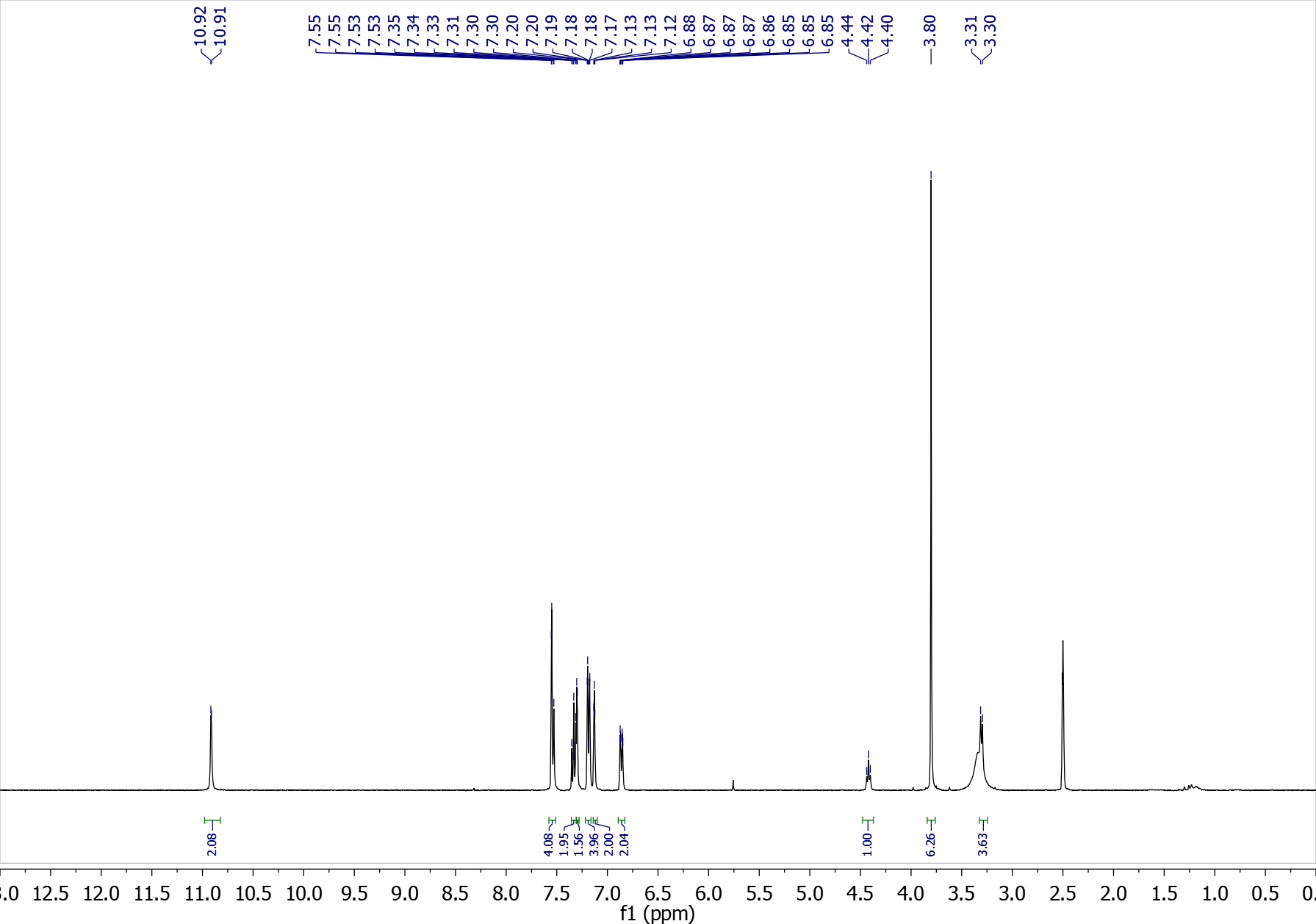

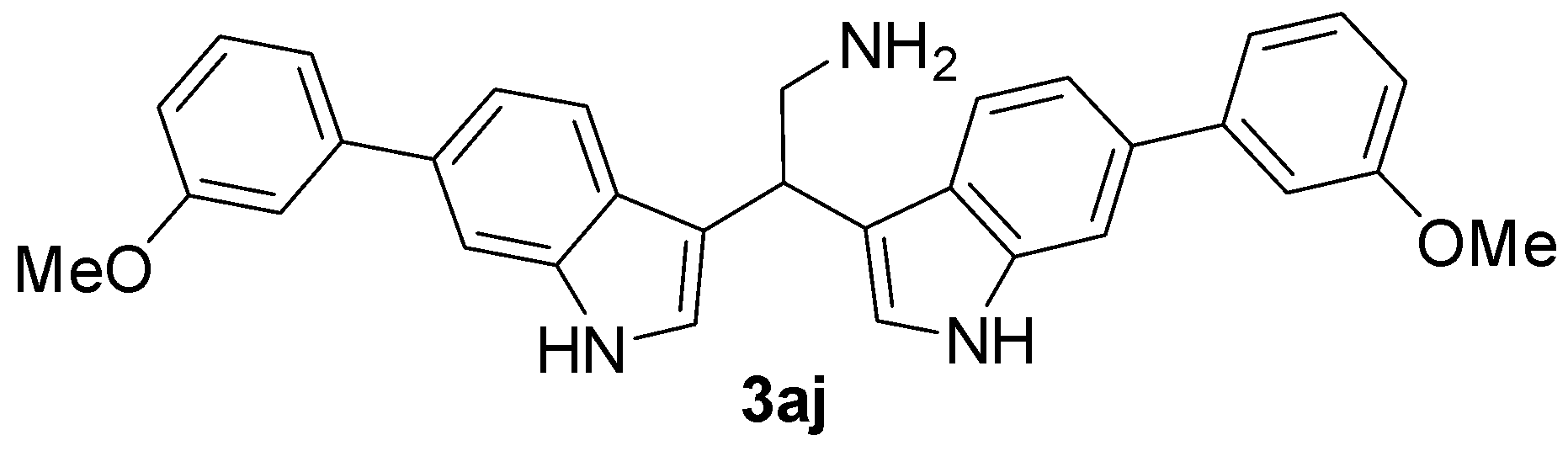


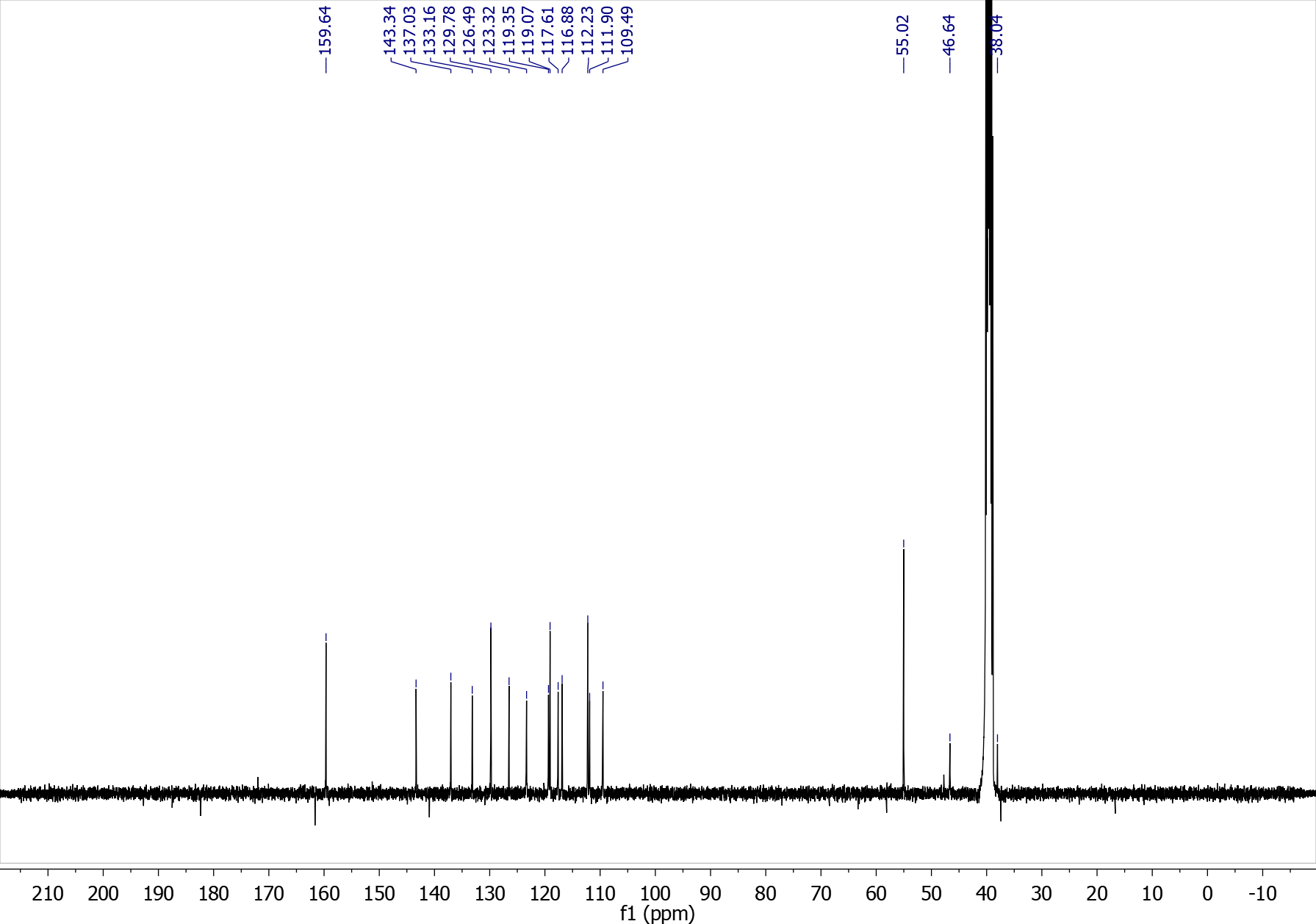


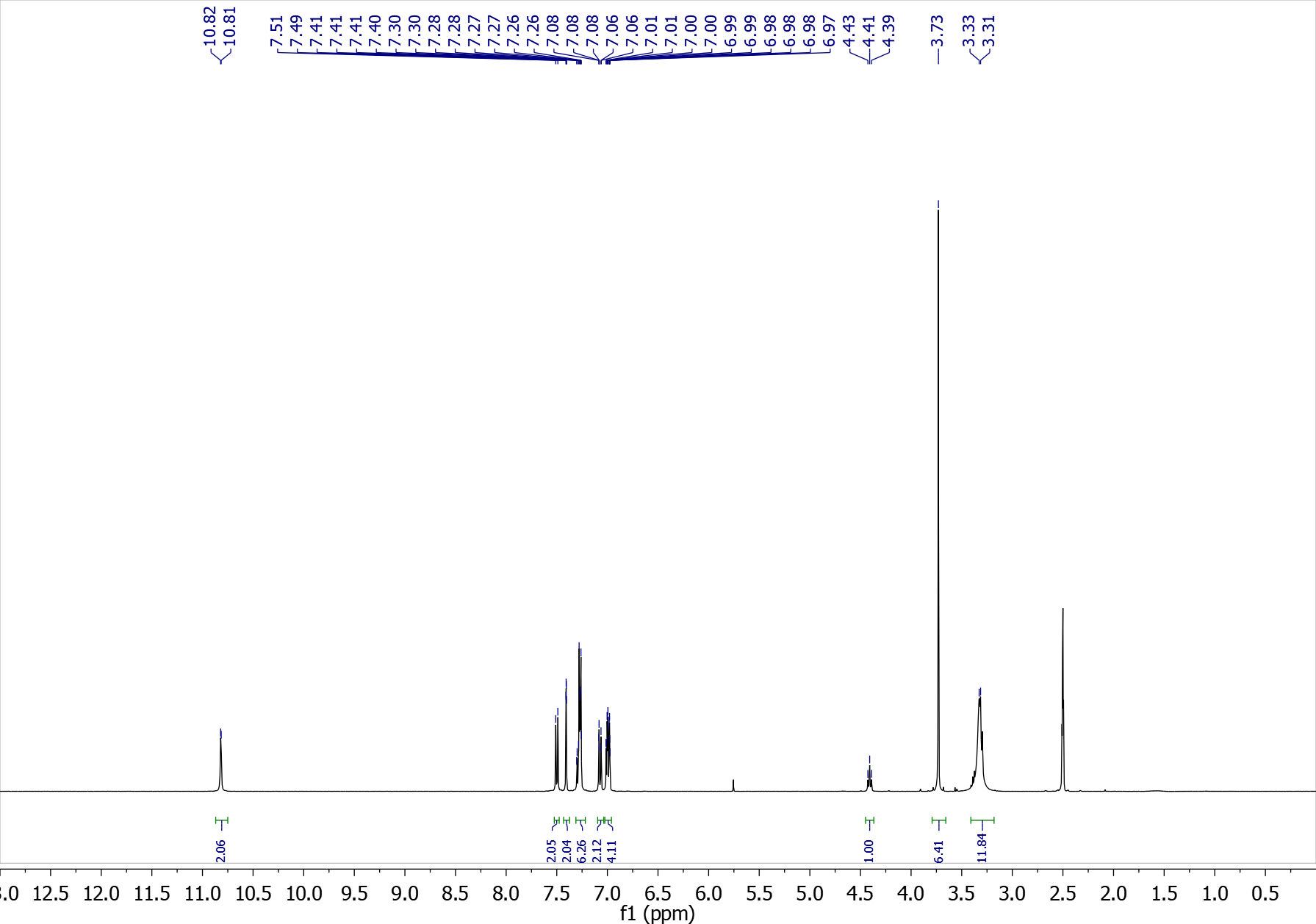

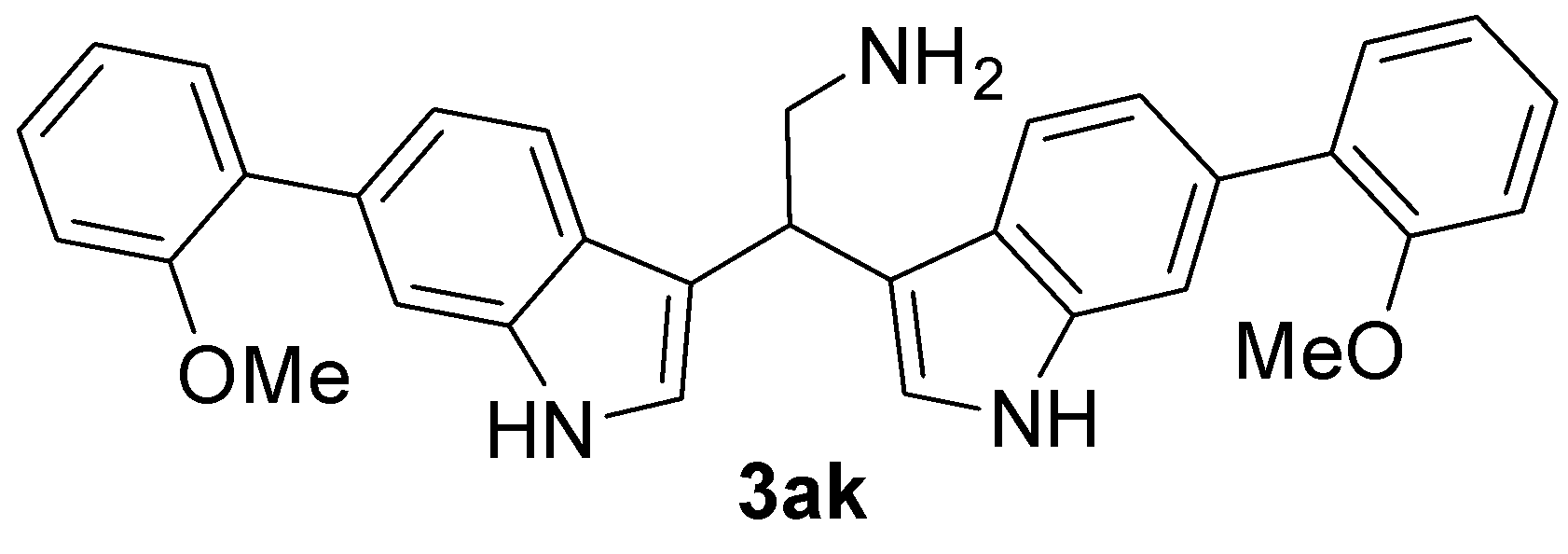


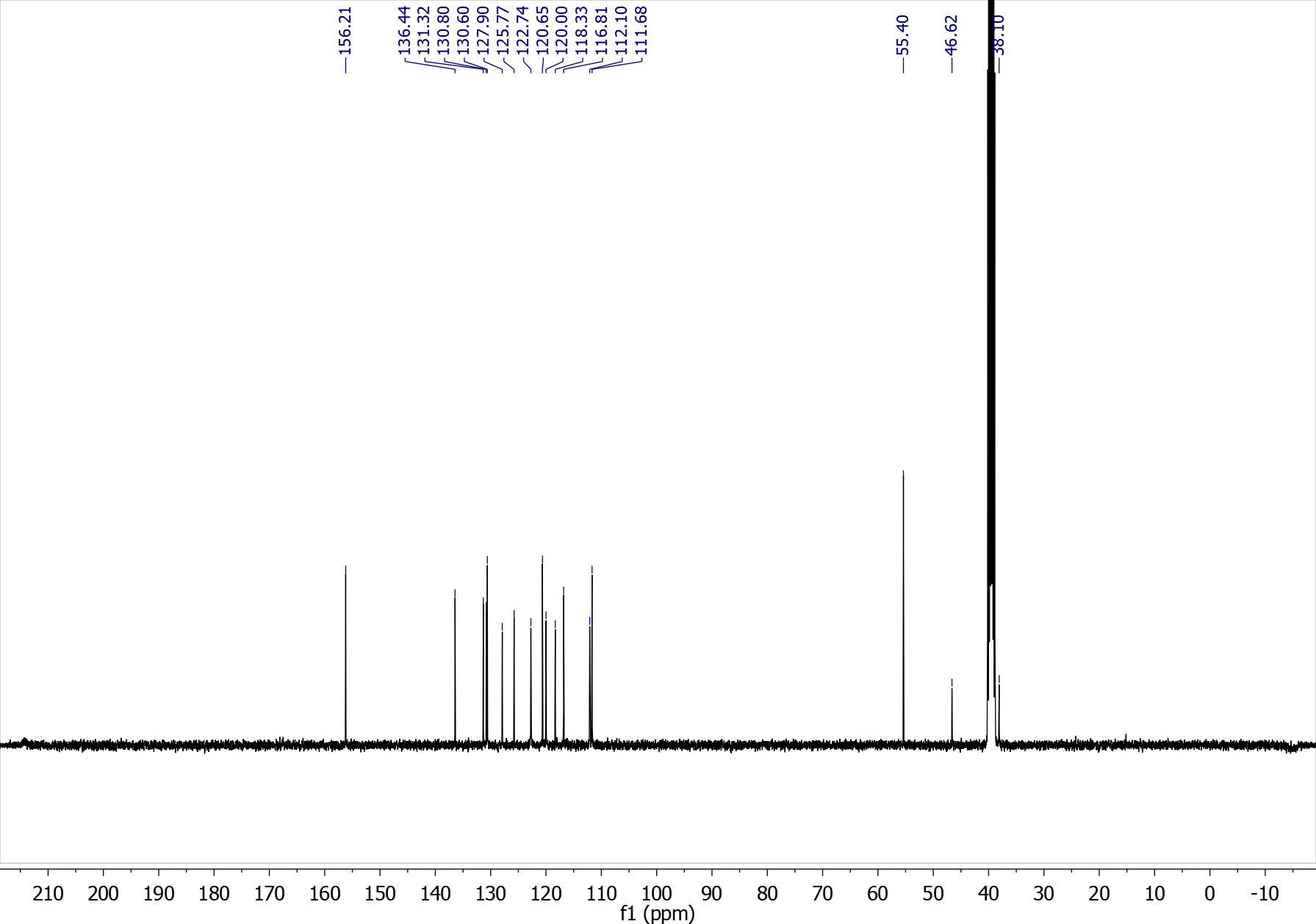


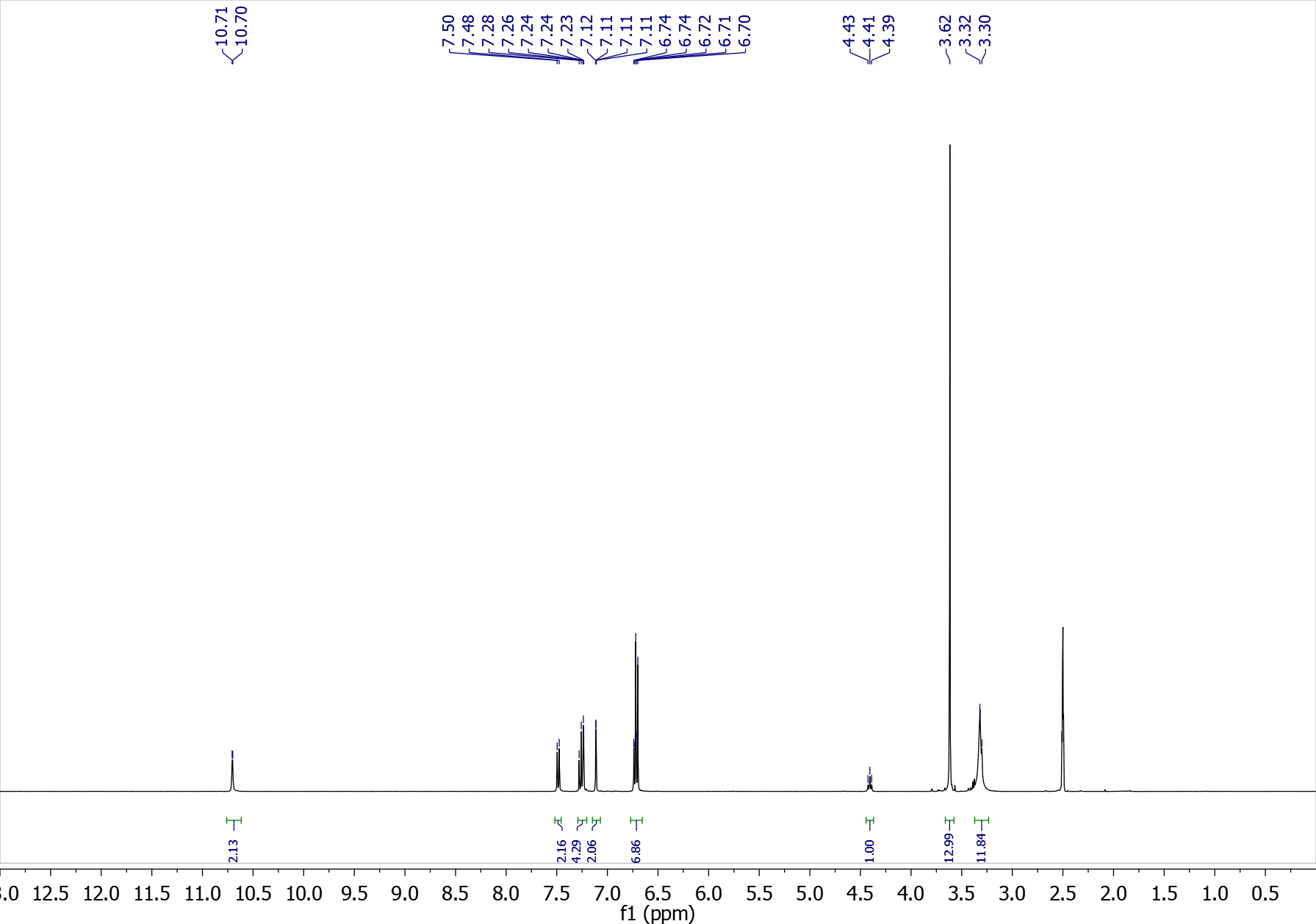

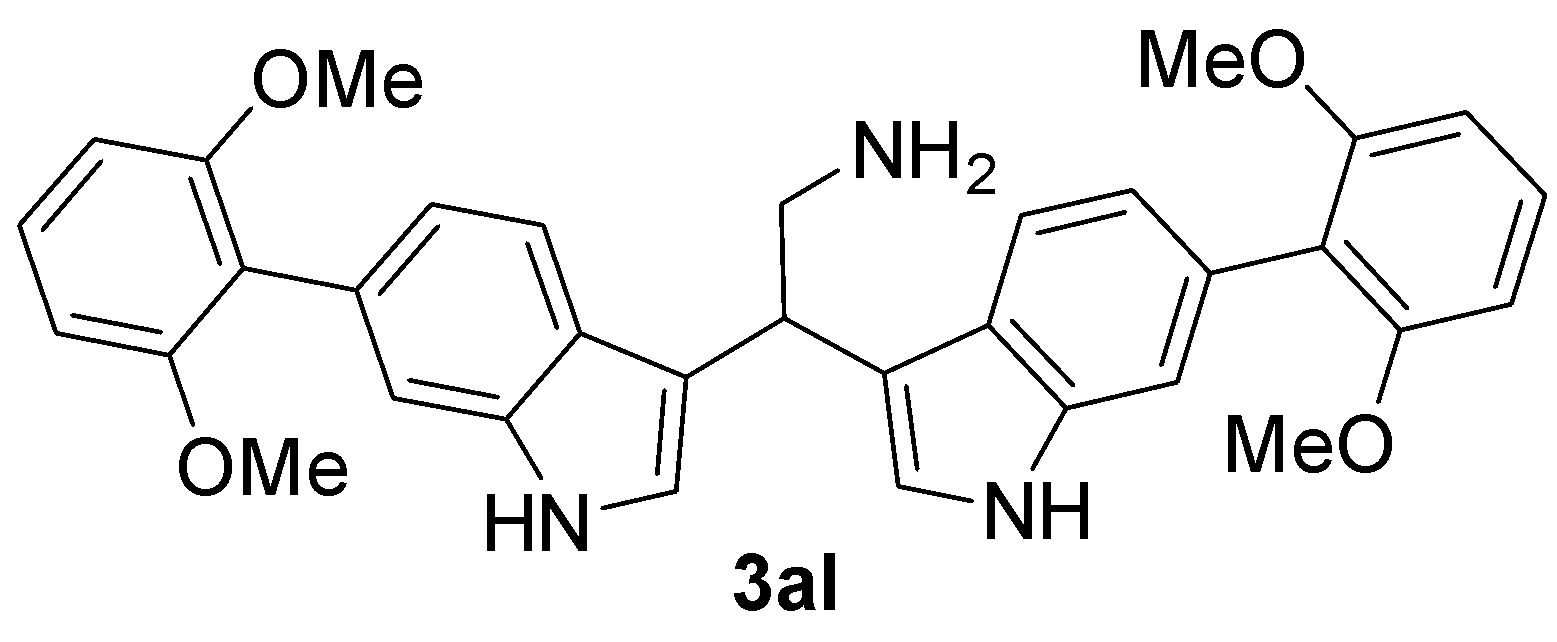


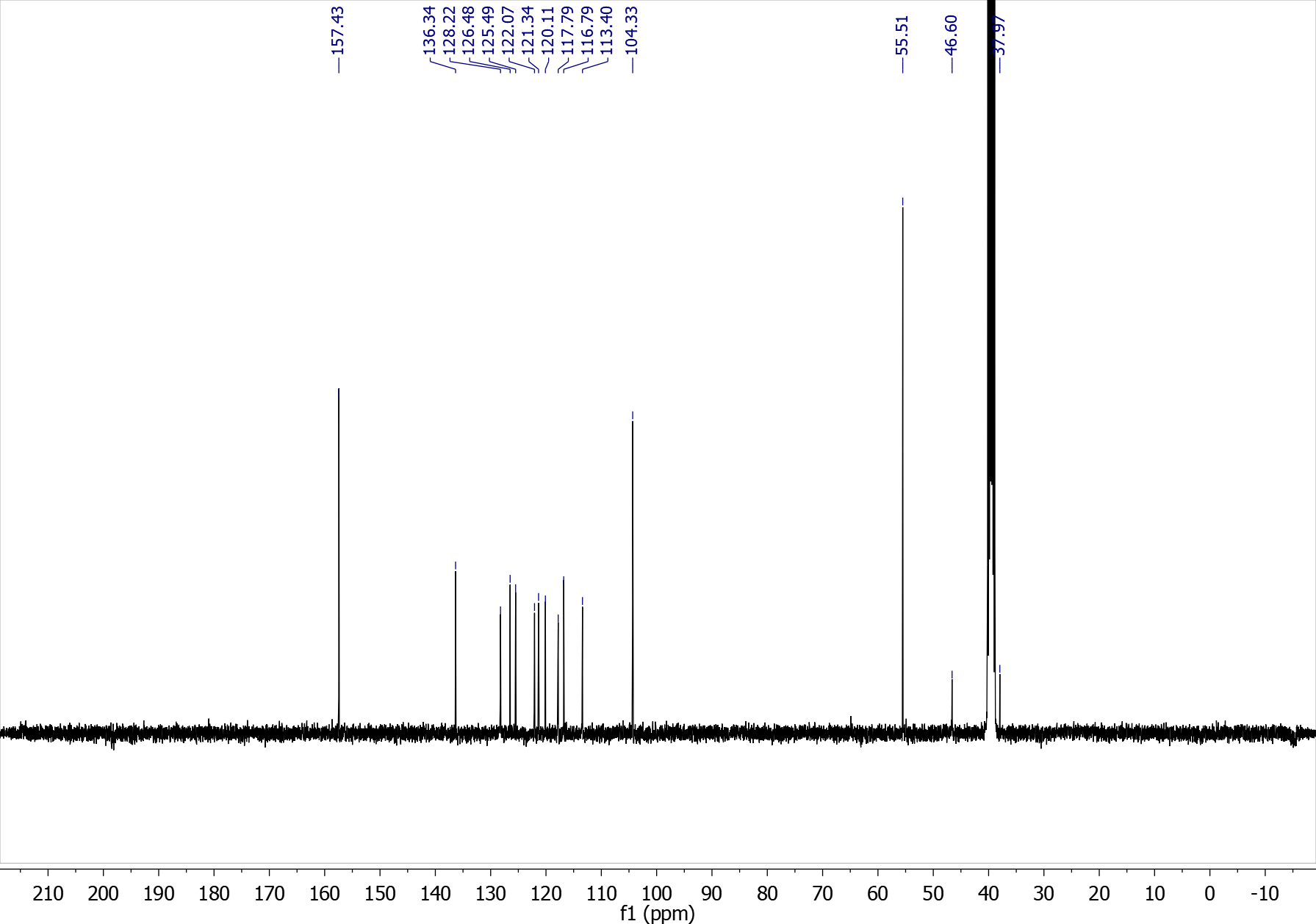


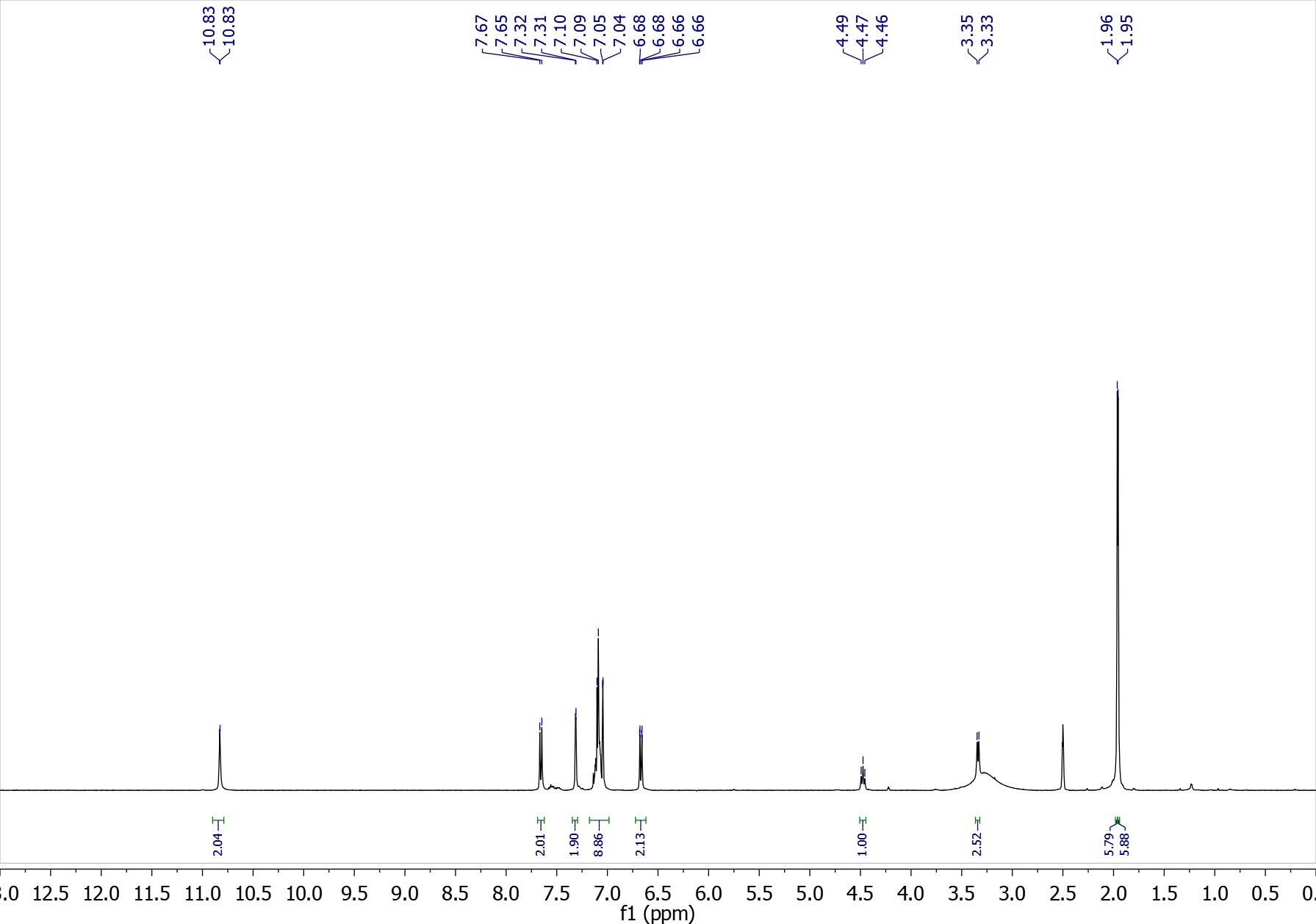

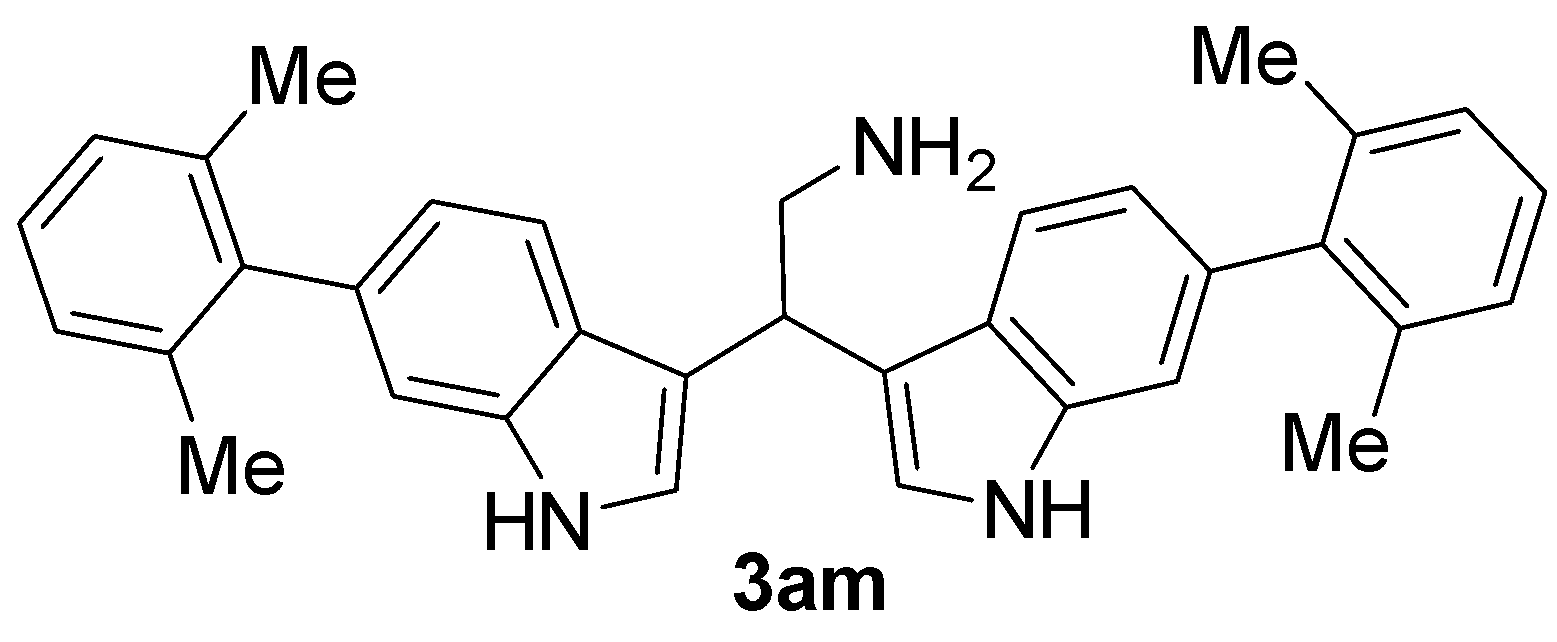


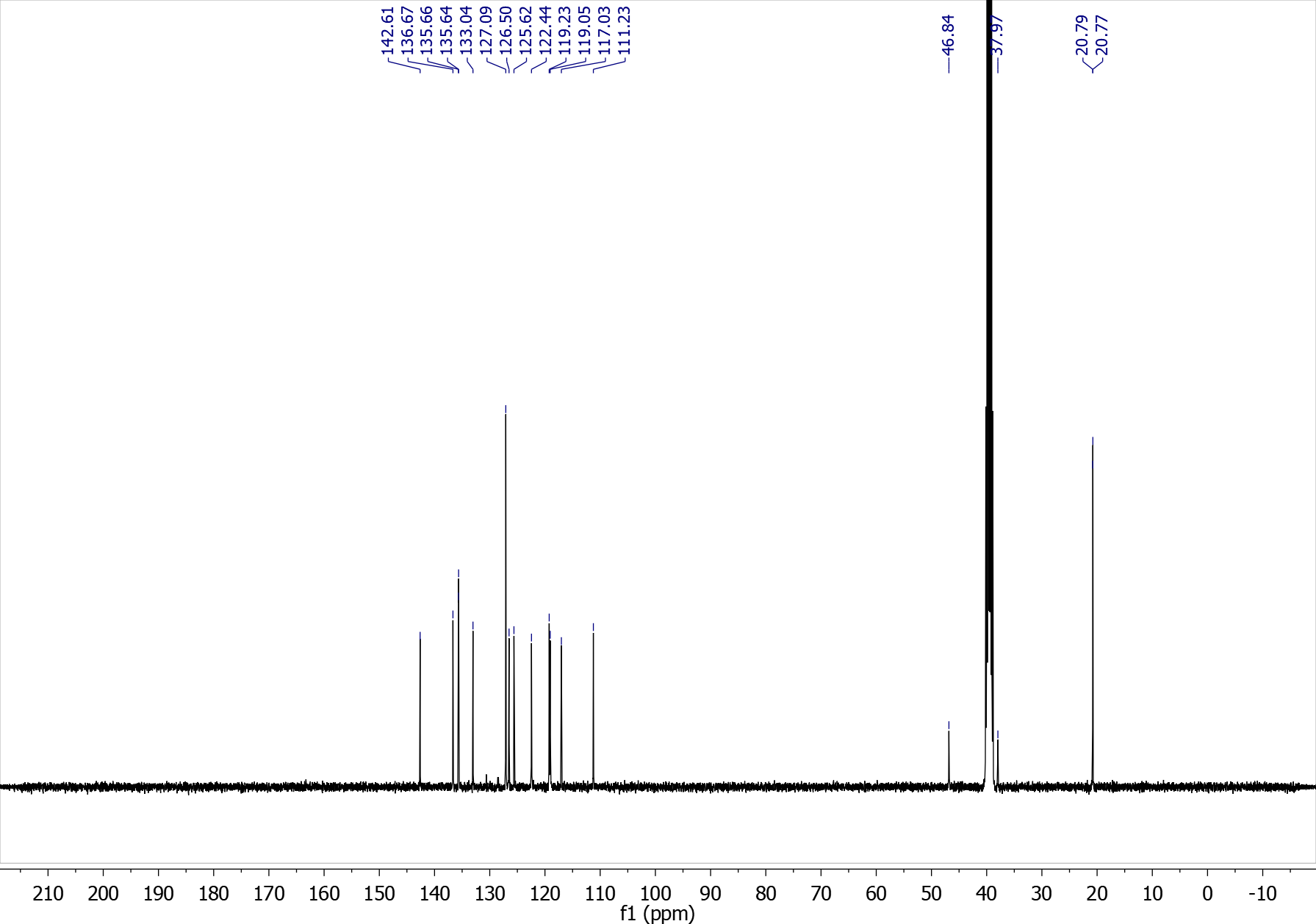


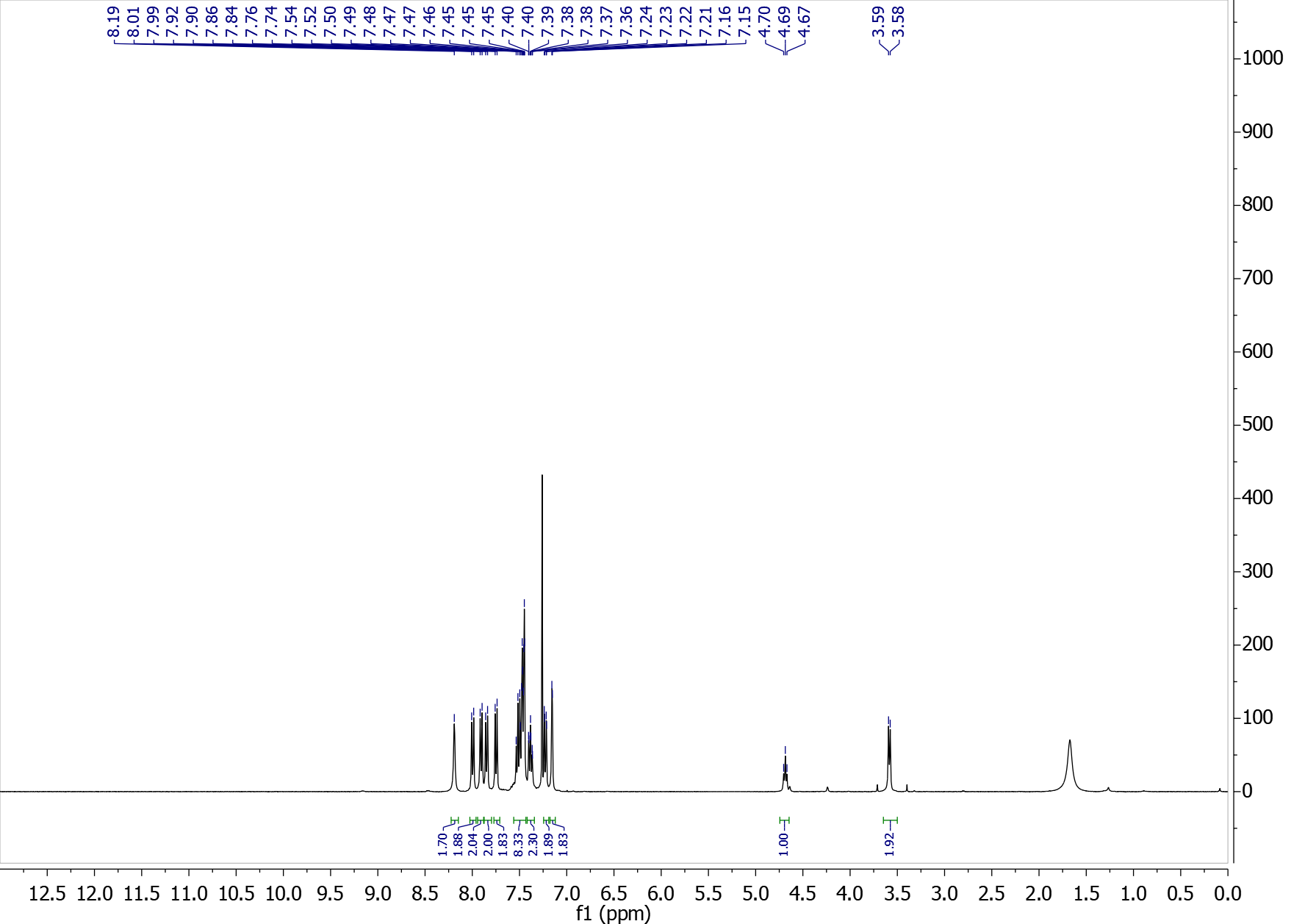

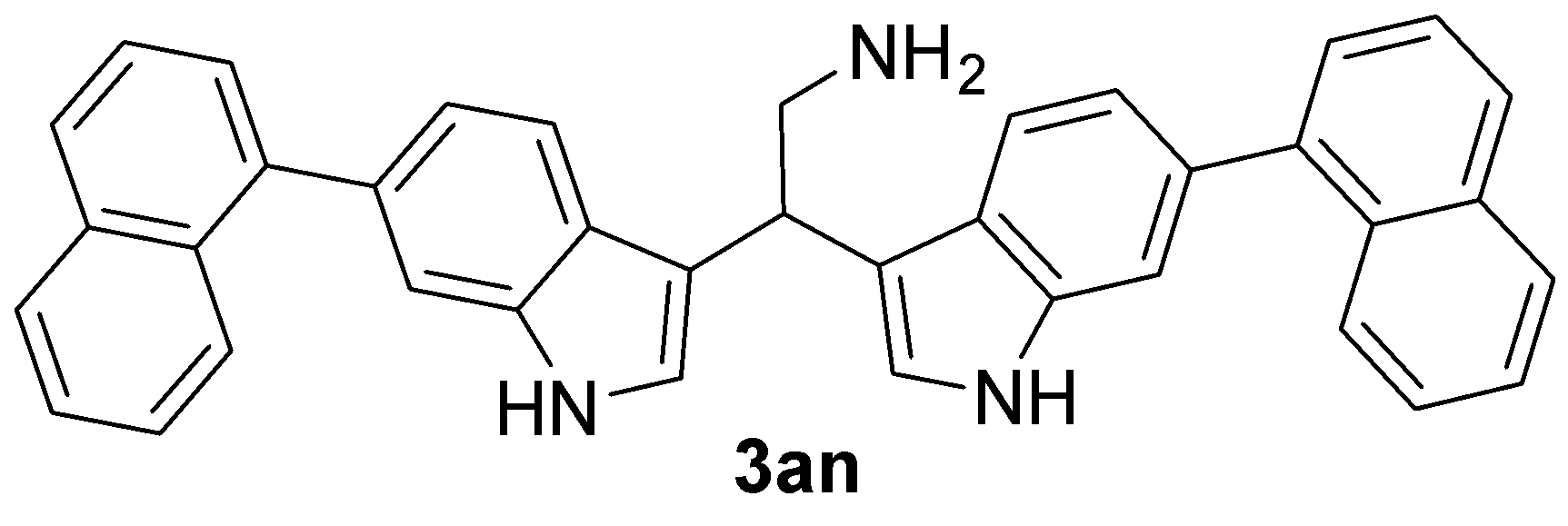


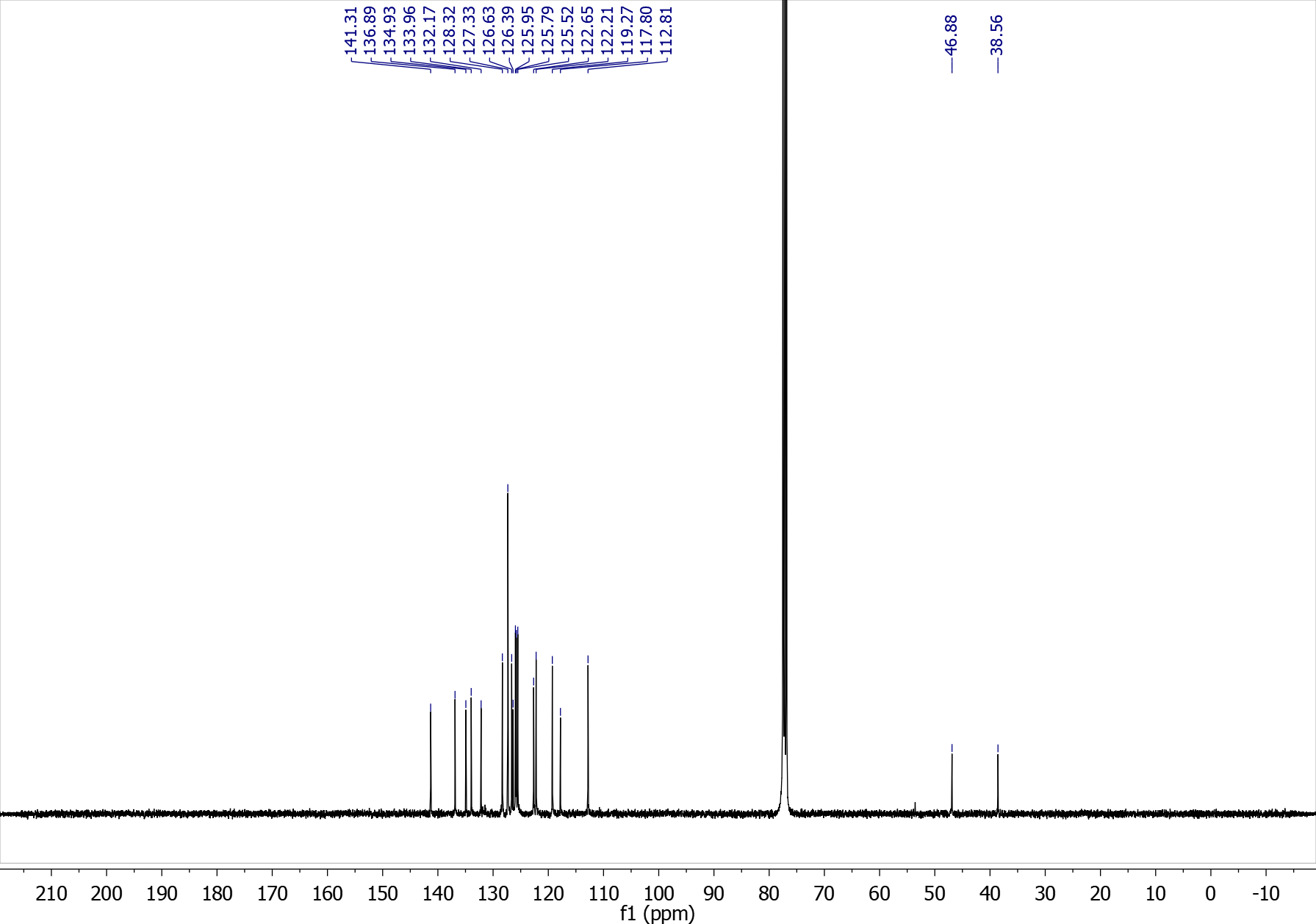


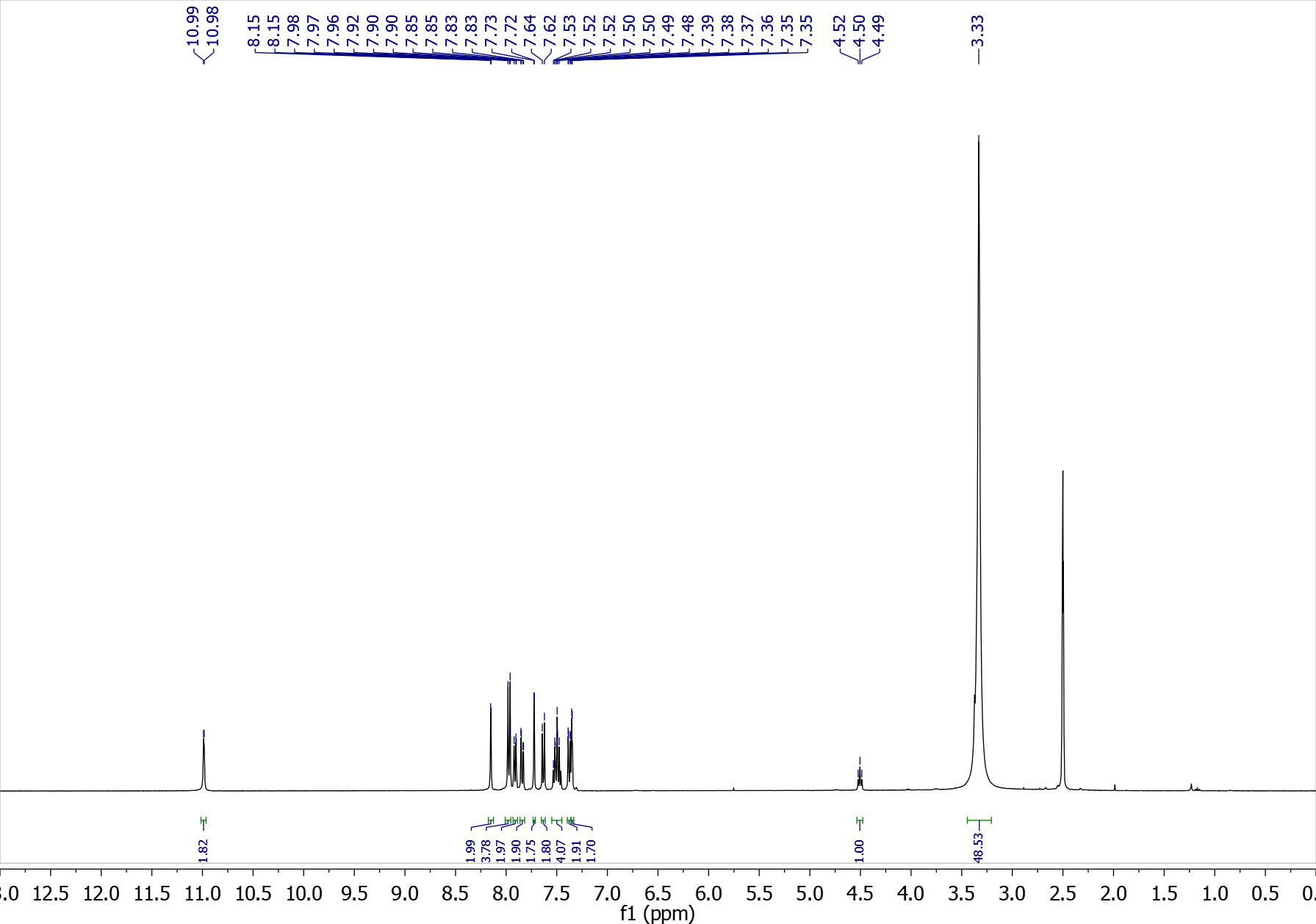

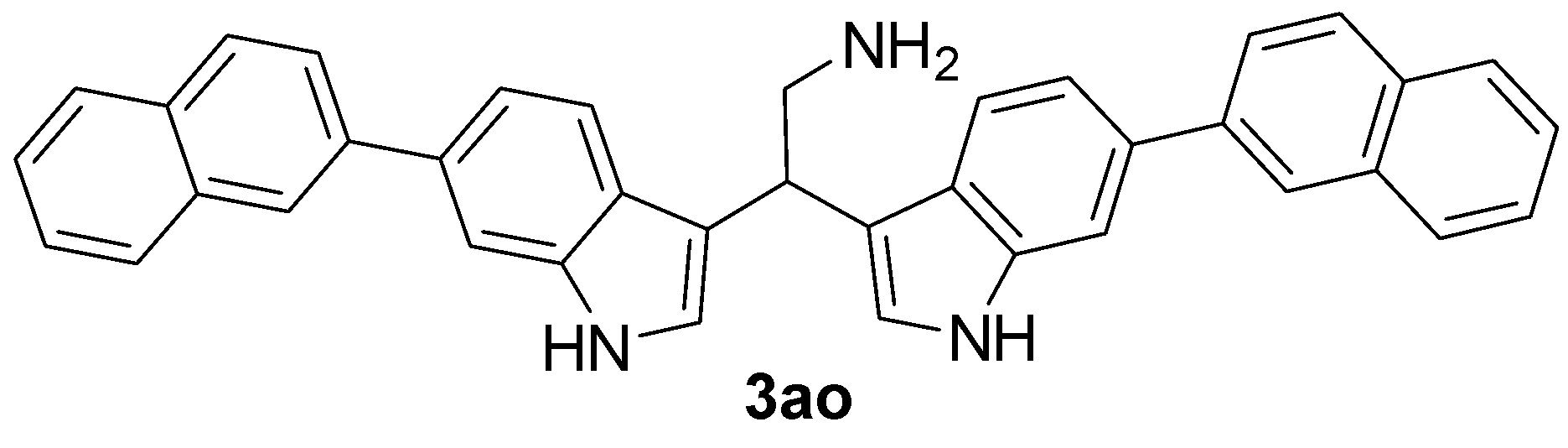


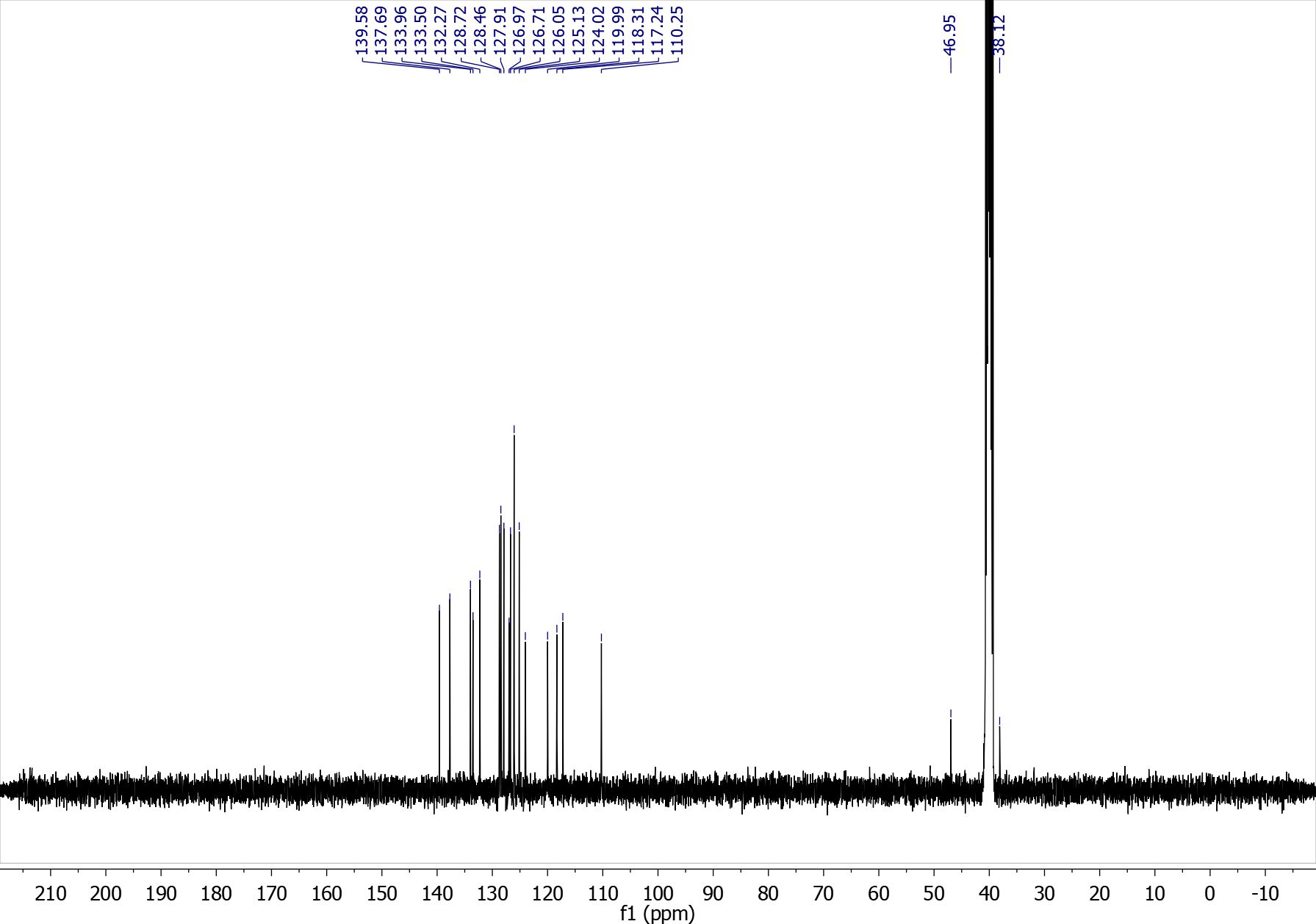


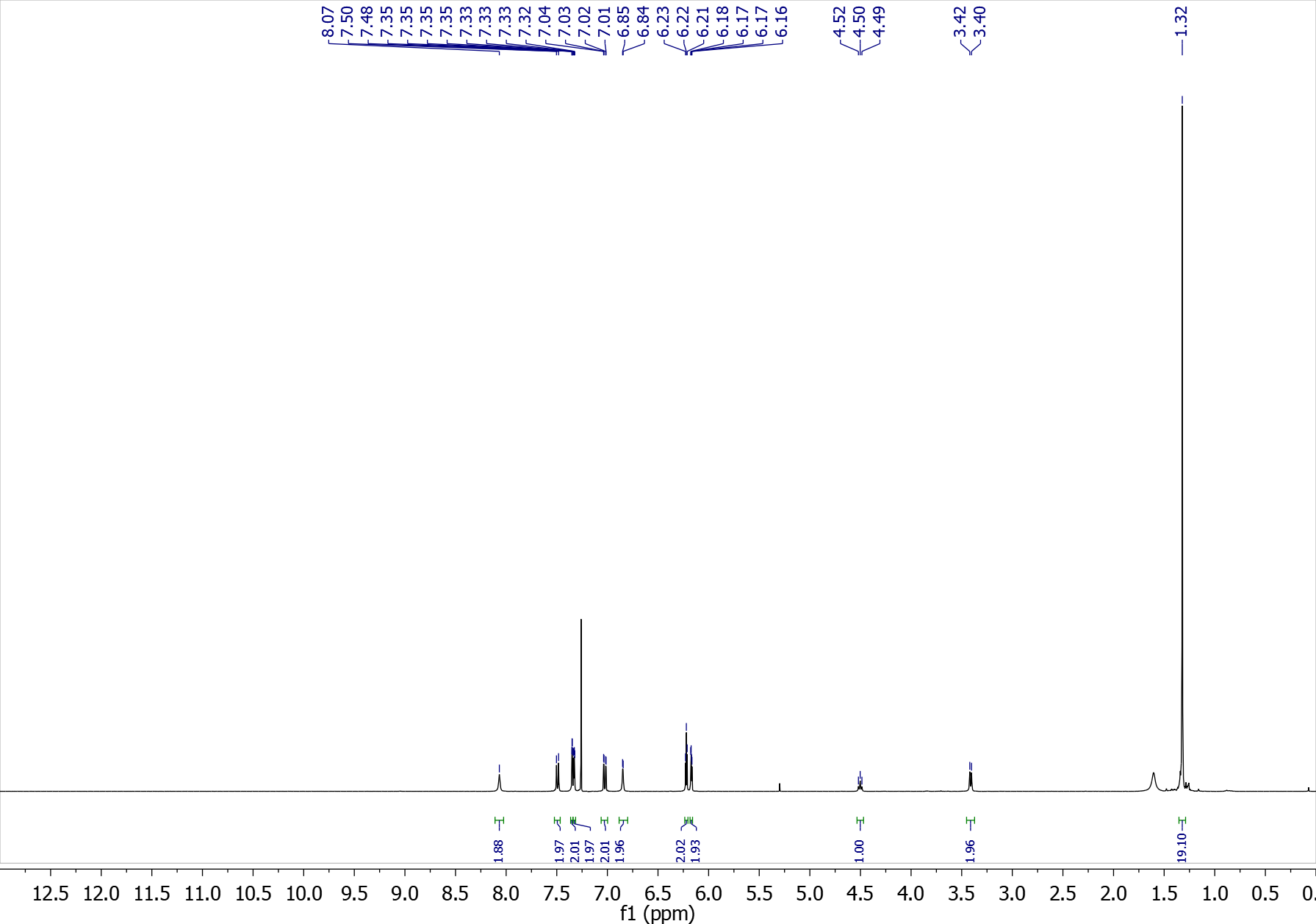

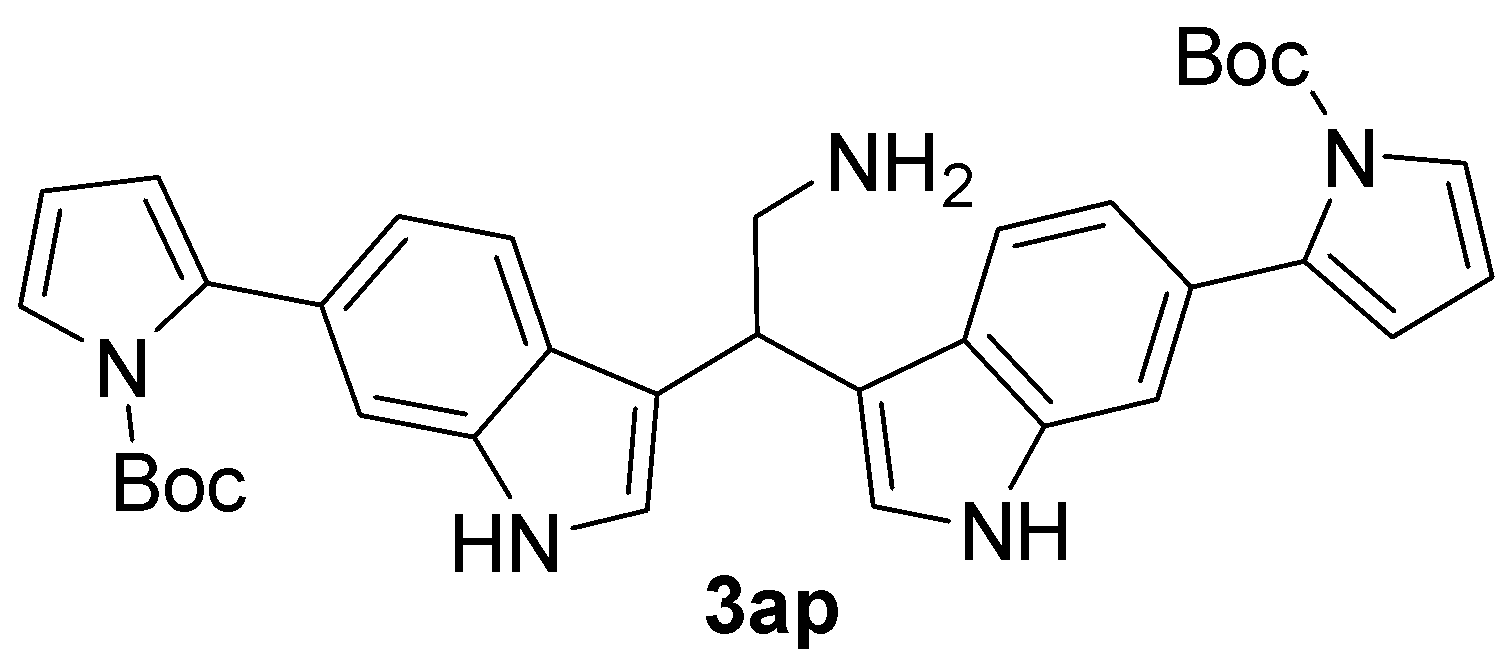


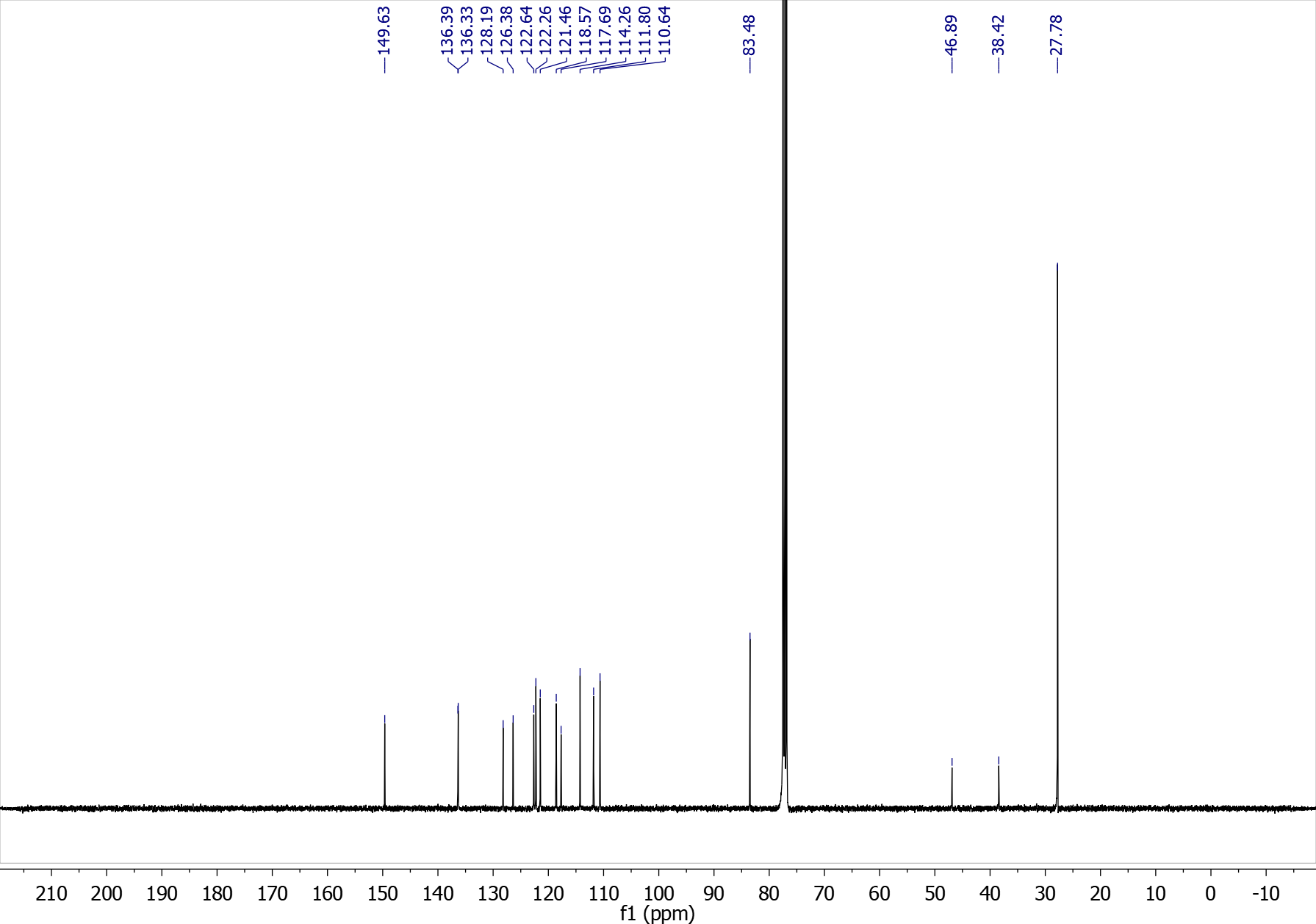


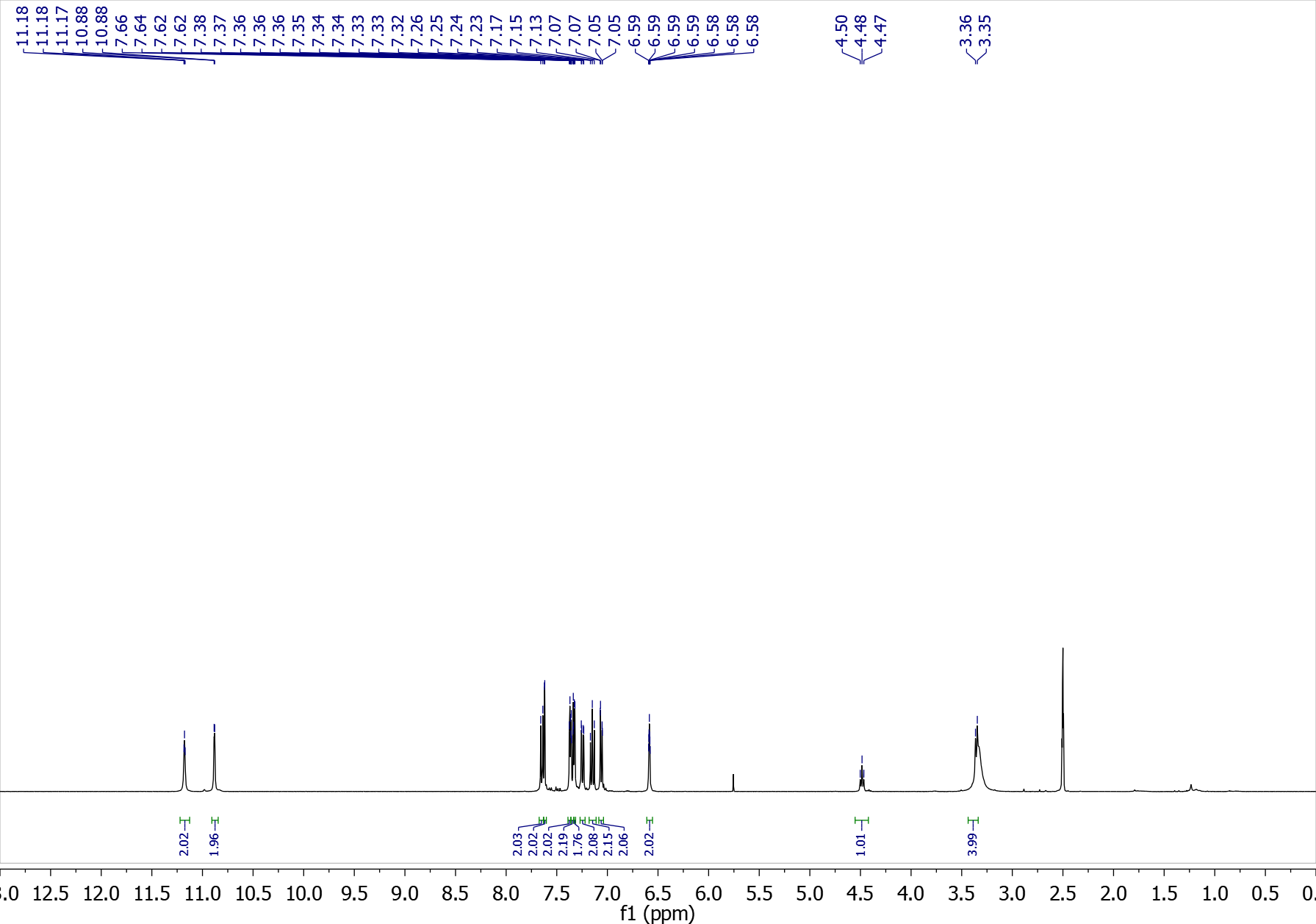

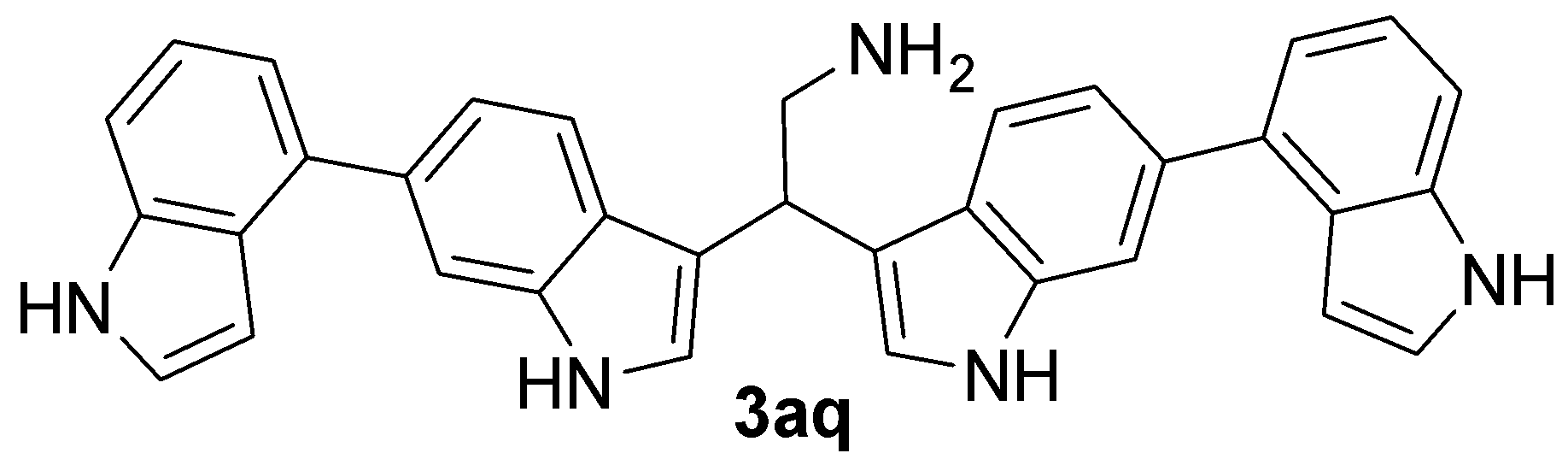


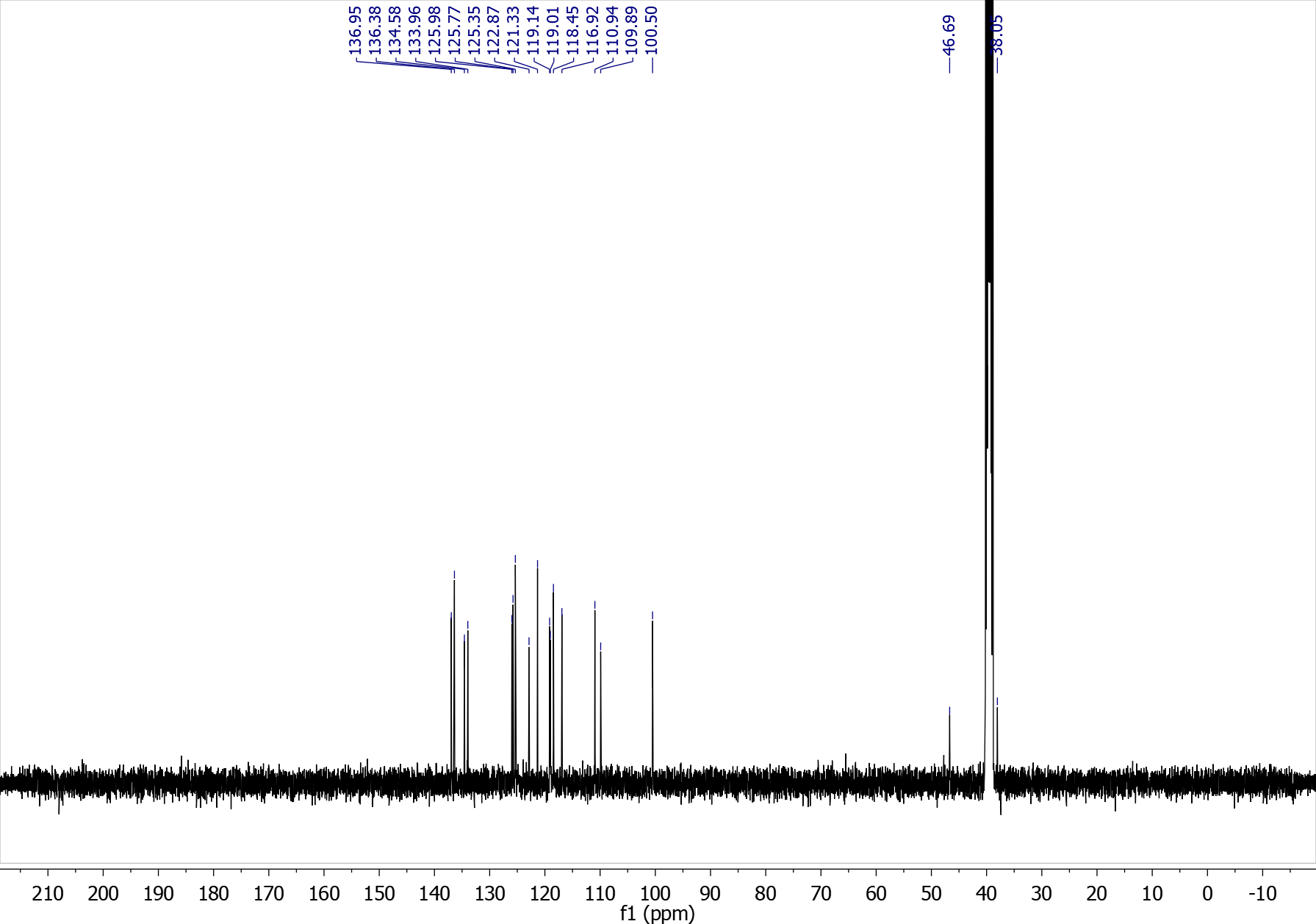


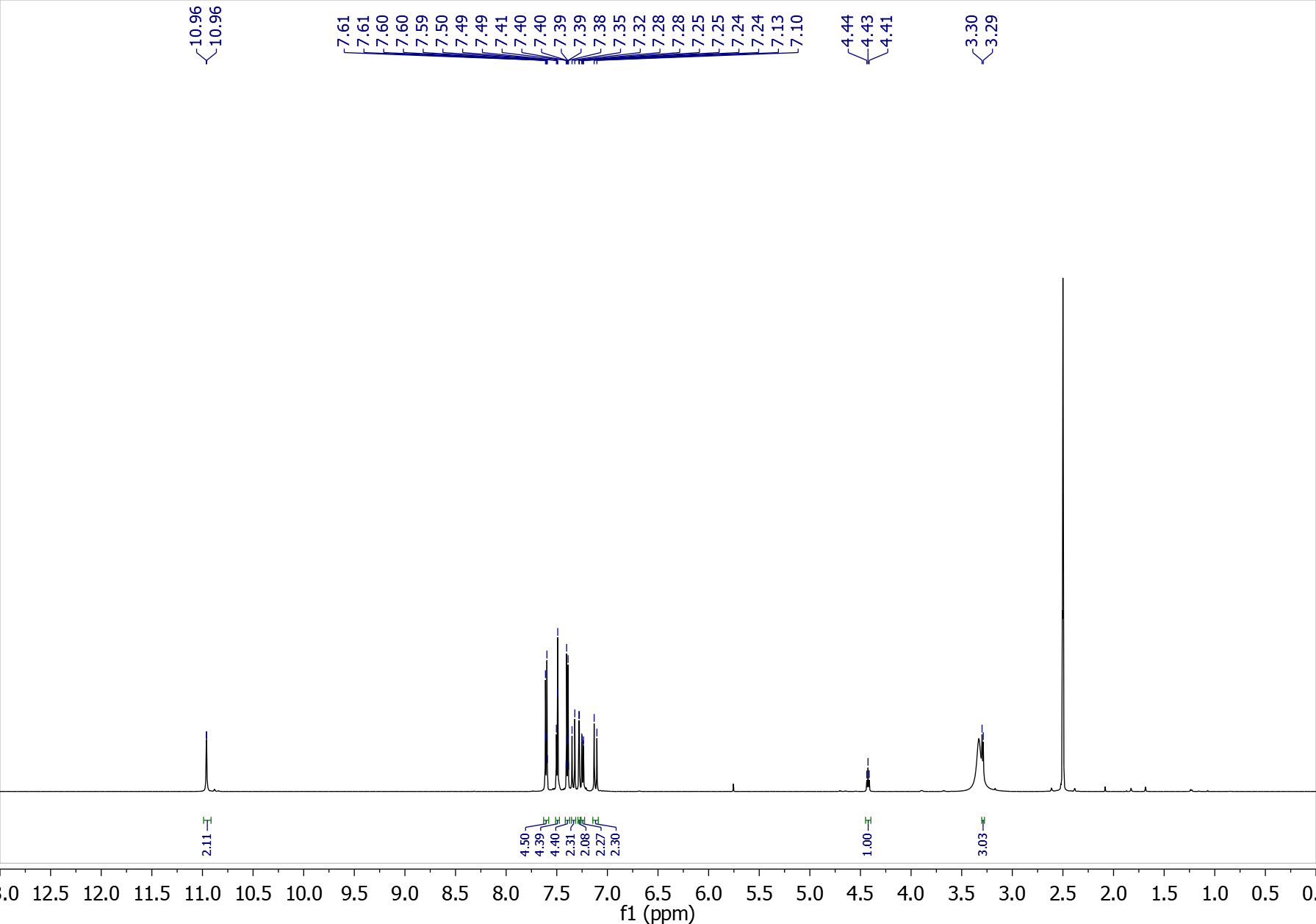

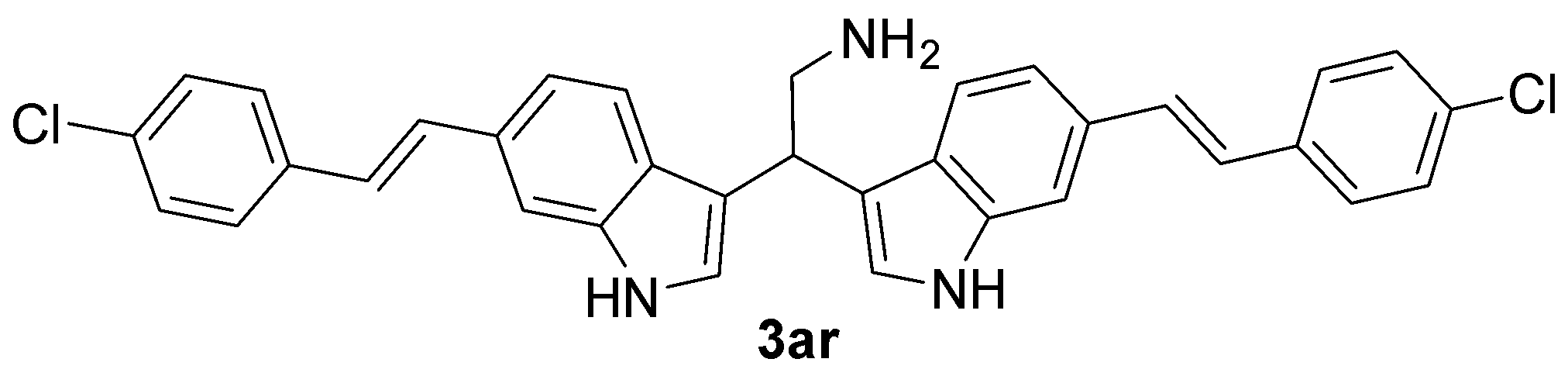


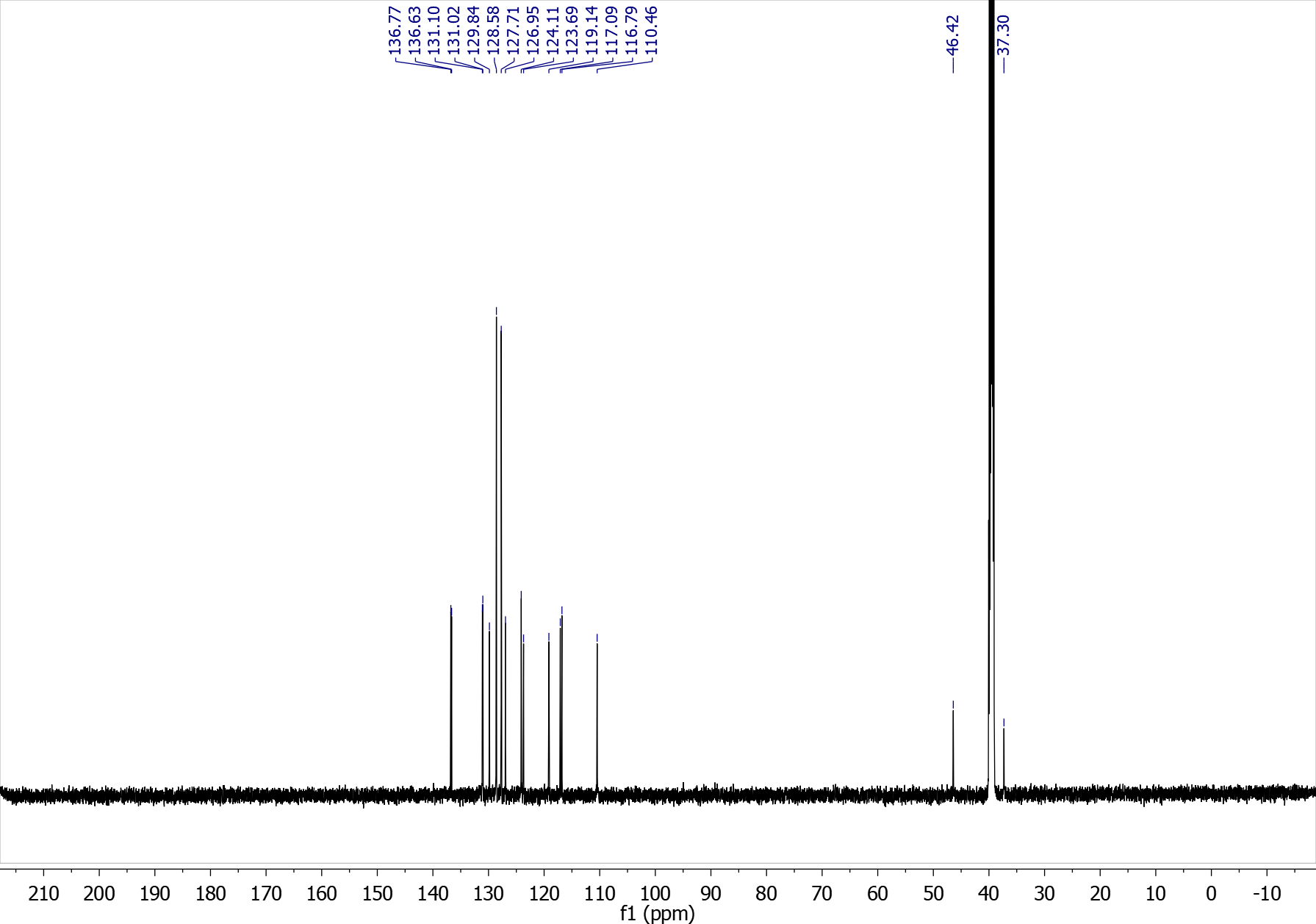


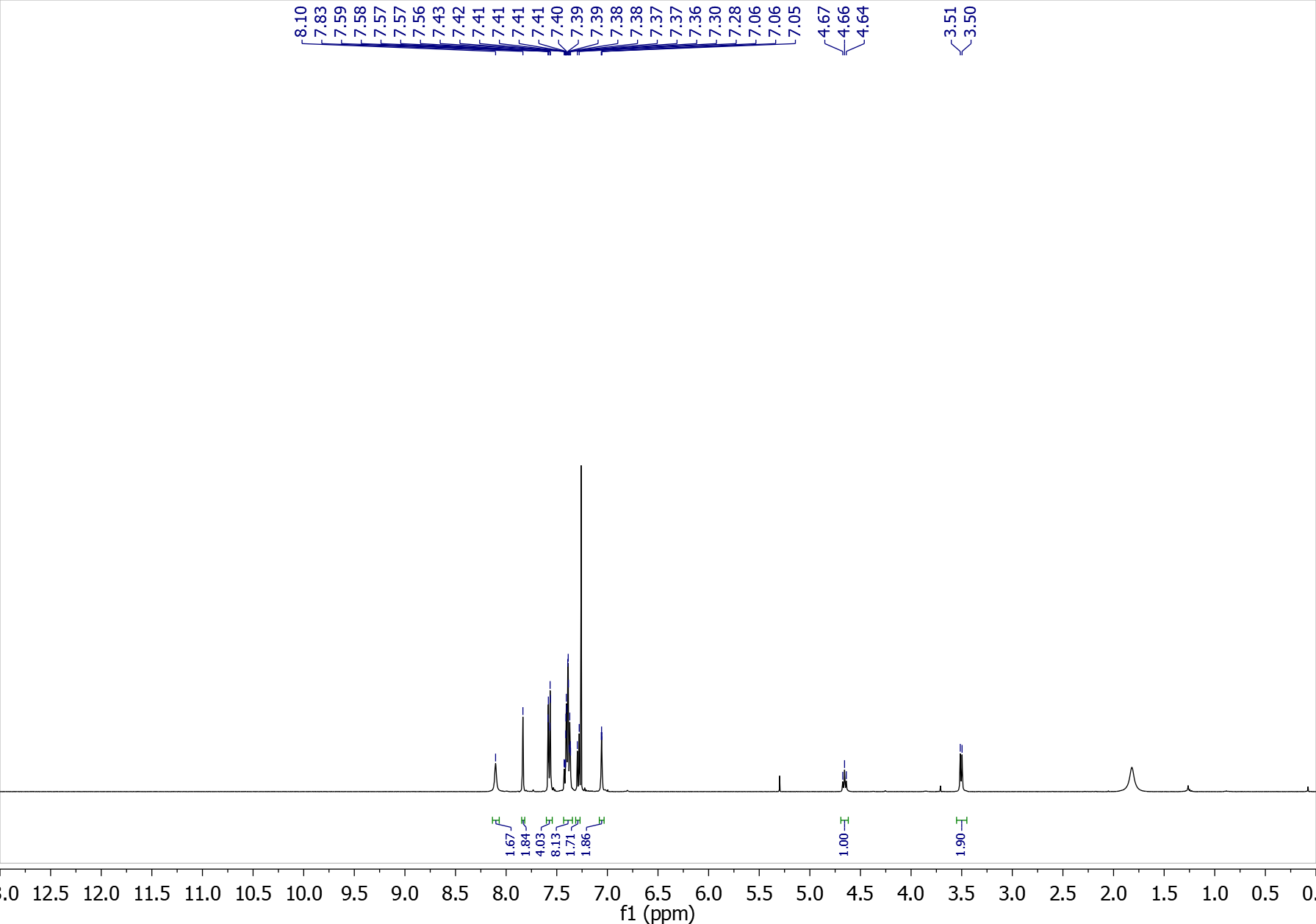

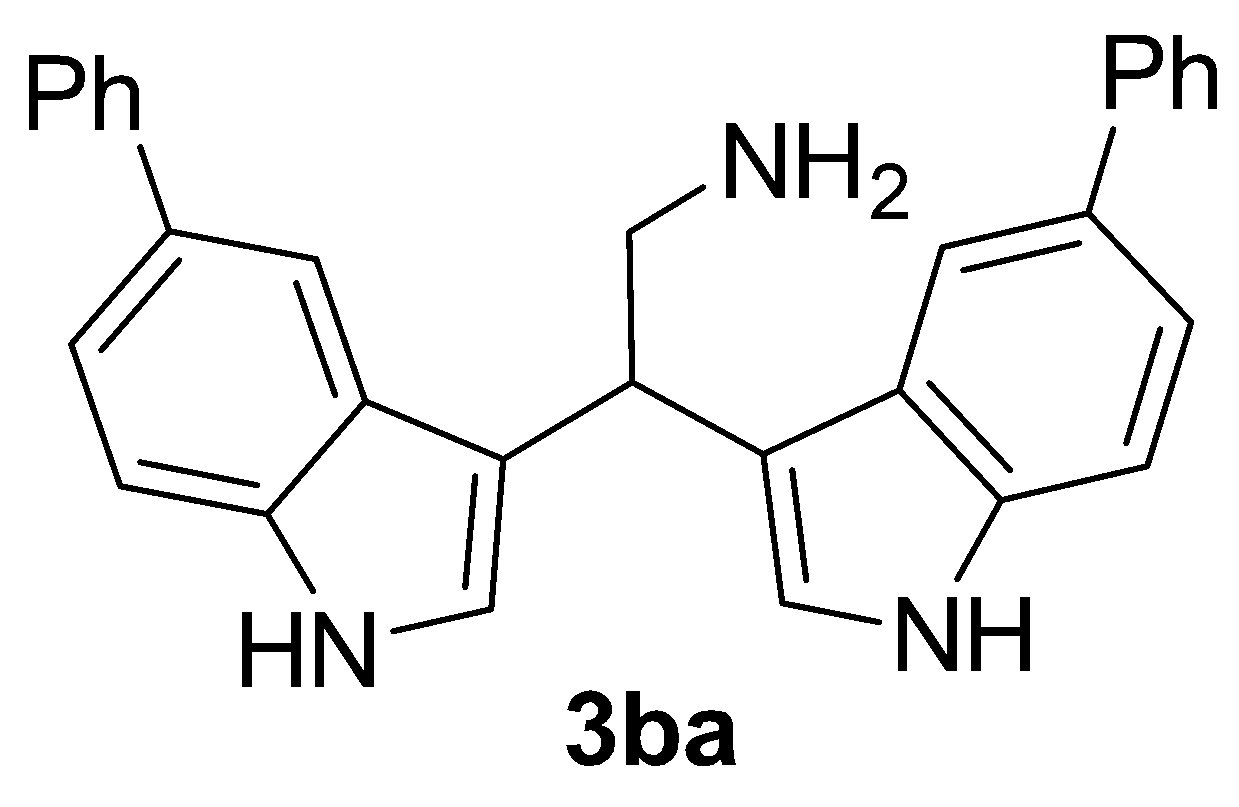


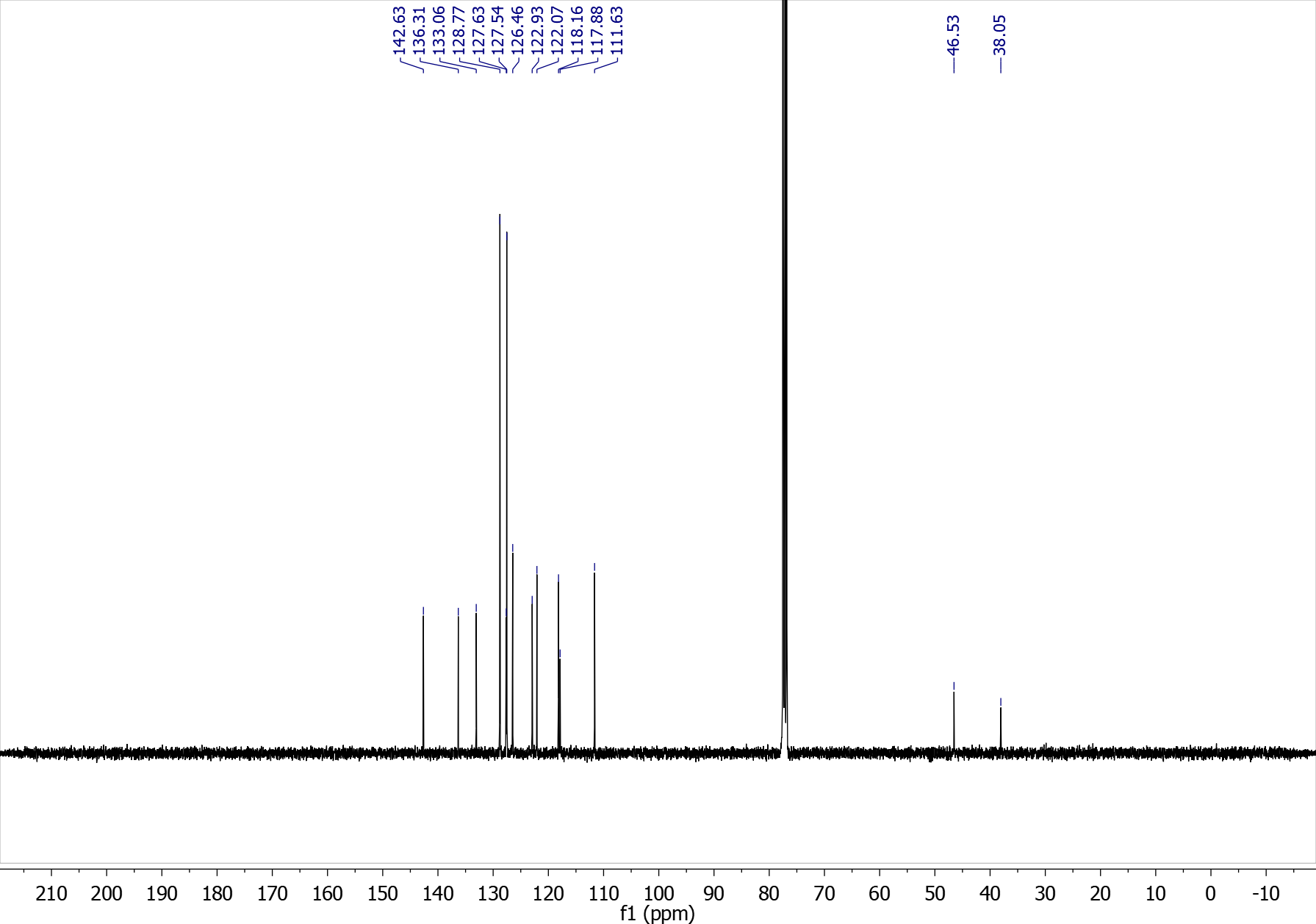


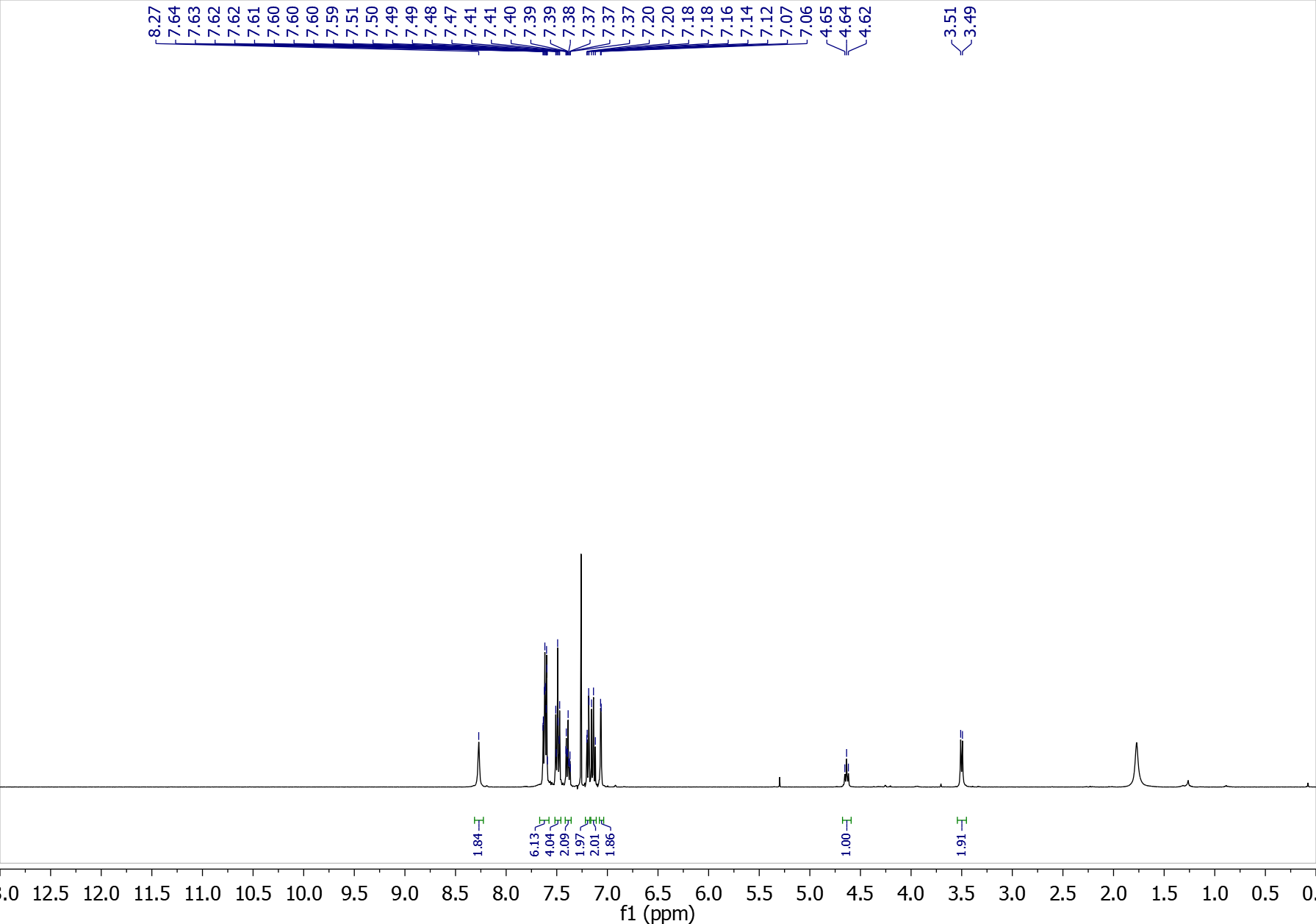

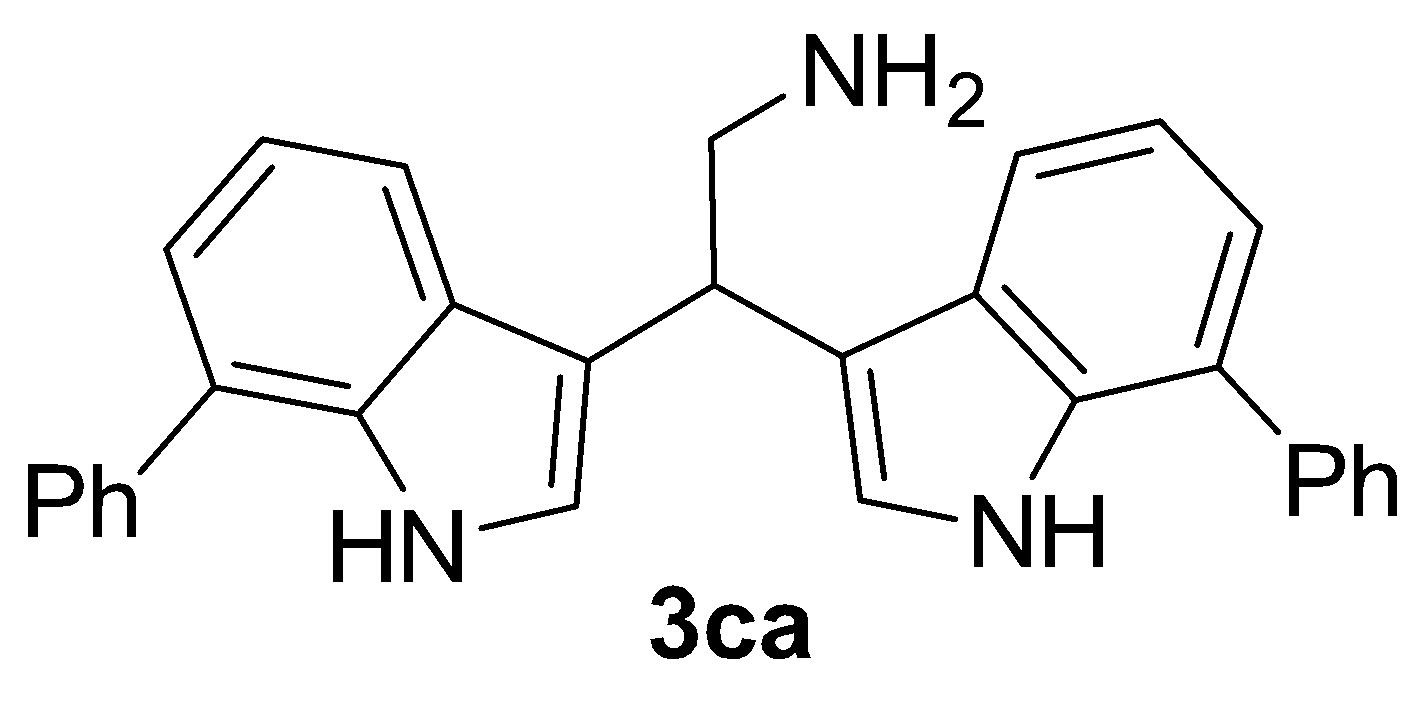


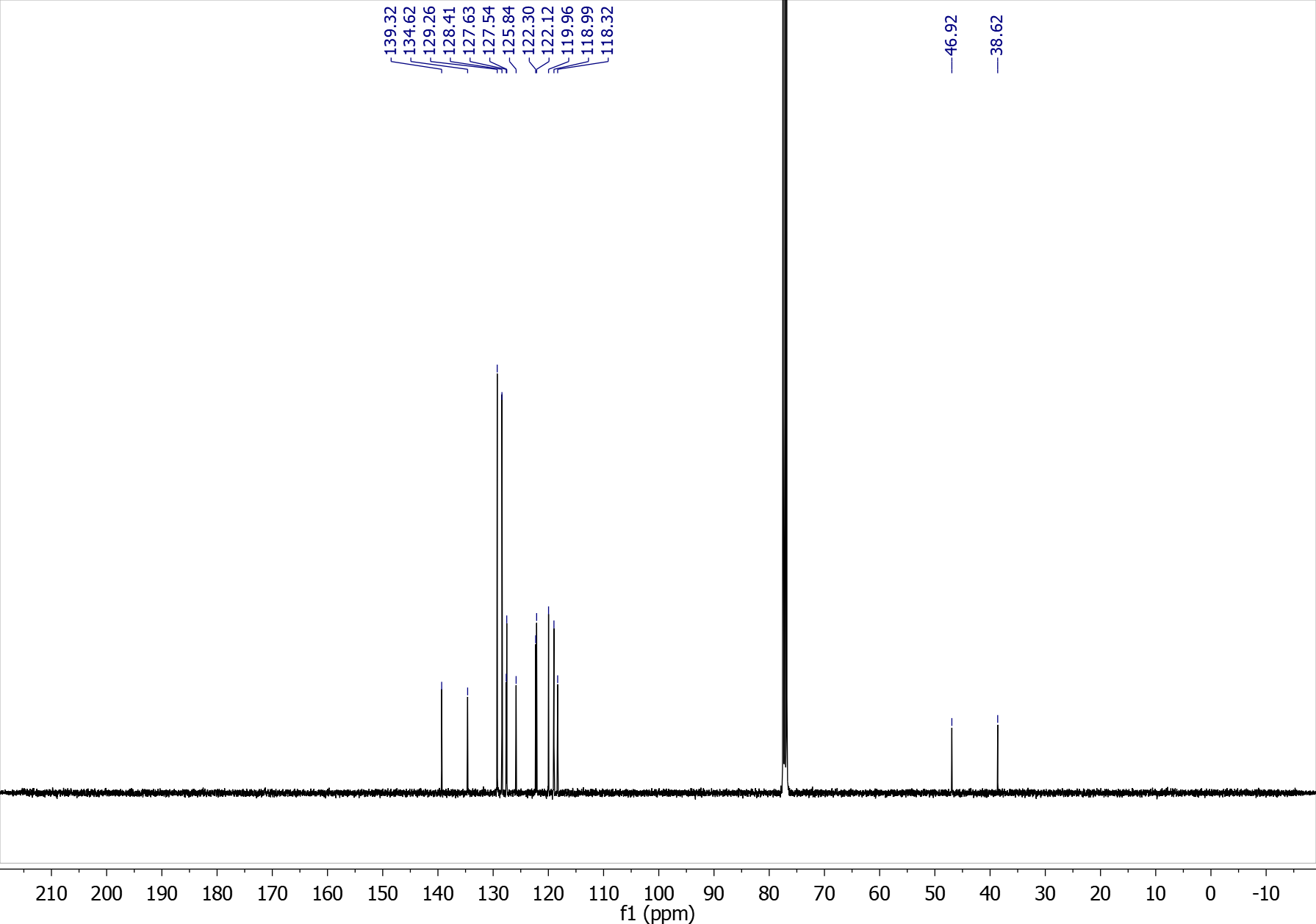


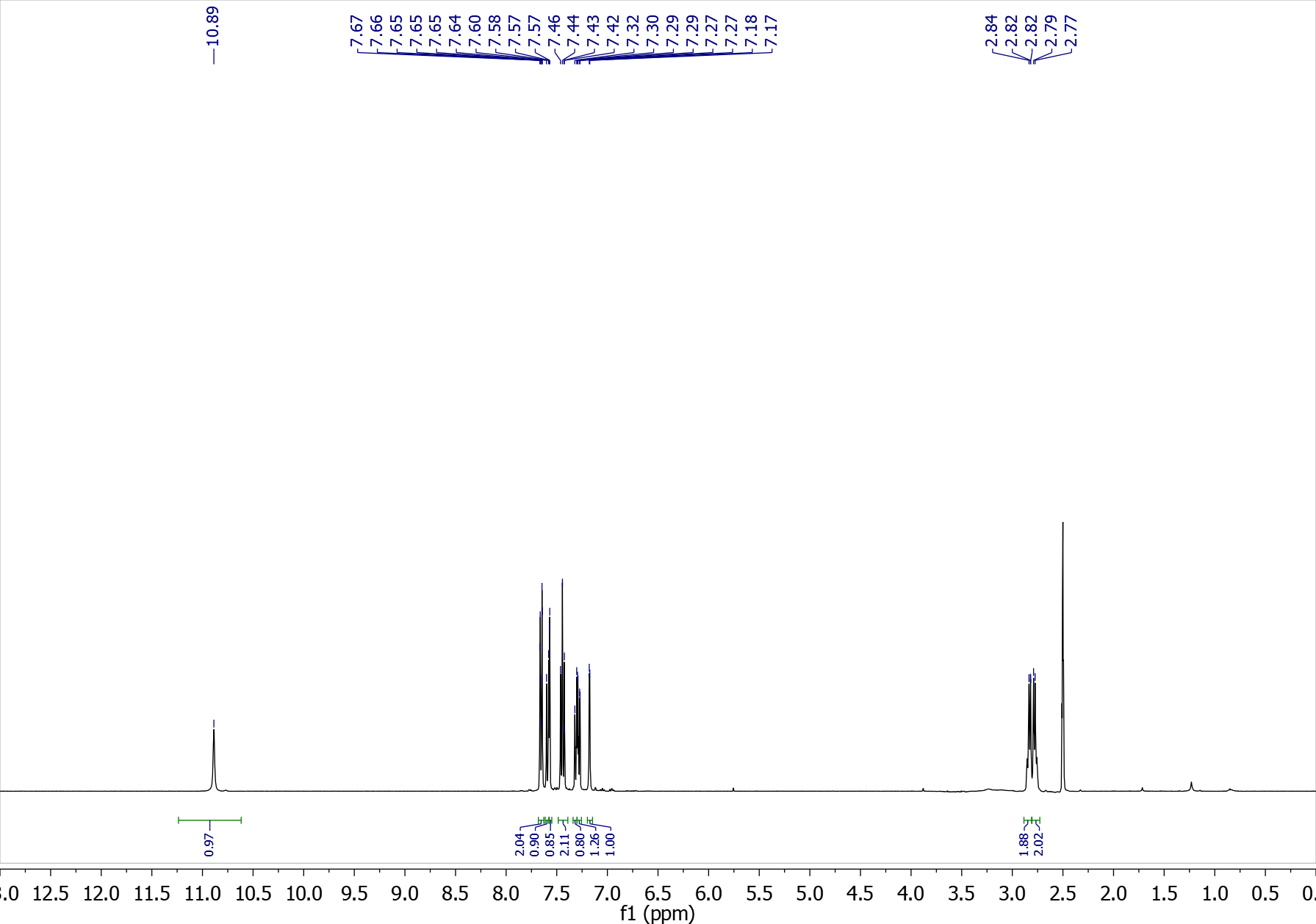

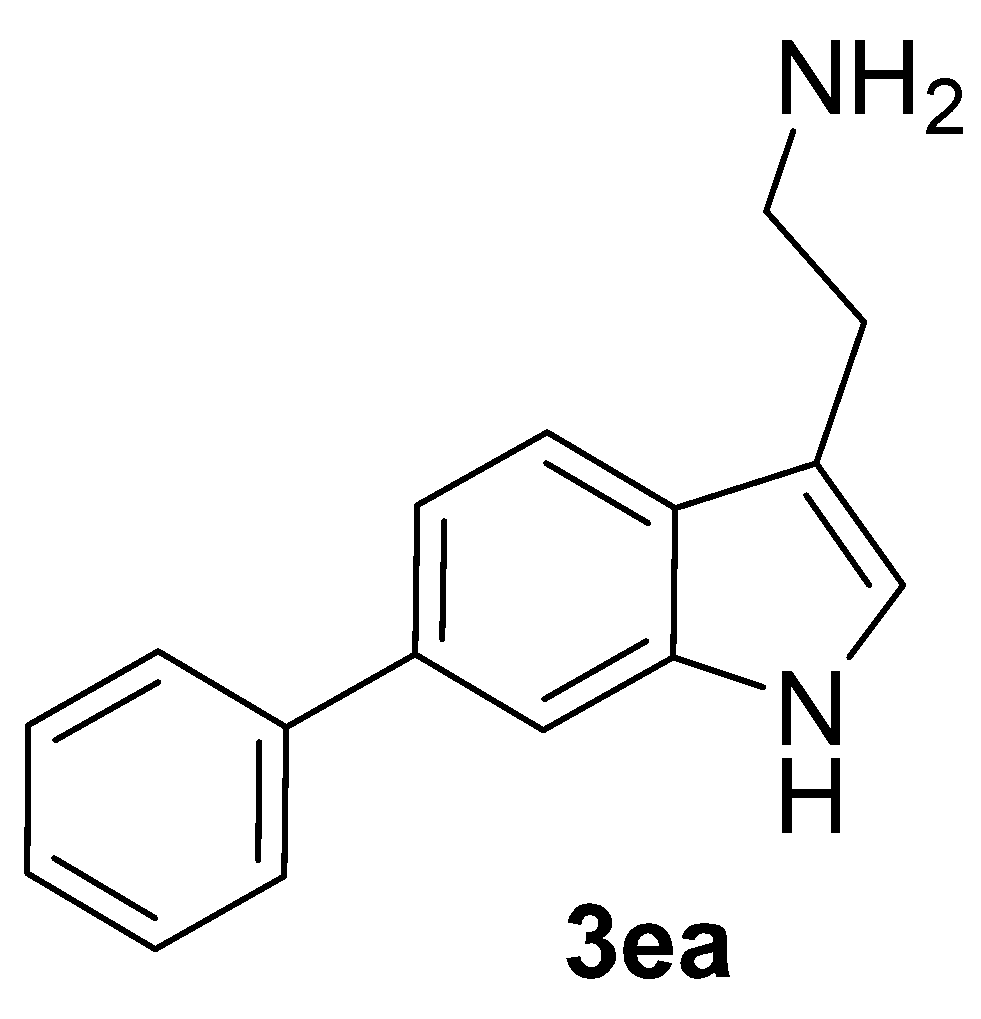


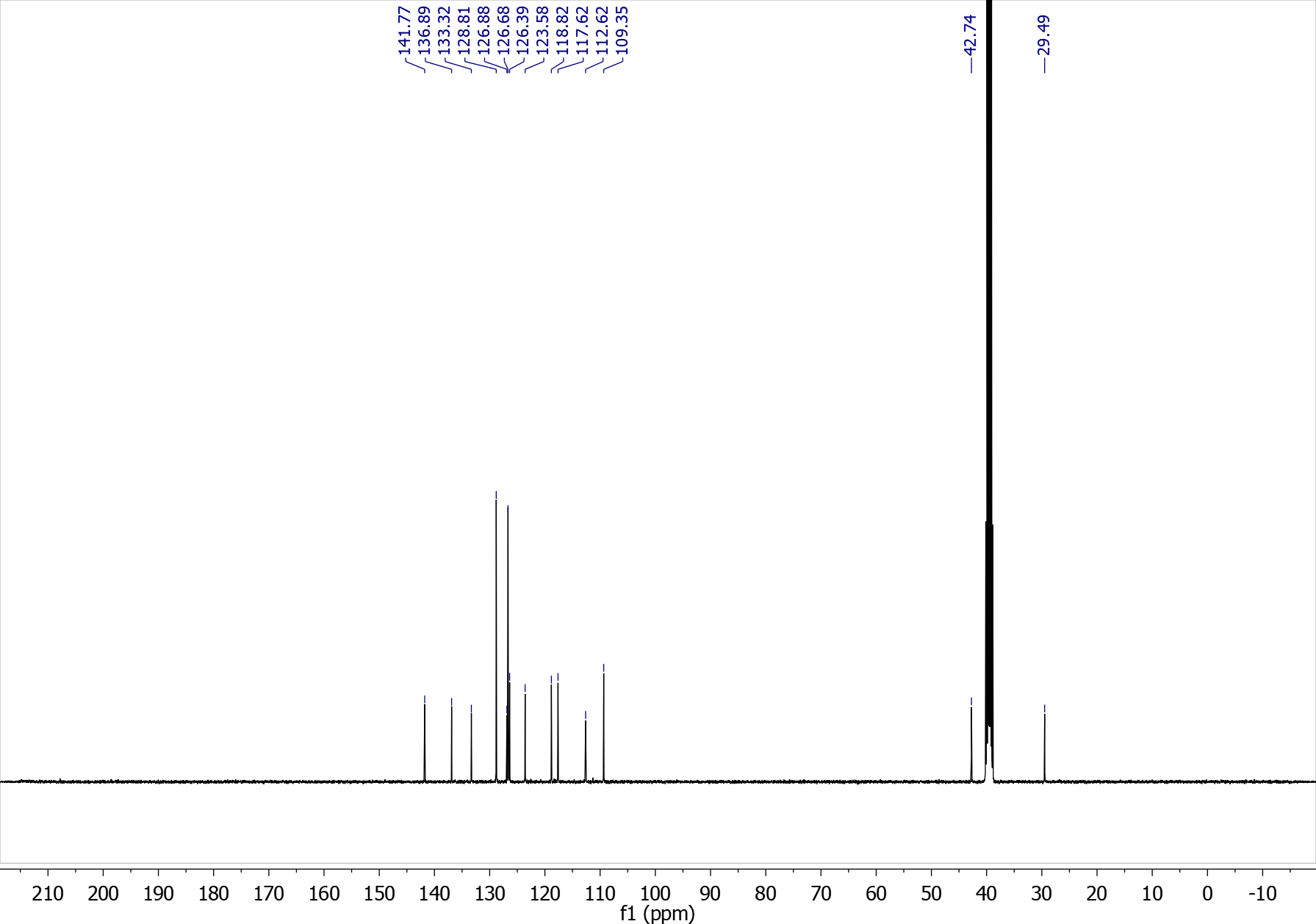


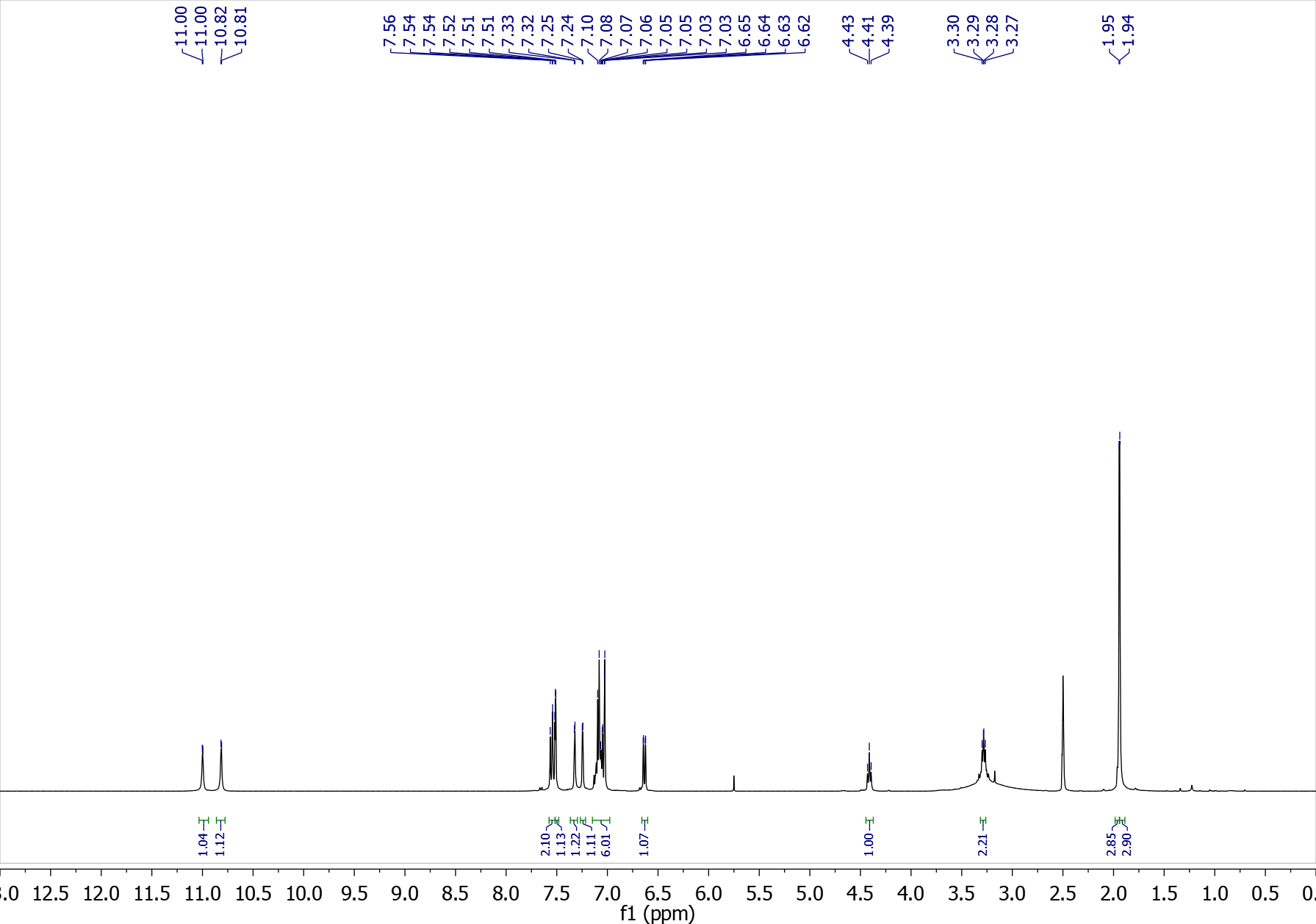

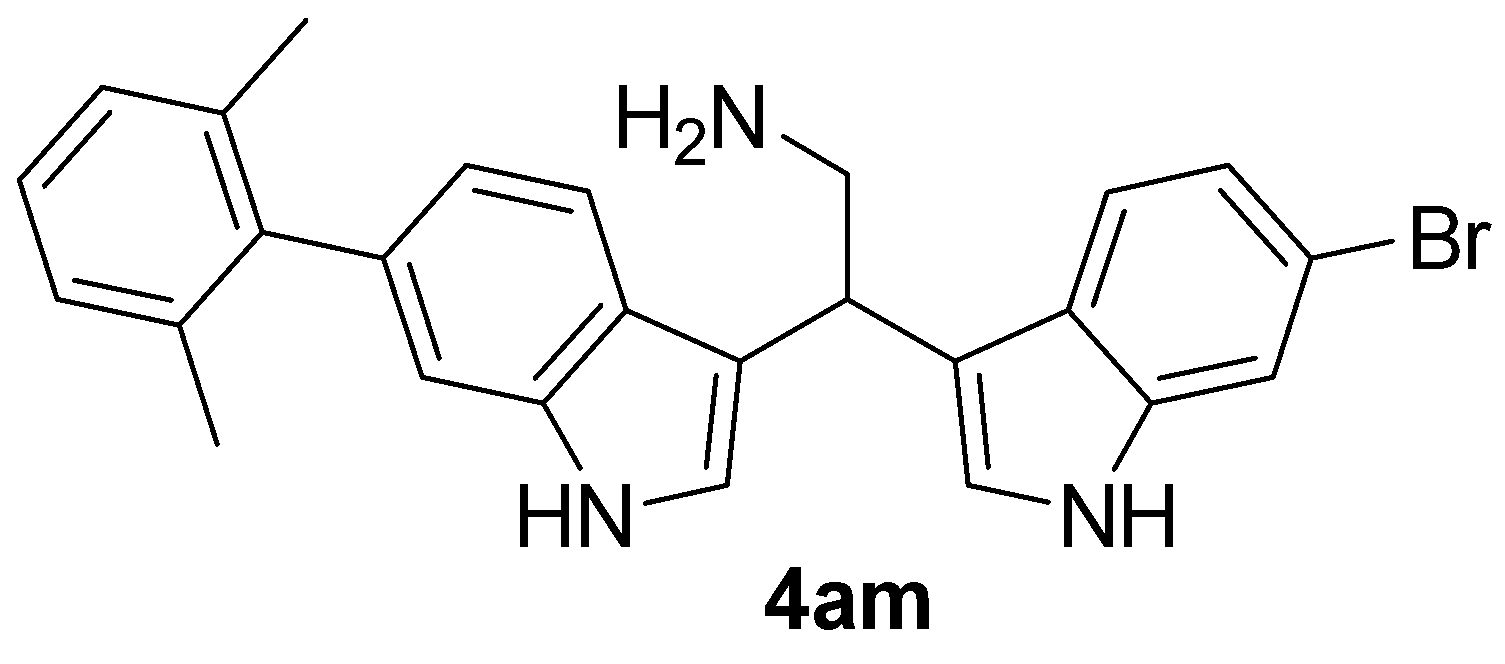


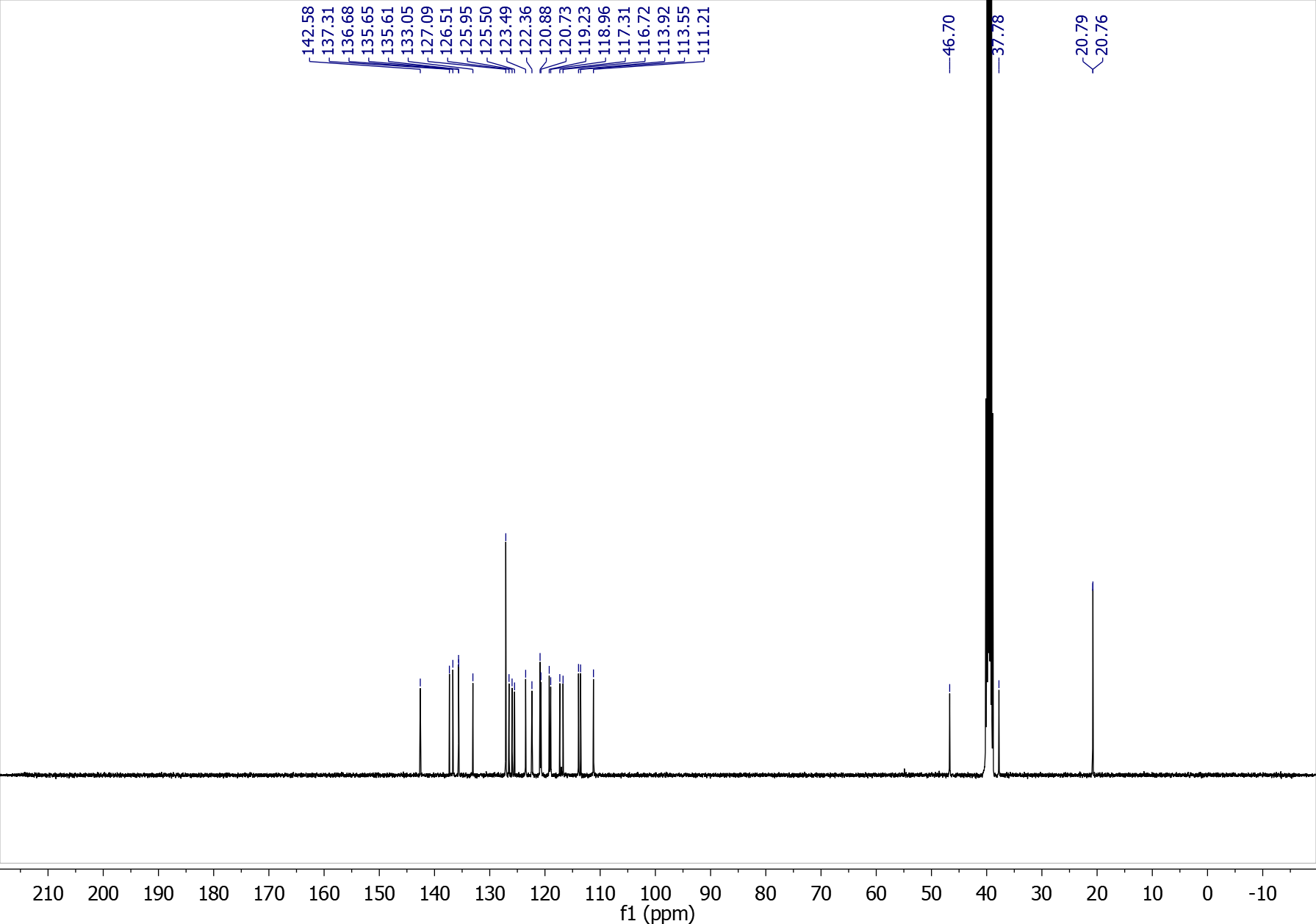


1. **Dose-response curves of 3af; 3ca and 3aq on *L. infantum* promastigotes**


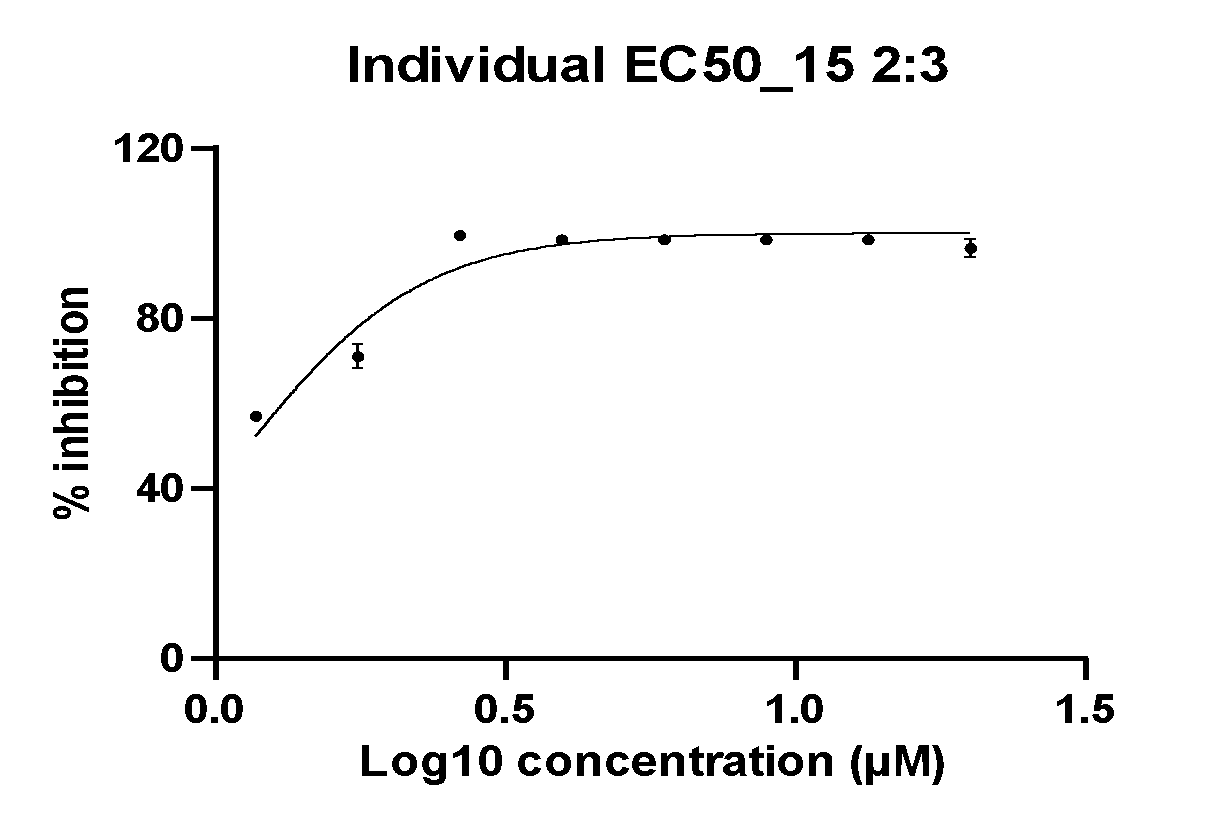


**3af**


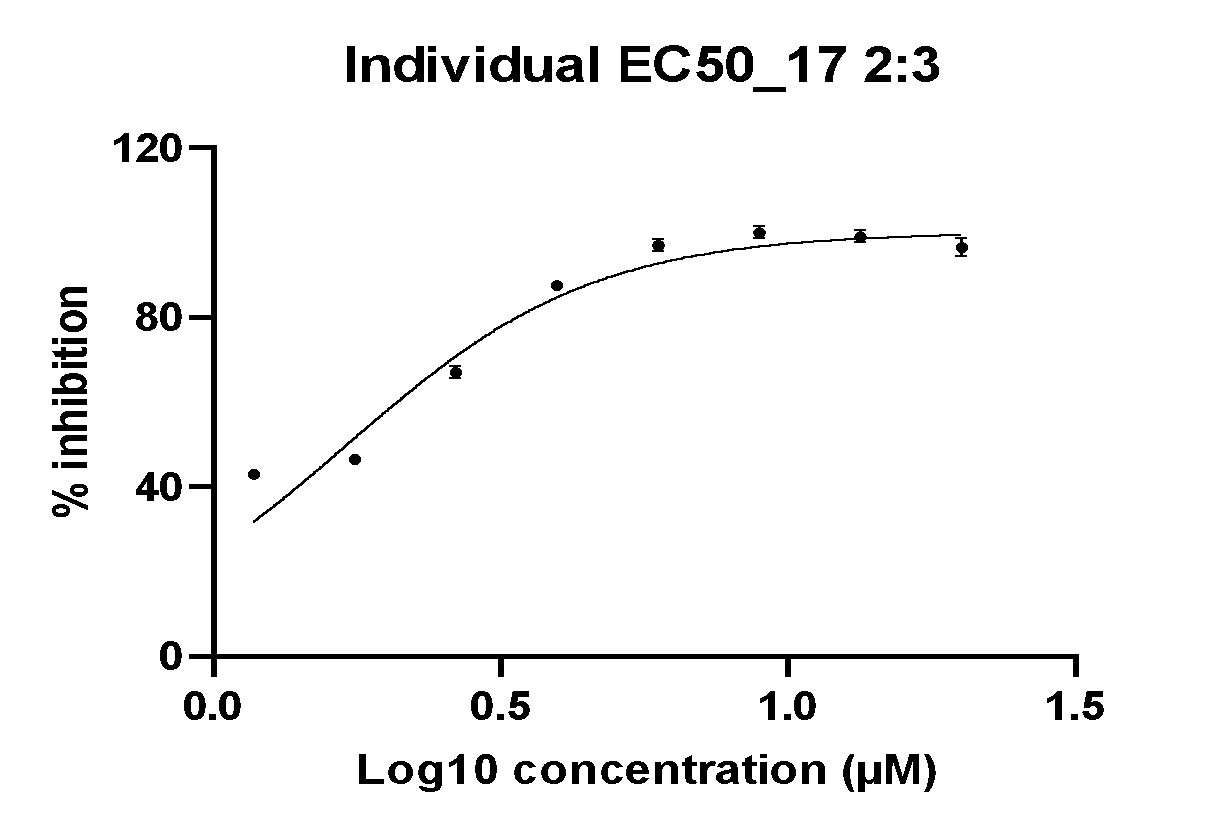


**3ca**


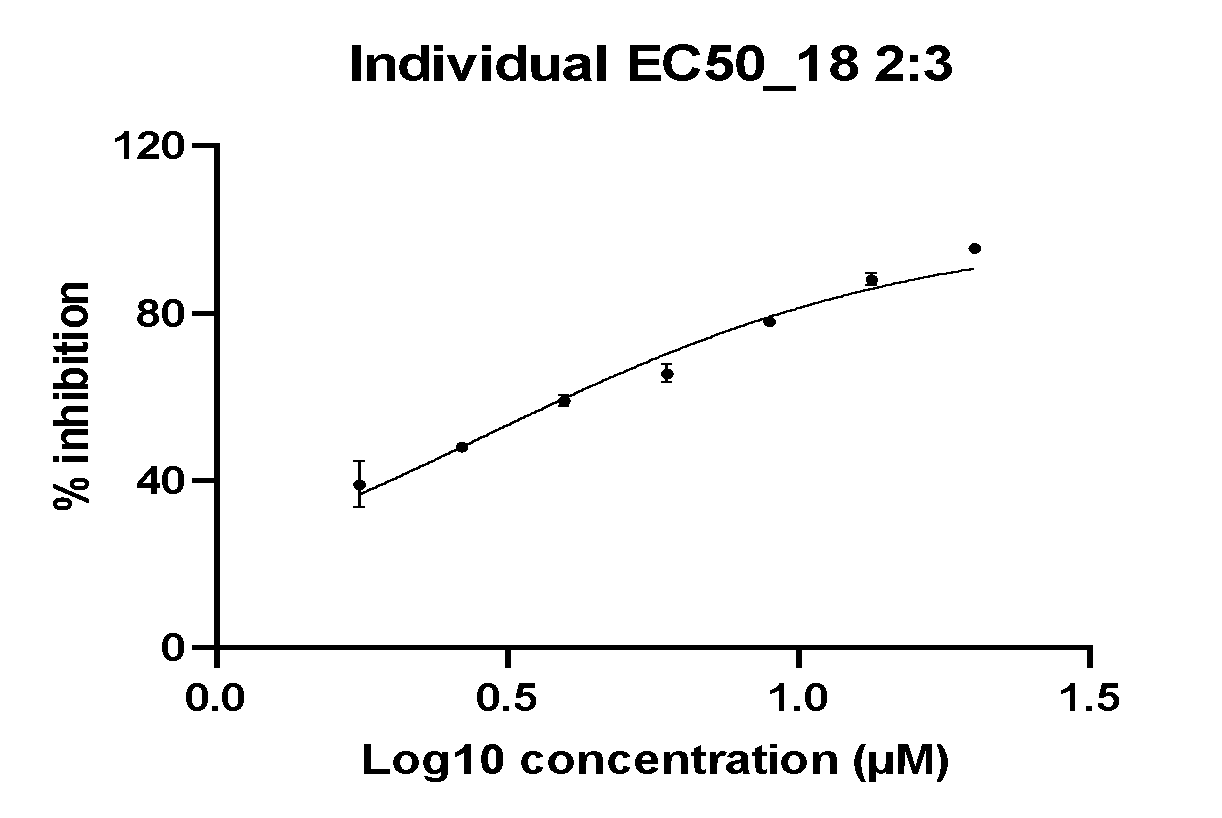


**3aq**

**Figure S8** – Dose-response curves of **3af**; **3ca** and **3aq** on *L. infantum* promastigotes. The curves represent the % inhibition of *L. infantum* parasites at increasing concentrations of each compound.

1. **Dose-response curves of 3af; 3ca and 3aq on THP-1 cells**

**3af**


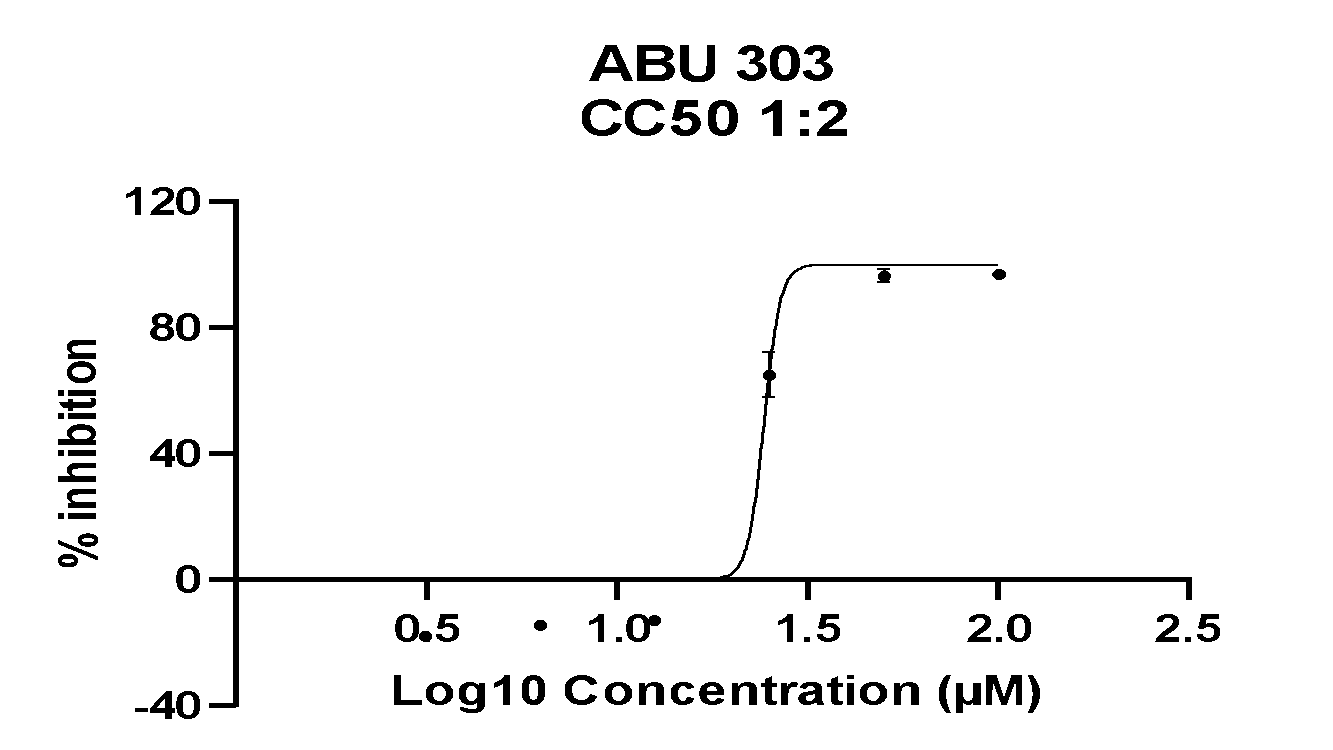


**3ca**


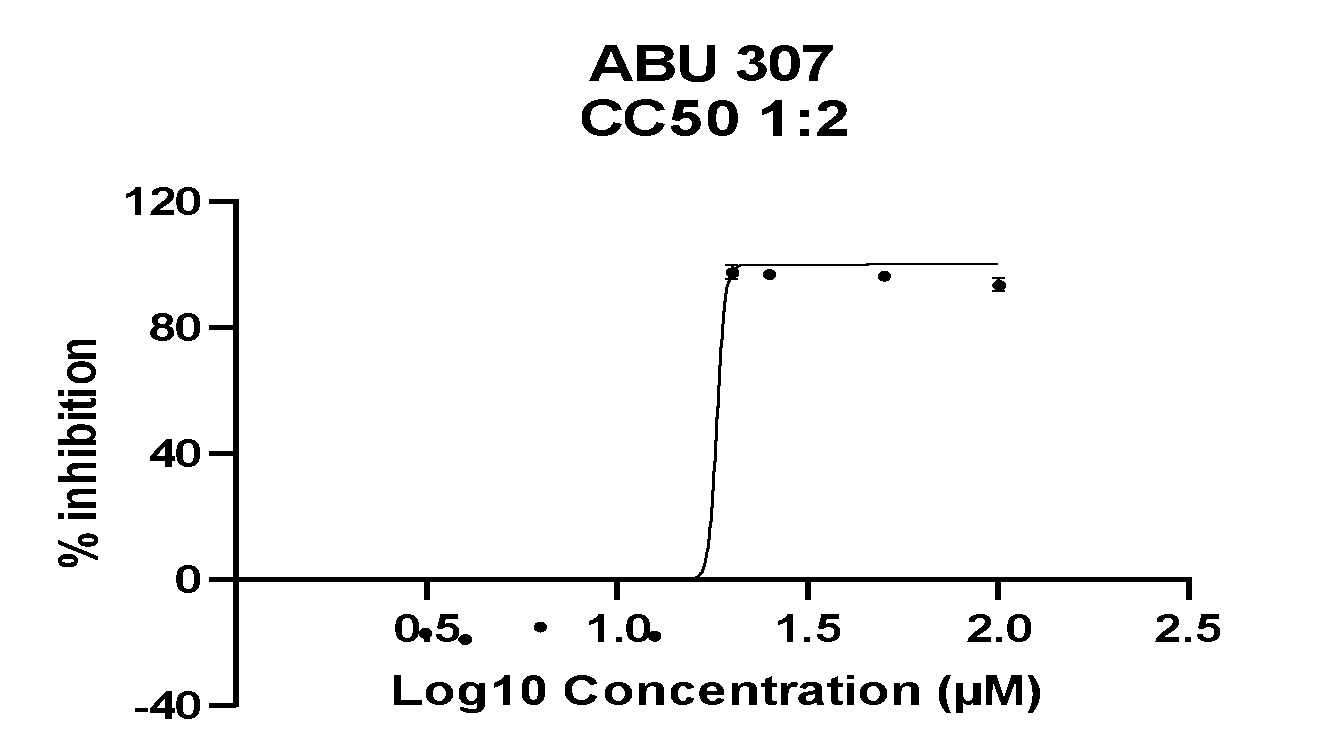


**3aq**


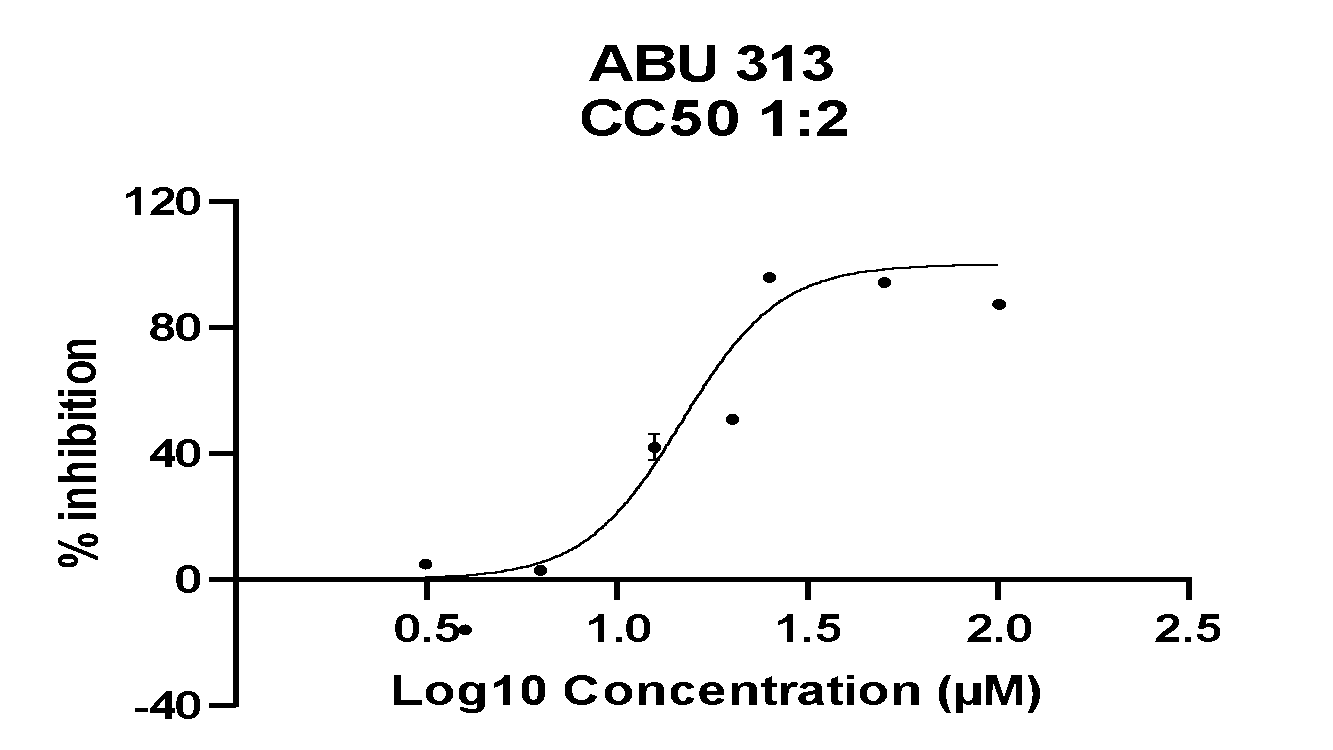


**Figure S9** – Dose-response curves of **3af**; **3ca** and **3aq** on THP-1 cells. The curves represent the % inhibition of THP-1 viability at increasing concentrations of each compound.

1. **Representative phase-contrast images of THP-1 cells infected and treated with compounds 3af, 3aq, 3ca, and miltefosine**

**Figure S10** – Representative phase-contrast images of THP-1 cells infected and treated with compounds **3af**, **3aq**, **3ca**, and miltefosine at the indicated concentrations. Scale bar 100 µm.

1. **References**

[1] M. Mari, A. Tassoni, S. Lucarini, M. Fanelli, G. Piersanti, G. Spadoni, *Eur. J. Org. Chem*. **2014**, 3822.

[2] R. W. Schumachert, B. S. Davidson, *Tetrahedron* **1999**, *34*, 935.

[3] T. Iwamoto, C. Okuzono, L. Adak, M. Jinc, M. Nakamura, *Chem. Commun*. **2019**, *55*, 1128.

[4] A. Diotallevi, L. Scalvini, G. Buffi, Y. Pérez-Pertejo, M. De Santi, M. Verboni, G. Favi, M. Magnani, A. Lodola, S. Lucarini, L. Galluzzi, *ACS Omega* **2021**, *6*, 35699.

[5] A. Centanni, A. Diotallevi, G. Buffi, D. Olivieri, N. Santarém, A. Lehtinen, J. Yli-Kauhaluoma, A. Cordeiro-da-Silva, P. Kiuru, S. Lucarini, L. Galluzzi, *PLoS One*, **2024**, *19*, e0301901.
